# Comparative evaluation of multimarker algorithms for early-stage HCC detection in multicenter prospective studies^☆^

**DOI:** 10.1016/j.jhepr.2024.101263

**Published:** 2024-11-08

**Authors:** Jinlin Hou, Thomas Berg, Arndt Vogel, Teerha Piratvisuth, Jörg Trojan, Enrico N. De Toni, Masatoshi Kudo, Katarina Malinowsky, Peter Findeisen, Johannes Kolja Hegel, Wenzel Schöning, Kairat Madin, Konstantin Kroeniger, Henry Lik-Yuen Chan, Ashish Sharma

**Affiliations:** 1 Hepatology Unit and Department of Infectious Diseases, Nanfang Hospital, Southern Medical University, Guangzhou China; 2 Division of Hepatology, Department of Medicine II, University of Leipzig Medical Center, Leipzig, Germany; 3 Department of Gastroenterology, Hepatology and Endocrinology, Medizinische Hochschule Hannover, Hannover, Germany (At the time of analysis); 4 Division of Gastroenterology and Hepatology, Toronto General Hospital, Toronto, Canada; 5 Medical Oncology, Princess Margaret Cancer Centre, Toronto, Canada; 6 Division of Gastroenterology and Hepatology Department of Medicine, Prince of Songkla University, Hat Yai, Thailand; 7 Department of Gastroenterology, Goethe Universitaet Frankfurt, Frankfurt, Germany; 8 Department of Medicine II, University Hospital, Ludwig Maximilian University of Munich, Munich, Germany; 9 Department of Gastroenterology and Hepatology, Kindai University, Osaka, Japan; 10 Department of Biomarker Services, Microcoat Biotechnologie GmbH, Bernried, Germany; 11 MVZ Labor Dr. Limbach & Kollegen, Heidelberg, Germany; 12 Department of Studies, Collaboration and Innovation Management, Labor Berlin Charité Vivantes Services GmbH, Berlin, Germany; 13 Department of Surgery, Universitätsmedizin Berlin, Chirurgische Klinik, Campus Charité Mitte and Campus Virchow-Klinikum, Berlin, Germany; 14 Global Study Management, Roche Diagnostics GmbH, Penzberg, Germany; 15 Clinical Algorithms & Biomarker Statistics, Roche Diagnostics GmbH, Penzberg, Germany; 16 Department of Internal Medicine, The Chinese University of Hong Kong, Hong Kong Special Administrative Region of China; 17 Clinical Development & Medical Affairs, Roche Diagnostics International AG, Rotkreuz, Switzerland

**Keywords:** Hepatocellular carcinoma, Surveillance, Algorithm, GAAD, GALAD

## Abstract

**Background & Aims:**

We compared the clinical performance of the novel GAAD (gender [biological sex], age, alpha-fetoprotein [AFP], des-gamma carboxyprothrombin [DCP]) and GALAD (gender [biological sex], age, AFP, *Lens culinaris* agglutinin-reactive AFP [AFP-L3], DCP) algorithms to deduce the clinical utility of AFP-L3 for detecting early-stage hepatocellular carcinoma (HCC) from chronic liver disease (CLD).

**Methods:**

An algorithm development study (STOP-HCC-ARP) and clinical validation study (STOP-HCC-MCE) were conducted, recruiting adult participants with HCC (confirmed by radiology or pathology) or CLD in an international, multicenter, case-control design. Serum biomarkers were measured using Elecsys assays (GAAD and GALAD [Cobas]) or μTASWAKO assays (GALAD [μTASWAKO]) while blinded to case/control status.

**Results:**

In STOP-HCC-ARP (algorithm development cohort), 1,006 patients {297 HCC (41.4% early-stage [Barcelona Clinic Liver Cancer {BCLC} 0/A) and 709 CLD} were included. Area under the curve (AUCs) for discriminating between early-stage HCC *vs.* CLD were 91.4%, 91.4%, and 90.8% for GAAD (Cobas), GALAD (Cobas), and GALAD (μTASWAKO), respectively. The clinical validation cohort of STOP-HCC-MCE comprised 1,142 patients, (366 HCC cases [48% early-stage], 468 specificity samples and 302 CLD); AUCs for GAAD (Cobas), GALAD (Cobas), and GALAD (μTASWAKO) for discriminating between early-stage HCC *vs.* CLD were 91.4%, 91.5%, and 91.0%, respectively; AUCs were 94.7–95.0% for all-stage HCC. The GAAD and GALAD algorithms demonstrated similar good performance regardless of disease etiology, presence of cirrhosis, geographical region, and within pan-tumor specificity panels (*p* <0.001).

**Conclusions:**

GAAD (Cobas) demonstrated good clinical performance, similar to GALAD (Cobas and μTASWAKO) algorithms, in differentiating HCC and CLD controls, across all disease stages, etiologies, and regions; therefore, AFP-L3 may have a negligible role in GALAD for HCC surveillance.

**Impact and implications:**

To improve the detection of early-stage hepatocellular carcinoma (HCC) from benign chronic liver disease (CLD), algorithms combining demographic characteristics and serum biomarkers, such as GAAD and GALAD, have been developed. GAAD combines gender (biological sex), age, alpha-fetoprotein (AFP), des-gamma carboxy-prothrombin (DCP); GALAD combines the same characteristics and biomarkers as GAAD with the addition of *Lens culinaris* agglutinin-reactive AFP (AFP-L3). Changing disease etiologies and treatment paradigms have raised questions regarding the utility of AFP-L3 in HCC surveillance. Our work demonstrates that the GAAD (Cobas) algorithm demonstrated good clinical performance and was as sensitive and specific as the GALAD (Cobas) and GALAD (μTASWAKO) algorithms in differentiating HCC and CLD controls, across all disease stages, etiologies, and geographical regions; therefore, AFP-L3 may have a negligible role in HCC detection. Our study provides supporting evidence that in participants with CLD undergoing guideline-directed HCC surveillance, the GAAD (Cobas) algorithm may be used as an effective method for the detection of HCC, potentially resulting in improved patient outcomes.

## Highlights

• Algorithms combining demographics and serum biomarkers may improve detection of early-stage HCC from CLD.
• GAAD and GALAD algorithms demonstrated comparable good performance in differentiating HCC from CLD.
• GAAD and GALAD performed comparably across disease stages, etiology and geography.
• AFP-L3 may have a negligible role in GALAD for HCC surveillance.
• GAAD may be a time- and cost-efficient tool for detecting early-stage HCC.

## Graphical abstract

**Figure:**
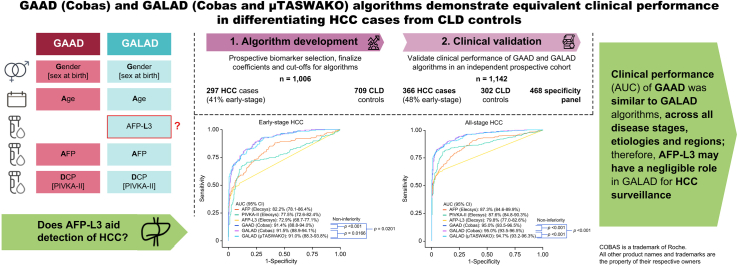

## Introduction

Early hepatocellular carcinoma (HCC) surveillance is essential to improve clinical outcomes.^1^^,^^2^ Therefore, surveillance programs, including ultrasonography (USG) every 6 months with or without alpha-fetoprotein (AFP) testing, are recommended to screen at-risk patients.^1^^,^[3], [4], [5], [6] Risk factors for developing HCC include cirrhosis, hepatitis B and C virus (HBV, HCV) infection, metabolic dysfunction-associated steatotic liver disease (MASLD), and alcoholic-related liver disease (ALD).^2^

Although surveillance programs are guideline-recommended, the combination of USG + AFP may only identify up to 70% of patients with early-stage HCC.^1^^,^^4^^,^^5^^,^^7^ In addition to AFP, other HCC-specific serum biomarkers, such as protein induced by vitamin K absence or antagonist-II (PIVKA-II) and *Lens culinaris* agglutinin-reactive AFP (AFP-L3), have been identified.^8^ However, when used individually, serum biomarkers demonstrate inadequate sensitivity and accuracy for HCC diagnosis.^1^^,^[3], [4], [5], [6]^,^^8^ Notably, a recent Asia-Pacific consensus paper advised that PIVKA-II in combination with AFP (and USG) shows potential benefit for HCC detection especially in those with small and AFP-negative tumors, which are predominantly early-stage disease.[9], [10], [11]

To further improve the detection of early-stage HCC from benign chronic liver disease (CLD), algorithms combining demographic characteristics and serum biomarkers have also been developed.^8^^,^[12], [13], [14] Notably, the GALAD score, combining gender (biological sex) and age plus a three-serum biomarker panel (AFP-L3, AFP, and PIVKA-II), has demonstrated good clinical performance for the differentiation of HCC and CLD, superior to that of single biomarkers in multiple case-control and prospective phase II/III biomarker studies.^8^^,^[12], [13], [14], [15], [16] Although AFP-L3 is included in GALAD, the first algorithm development study reported odds ratios of 1.05 and 1.04 for AFP-L3 in the discovery and validation datasets, respectively, with near zero coefficients; thus, AFP-L3 may contribute negligibly to the GALAD algorithm.^14^

Years later, the role and contribution of AFP-L3 in HCC detection remains controversial as a standalone assay and component of GALAD owing to evolving disease etiologies and antiviral treatment paradigms. Viral hepatitis-related HCC has plateaued globally, whereas ALD and MASLD-related HCC incidence and mortality have increased,[17], [18], [19] highlighting a need to closely examine the utility of AFP-L3 and GALAD in the evolving high-risk population. Because of advances in antiviral HBV/HCV therapies leading to improved liver function, post-treatment AFP levels stay normalized, rendering high specificity to AFP for HCC surveillance in patients with chronic inflammatory background,^20^ potentially making AFP-L3 usage obsolete.^21^ Therefore, novel Elecsys-based GAAD (gender [biological sex], age, AFP, DCP [des-gamma carboxyprothrombin {PIVKA-II}]) and GALAD algorithms (Roche Diagnostics International Ltd, Rotkreuz, Switzerland) have been developed. The GAAD algorithm does not include AFP-L3 and demonstrated good clinical performance in HCC and benign CLD differentiation.^22^

In this study, we compared the clinical performance of the GAAD and GALAD algorithms to deduce the clinical utility of AFP-L3 in the context of disease etiology shift and current treatment patterns in two large-scale prospective studies.

## Experimental procedures

### Study population

**Fig. 1:**
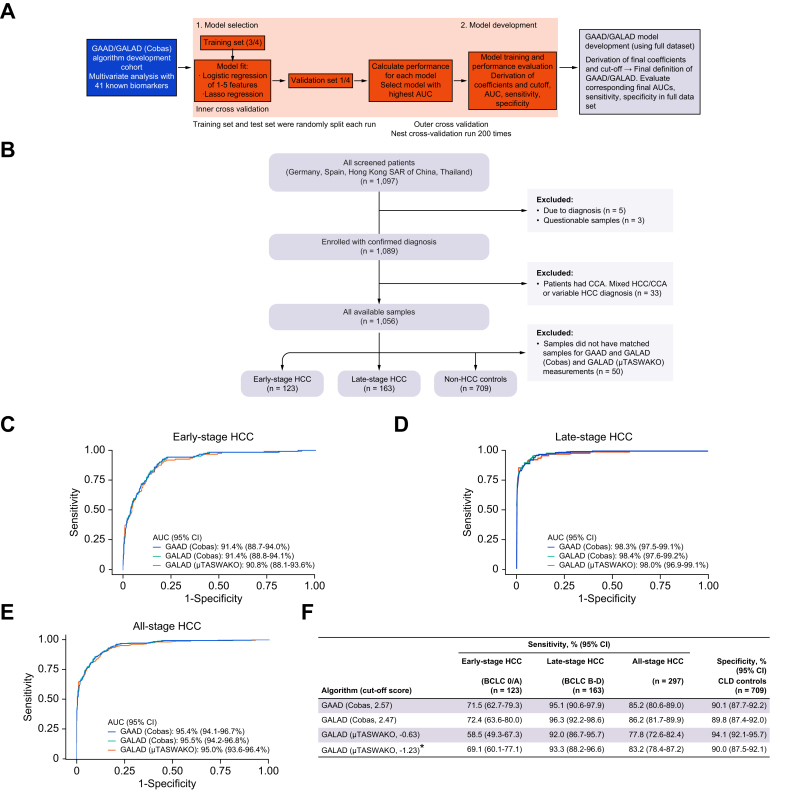
Development, clinical evaluation, and performance of GAAD (Cobas), GALAD (Cobas), and GALAD (μTASWAKO) algorithmic scores in STOP-HCC-ARP. Algorithm development strategy (A) and study disposition (B); clinical performance in differentiating early-stage (C); late-stage (D) and all-stage HCC (E) from CLD controls. Sensitivities and specificities are shown in table (F). ∗Cut-off value corresponds to matching GAAD (Cobas) speciﬁcity of 90%. AUC, area under the curve; BCLC, Barcelona Clinic Liver Cancer; CCA, cholangiocarcinoma; CLD, chronic liver disease; GAAD, gender (biological sex), age, AFP, DCP (PIVKA-II); GALAD, gender (biological sex), age, AFP-L3, AFP, DCP (PIVKA-II); HCC, hepatocellular carcinoma.

Two independent, international, multicenter, prospective cohort studies were enrolled using a case-control design in participants aged ≥18 years, comprising cohorts of HCC (early-stage [Barcelona Clinic Liver Cancer {BCLC} tumor stage 0/A], late-stage [BCLC tumor stage B–D], and all-stage), and a benign CLD control. The STOP-HCC-ARP algorithm development study involved seven clinics across Germany, Spain, Thailand, and Hong Kong Special Administrative Region of China (SAR) between 2014 and 2016; the study design and biomarker selection methodology have been previously described (Fig. 1A).^8^ The STOP-HCC-MCE clinical validation study involved 10 clinics from the People’s Republic of China, Hong Kong (SAR), Germany, Thailand, and Japan from 2017 to 2022. The study data were included in clinical performance analysis and a specificity panel.

Eligible HCC patients had a first-time HCC diagnosis, confirmed either by radiology according to international guidelines (USG, or either a ≥1 cm lesion showing arterial-phase hyperenhancement in combination with washout appearance and/or capsule by quadruple-phase computed tomography scan or multiphase contrast-enhanced magnetic resonance imaging)^1^^,^^5^^,^^23^ or by positive pathology within 6 months of enrollment. Briefly, the BCLC staging system was used to categorize patients with HCC into one of the following disease stages: very early HCC (stage 0; single nodule ≤2 cm without vascular invasion or extrahepatic spread in an asymptomatic patient with preserved liver function); early HCC (stage A; single nodule or ≥3 nodules <3 cm without macrovascular invasion or extrahepatic spread in an asymptomatic patient [performance status {PS} 0]); intermediate HCC (stage B; multifocal HCC with no vascular invasion or extrahepatic spread in an asymptomatic patient with preserved liver function [PS 0]); advanced HCC (stage C; patients with vascular invasion or extrahepatic spread presenting with PS ≥2 and preserved liver function); end-stage (stage D; major cancer-related symptoms [PS >2] and/or impaired liver function without the option of liver transplant owing to HCC burden or non–HCC-related factors). Eligible CLD controls comprised at-risk patients without HCC confirmed by imaging within the past 12 months, and the presence of cirrhotic or non-cirrhotic liver disease of either viral (HBV/HCV infection) or non-viral (ALD, metabolic dysfunction-associated steatohepatitis or other) etiology undergoing HCC surveillance.

Exclusion criteria was any other cancer (excluding non-melanoma skin cancer), recurrent HCC, HCC treatment, a glomerular filtration rate <60 ml/min/1.73 m^2^, or treatment with anti-vitamin K coagulant therapy. Those in the CLD control group with a hepatic mass (either indeterminate or meeting radiological criteria for HCC) were also excluded.

For specificity panel analysis of STOP-HCC-MCE, diseases included in the specificity panel were grouped into the following categories: (1) other histologically-confirmed malignancies: cholangiocarcinoma, colorectal cancer, pancreatic cancer, gastric/esophageal cancer, gynecological cancers (ovarian, endometrial and cervical), lung cancer, renal cancer, breast cancer; (2) other benign liver diseases: hemangioma, benign liver cysts, other benign liver diseases (hepatocellular adenoma or focal nodular hyperplasia); and (3) benign diseases: rheumatoid arthritis, morbus Crohn, ulcerative colitis, other autoimmune disease (systemic lupus erythematosus and Hashimoto thyroiditis). To note, patients with cholangiocarcinoma and pancreatic cancer were excluded from the analysis.

Ethics committee and institutional review board approvals were obtained for all study sites involved in sample collection. Each participant provided informed consent before enrollment, and local rules regarding informed consent for the subsequent use of collected samples were followed.

### GAAD and GALAD (Cobas) algorithm development

Multivariate analyses were performed to identify the best-performing biomarker panel that could separate early- or all-stage HCC from benign controls using two methods: (1) lasso regression (no fixed panel size) and (2) exhaustive search with logistic regression (fixed panel size [from two to four biomarkers]). Lasso regression optimized the best model by maximizing diagnostic accuracy while minimizing the biomarker number. In an exhaustive search, logistic regression models, based on all possible two to four biomarker combinations, were evaluated and compared. For the two top-performing clinical algorithms (GAAD and GALAD), the full STOP-HCC-ARP dataset was used to train logistic regression models, with an HCC diagnosis (BCLC-stage-independent) used as the predictor variable. After training the models, the GAAD and GALAD cut-offs were determined by calculating the 90% quantile of the score values (using the type 2 method, described previously^24^) from the control patients of STOP-HCC-ARP, which corresponds to the 90% specificity cut-off for the aid in diagnosis of early-stage HCC.

### Biomarker evaluation

Serum samples were collected ≥1 day before any planned procedures requiring general anesthesia and stored at -70 °C at the sample collecting sites. Samples were tested at Microcoat GmbH (Bernried, Germany), with the exception of samples collected in the People’s Republic of China (Elecsys assays were tested at the collection site and μTASWAKO assays were tested at Huashan Hospital, Shanghai, China).

PIVKA-II, AFP, and AFP-L3 serum levels were measured in one run within 3 experimental days using either Elecsys assays on the Cobas e 601 analyzer, or in multiple smaller batches in more than 3 days using μTASWAKO assays on the Fujifilm Micro Total Analysis System μTASWako analyzer owing to lower throughput of the μTASWako analyzer. Sample stability studies were conducted for all assays in accordance with the regulatory guidelines for an *in vitro* diagnostic medical device registration.

Predefined established cut-offs (based on the training cohort) used for the detection of HCC *vs.* benign CLD were: 20 ng/ml for AFP (Elecsys); 2.3 ng/ml for AFP-L3 (Elecsys); 28.4 ng/ml for PIVKA-II (Elecsys); 2.47 (range 0–10) for GALAD (Cobas); 2.57 (range 0–10) for GAAD (Cobas); and –0.63 for GALAD (μTASWAKO).

Additional cut-offs for GALAD (μTASWAKO) were also assessed, corresponding to GAAD (Cobas) specificity of 90%.

### Statistical analysis

The clinical performance of the GAAD and GALAD algorithms, and individual biomarkers alone, were compared using receiver operating characteristic (ROC) analysis, and area under the curve (AUC) values were calculated. For sensitivity and specificity analyses, the derived 95% confidence intervals (CIs) were calculated from the binomial distribution using the Clopper-Pearson method.^25^ Values of *p* comparing non-inferiority of AUCs were calculated using the H0 hypothesis AUC (Test 1) < AUC (Test 2) - 0.01 with a studentized bootstrap approach using N = 1,000 bootstrap replicates.

For the specificity panel in the STOP-HCC-MCE study, *p* values were calculated using binomial tests, with Bonferroni correction for every marker using the cut-offs listed previously and a specificity value ≥90%. If the sample size in the disease group was too small (<10), subgroup analysis was not performed.

For further details regarding the materials and methods used, please refer to the Supplementary CTAT table.

## Results

### Algorithm development study, STOP-HCC-ARP

#### Study population

Of the 1,097 patients enrolled, 1,006 had samples available for inclusion in the algorithm development study based on matched sample availability (Fig. 1B). The demographics and characteristics of the participants are shown (Table 1). Of all HCC patients, 123 (41.4%) had early- and 163 (54.9%) had late-stage HCC. In the early-stage HCC, late-stage HCC, and CLD control groups, respectively, mean age (standard deviation [SD]) was 60.5 (9.5), 60.6 (9.9), and 55.3 (10.7) years; 71.5%, 85.3%, and 56.3% were male; and 84.6%, 74.2%, and 53.2% had cirrhosis.

**Table 1:** Participant demographics and clinical characteristics in algorithm development study, STOP-HCC-ARP.

	Early-stage HCC (BCLC 0/A) (n = 123)	Late-stage HCC (BCLC B–D) (n = 163)	All-stage HCC (n = 297)∗	CLD controls (n = 709)
**Patient characteristics**				
Age, years, mean (SD)	60.5 (9.5)	60.6 (9.9)	60.8 (9.8)	55.3 (10.7)
Sex, n (%)†				
Male	88 (71.5)	139 (85.3)	233 (78.5)	399 (56.3)
Female	34 (27.6)	24 (14.7)	63 (21.2)	309 (43.6)

**Liver biochemistry, n (SD)**				
AST, U/L, median (IQR)‡	44.6 (28.0–78.5)	74.5 (48.6–123.9)	58.4 (39.2–104.8)	28.9 (22.5–45.3)
ALT, U/L, median (IQR)‡	26.8 (16.2–44.6)	34.0 (21.8–63.3)	30.5 (19.0–53.1)	20.0 (13.7–32.2)
Serum albumin, g/L, median (IQR)§	39.8 (34.0–44.6)	37.0 (32.0–41.8)	38.0 (33.0–43.0)	44.0 (41.0–46.0)
Serum total bilirubin, μmol/L, median (IQR)¶	16.8 (10.3–27.4)	17.1 (11.8–28.0)	16.9 (10.3–27.4)	12.0 (8.6–17.0)
ALBI score, median (IQR)	–2.5 (–3.0–1.9)	–2.3 (–2.8–1.8)	–2.4 (–2.9–1.9)	–3.0 (–3.3–2.7)

**Etiology, n (%)**∗∗				
Cirrhosis	104 (84.6)	121 (74.2)	232 (78.1)	377 (53.2)
Cirrhosis viral	97 (78.9)	102 (62.6)	205 (69.0)	350 (49.4)
Cirrhosis non-viral	28 (22.8)	34 (20.9)	64 (21.5)	59 (8.3)
Non-cirrhosis	15 (12.2)	29 (17.8)	47 (15.8)	330 (46.5)
Non-cirrhosis viral	16 (13.0)	28 (17.2)	47 (15.8)	339 (47.8)
Non-cirrhosis non-viral	3 (2.4)	8 (4.9)	11 (3.7)	67 (9.4)
Other	4 (3.3)	13 (8)	18 (6.1)	2 (0.3)

ALBI, albumin–bilirubin; ALT, alanine transaminase; ALT, alanine transaminase; AST, aspartate transferase; BCLC, Barcelona Clinic Liver Cancer; CLD, chronic liver disease; HCC, hepatocellular carcinoma; MELD, model for end-stage liver disease.

∗ HCC stage was unknown for 11 patients.

† Missing data for one patient with early-stage HCC, and two patients in the non-HCC CLD control groups.

‡ Missing data for five patients with HCC (two with early-stage; three with late-stage HCC), and 21 patients with non-HCC CLD.

§ Missing data for 19 patients with HCC (10 with early-stage; nine with late-stage HCC), and 122 patients with non-HCC CLD.

¶ Missing data for 20 patients with HCC (10 with early-stage; 10 with late-stage HCC), and 169 patients with non-HCC CLD.

∗∗ Patients could have multiple disease etiologies.

#### Clinical performance

The AUCs of GAAD (Cobas), GALAD (Cobas), and GALAD (μTASWAKO) were comparable and demonstrated good clinical performance in discriminating between early- (91.4%, 91.4%, and 90.8%, respectively), late- (98.3%, 98.4%, and 98.0%) and all-stage HCC (95.4%, 95.5%, and 95.0%) *vs.* CLD controls (Fig. 1C–E). The sensitivity and specificities of the algorithms are shown (Fig. 1F). When using a cut-off for GALAD (μTASWAKO) corresponding to 90% specificity for GAAD (Cobas), performance was comparable to both GAAD and GALAD (Cobas).

The GAAD and GALAD algorithms performed well, and AUCs remained comparable across different HCC etiologies (viral or non-viral disease etiologies with or without cirrhosis) (Fig. S1). In early-, late-, and all-stage HCC, respectively, AUCs for viral and non-viral etiologies were 88.2–97.6%, 95.7–99.5%, and 92.2–98.7%, and 90.0–97.1%, 98.5–100%, and 91.4–97.3%, respectively.

### Clinical validation study, STOP-HCC-MCE

#### Study population

**Fig. 2:**
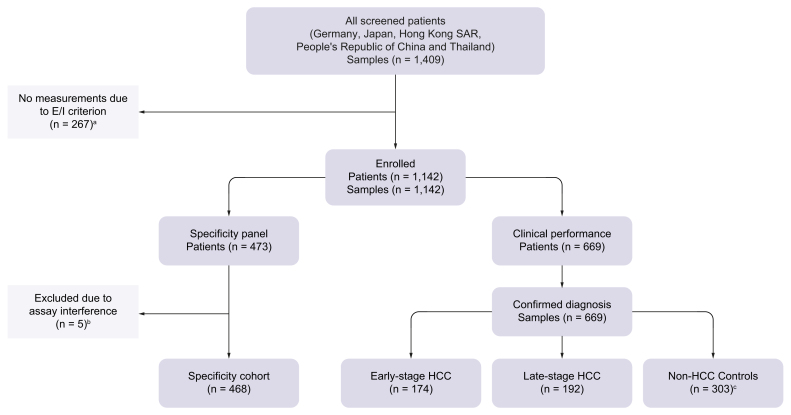
Study design and sample disposition in STOP-HCC-MCE. ^a^Excluded because of exclusion criteria (67 owing to renal failure, 17 owing to ICF issues, and 124 owing to lab parameters, sample processing, other cancer/missing diagnosis). ^b^Excluded because of interferences with assays. ^c^One non-HCC control subject was included for GAAD evaluation but excluded for GALAD evaluation due to bilirubin interference with AFP-L3 assay. AFP-L3, *Lens culinaris* agglutinin-reactive alpha-fetoprotein; E/I, exclusion/inclusion; GAAD, gender (biological sex), age, AFP, DCP (PIVKA-II); GALAD, gender (biological sex), age, AFP-L3, AFP, DCP (PIVKA-II); HCC, hepatocellular carcinoma; ICF, International Classification of Functioning, Disability and Health.

Patients enrolled and eligible for analysis in STOP-HCC-MCE (n = 1,142) were split into two cohorts; 668 evaluable participants (366 with HCC [174 early-stage; 192 late-stage] and 302 CLD controls) were included for analysis in the clinical performance cohort and 468 participants in the specificity panel cohort (Fig. 2).

Baseline characteristics of patients included in the clinical performance cohort are described (Table 2). In the early-stage HCC, late-stage HCC, and CLD control groups, respectively, mean age (SD) was 58.2 (11.4), 61.3 (11.2), and 49.6 (12.5) years; 83.9%, 84.4%, and 63.2% were male; 77.6%, 79.2%, and 37.1% had cirrhosis; and 88.5%, 66.7%, and 77.8% of patients had viral liver disease etiology (Table 2). Participant demographics and clinical characteristics for the specificity panel cohort, and the clinical performance cohort by clinical site, are shown (Tables S1 and S2, respectively).

**Table 2:** Participant demographics and clinical characteristics in clinical validation study, STOP-HCC-MCE.

	Early-stage (BCLC 0/A) HCC (n = 174)	Late-stage (BCLC B–D) HCC (n = 192)	Non-HCC CLD controls (n = 302)
**Patient characteristics**			
Age, years, mean (SD)	58.2 (11.4)	61.3 (11.2)	49.6 (12.5)
Sex, n (%)			
Male	146 (83.9)	162 (84.4)	191 (63.2)
Female	28 (16.1)	30 (15.6)	111 (36.8)
Race, n (%)			
Asian	135 (77.6)	91 (47.4)	181 (59.9)
White	39 (22.4)	99 (51.6)	112 (37.1)
Black/African American	0	1 (0.5)	3 (1.0)
Other/missing	0	1 (0.5)	6 (2.0)

**Liver disease etiology, n (%)**			
Viral liver disease etiology	154 (88.5)	128 (66.7)	235 (77.8)
Antiviral therapy in patients with viral liver disease etiology	74 (48.1)	63 (49.2)	121 (51.4)
HBV	127 (73.0)	105 (54.7)	174 (57.6)
HCV	27 (15.5)	23 (12.0)	61 (20.2)
MASH	9 (5.2)	21 (10.9)	62 (20.5)
ALD	14 (8)	42 (21.9)	23 (7.6)
Other	27 (15.5)	34 (17.7)	76 (25.2)
Cirrhosis	135 (77.6)	152 (79.2)	112 (37.1)
Cirrhotic HBV	97 (55.7)	81 (42.2)	60 (19.9)
Cirrhotic HCV	22 (12.6)	22 (11.5)	16 (5.3)
Cirrhotic MASH	7 (4.0)	14 (7.3)	7 (2.3)
Cirrhotic ALD	14 (8.0)	40 (20.8)	19 (6.3)
Cirrhotic other	23 (13.2)	28 (14.6)	29 (9.6)
Non-cirrhosis	39 (22.4)	40 (20.8)	190 (62.9)
Non-cirrhotic HBV	30 (17.2)	24 (12.5)	114 (37.7)
Non-cirrhotic HCV	5 (2.9)	1 (0.5)	45 (14.9)
Non-cirrhotic MASH	2 (1.1)	7 (3.6)	55 (18.2)
Non-cirrhotic ALD	0	2 (1.0)	4 (1.3)
Non-cirrhotic other	4 (2.3)	6 (3.1)	47 (15.6)

**Liver biochemistry and clinical features**			
AST, U/L, median (IQR)	34.0 (25.0–47.0)	66.5 (41.0–119.0)	29.0 (21.0-41.0)
ALT, U/L, median (IQR)	30.0 (23.0–42.0)	42.0 (30.0–68.0)	27.8 (20.0–44.8)
Serum albumin, g/L, median (IQR)	39.4 (36.0–41.8)	35.8 (31.8–40.0)	44.0 (40.0–47.0)
Serum total bilirubin, μmol/L, median (IQR)	14.1 (10.8–20.8)	15.4 (11.0–26.6)	11.0 (8.0–16.3)
PT-INR, n (%)			
1	174 (100.0)	187 (97.4)	—
2/3	0	5 (2.6)	—
Ascites, n (%)			
Mild	12 (6.9)	42 (21.9)	8 (2.7)
Moderate to severe	4 (2.3)	17 (8.9)	4 (1.3)
None	158 (90.8)	133 (69.3)	290 (96.0)
Hepatic encephalopathy, n (%)			
Grade I–II	2 (1.2)	4 (2.1)	4 (1.3)
None	172 (98.8)	188 (97.9)	298 (98.7)
MELD score, median (IQR)	8.0 (7.0–9.0)	8.0 (7.0–10.0)	7.0 (6.0–8.0)
ALBI score, median (IQR)	–2.6 (–2.8–2.3)	–2.3 (–2.6–1.8)	–3.1 (–3.3–2.7)
ALBI grade, n (%)			
1	85 (48.9)	55 (28.6)	241 (79.8)
2	85 (48.9)	114 (59.4)	56 (18.5)
3	4 (2.3)	23 (12)	5 (1.7)
Child–Pugh class, n (%)			
A	154 (88.5)	129 (67.2)	—
B	20 (11.5)	54 (28.1)	—
C	0 (0)	9 (4.7)	—
Tumor nodule number, n (%)			
1	147 (84.5)	45 (23.4)	NA
2	23 (13.2)	35 (18.2)	NA
3	4 (2.3)	17 (8.9)	NA
≥3	0 (0)	95 (49.5)	NA

**Tumor characteristics, n (%)**			
Large multinodular	0 (0)	87 (45.3)	NA
Portal invasion or extrahepatic spread (N1, M1)	0 (0)	82 (42.7)	NA
Single <2 cm	43 (24.7)	0 (0)	NA
Single or ≤3 nodules ≤3 cm	131 (75.3)	23 (12.0)	NA
**Size of index lesion, cm, mean (IQR; minimum, maximum)**	3.0 (1.8–3.8; 0–11.3)	7.6 (4.5–10.0; 0.7–18.4)	NA

ALBI, albumin–bilirubin; ALD, alcoholic-related liver disease; ALT, alanine transaminase; AST, aspartate transferase; BCLC, Barcelona Clinic Liver Cancer; CLD, chronic liver disease; HBV, hepatitis B virus; HCC, hepatocellular carcinoma; HCV, hepatitis C virus; MASH, metabolic dysfunction-associated steatohepatitis; MELD, model for end-stage liver disease; PT-INR, prothrombin time-international normalized ratio.

#### Method comparison and specificity panel analysis

**Fig. 3:**
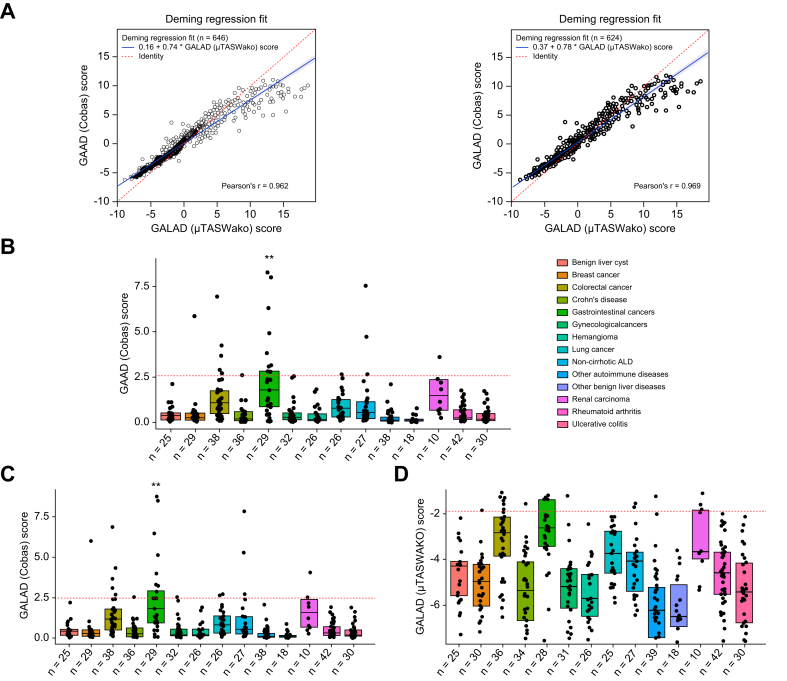
Weighted Deming regression fit and specificty panel data in STOP-HCC-MCE. Weighted Deming regression fit of agreement between GAAD (Cobas) and GALAD (μTASWAKO), GALAD (Cobas) and GALAD (μTASWAKO) in STOP-HCC-MCE (A), specificity panel data across disease groups for GAAD (Cobas) (B), GALAD (Cobas) (C), and GALAD (μTASWAKO) (D) in STOP-HCC-MCE. Levels of significance: ∗∗*p* <0.01. (*p* values were calculated using binomial tests, Bonferroni correction for every marker, and specificity values with the corresponding cut-offs for the markers: GAAD [2.57, 90%], GALAD [2.47, 90%], WAKO GALAD [−1.89, 90%].) ALD, alcoholic-related liver disease; GAAD, gender (biological sex), age, AFP, DCP (PIVKA-II); GALAD, gender (biological sex), age, AFP-L3, AFP, DCP (PIVKA-II).

A method comparison using weighted Deming regression analyses indicated an excellent analytical agreement between GALAD (μTASWAKO) and GAAD (Cobas) (Pearson’s r = 0.962; *p* <0.001), and GALAD (Cobas) (Pearson’s r = 0.969; *p* <0.001) algorithms in this cohort (Fig. 3A). In the specificity panel cohort (n = 468), low scores for GAAD (Cobas), GALAD (Cobas), and GALAD (μTASWAKO) were observed across disease groups, indicating high algorithmic panel specificity for HCC diagnosis except slight elevation in gastrointestinal cancers (median [IQR 1.79 [0.90–2.83], *p* <0.01; 1.82 [0.96–2.90], *p* <0.01); and -2.61 [-3.42 to -1.38] for GAAD (Cobas), GALAD (Cobas), and GALAD (μTASWAKO), respectively] (Fig. 3B–D).

#### Clinical performance

**Fig. 4:**
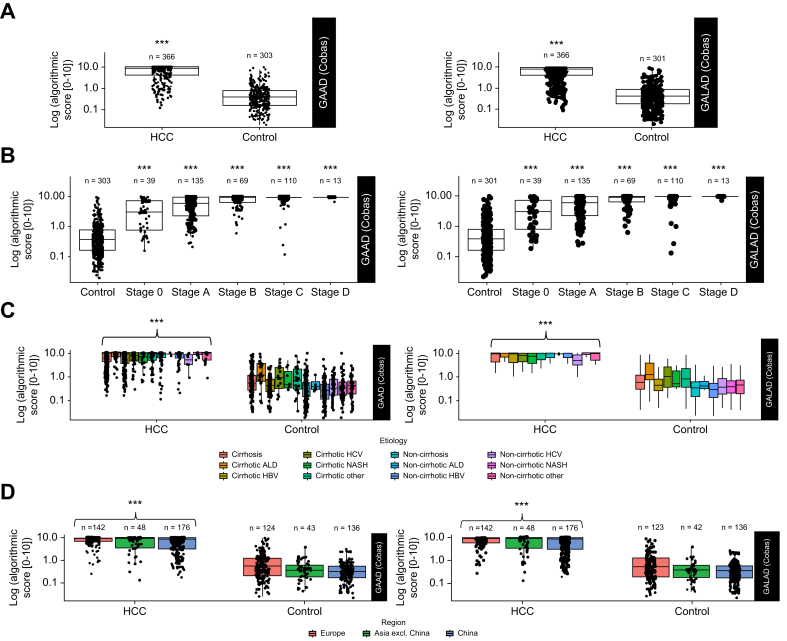
Distribution of GAAD (Cobas) and GALAD (Cobas) scores. Distribution by HCC and CLD controls (A), BCLC (B), etiology (C), and geographical region (D) in STOP-HCC-MCE. Levels of significance: ∗∗∗*p* <0.001 compared with control (Welch’s *t* test). ALD, alcoholic-related liver disease; BCLC, Barcelona Clinic Liver Cancer; CLD, chronic liver disease; CLD, chronic liver disease; GAAD, gender (biological sex), age, AFP, DCP (PIVKA-II); GALAD, gender (biological sex), age, AFP-L3, AFP, DCP (PIVKA-II); HBV, hepatitis B virus; HCC, hepatocellular carcinoma; HCV, hepatitis C virus; NASH, non-alcoholic steatohepatitis.

The distribution of GAAD and GALAD (Cobas) scores was both distinctly higher in all-stage HCC *vs.* CLD (median [IQR]: GAAD: 9.05 [4.49–9.96] *vs.* 0.39 [0.16–0.77], *p* <0.001; GALAD: 9.24 [4.78–9.98] *vs.* 0.41 [0.18–0.80], *p* <0.001) (Fig. 4A). For each BCLC stage, GAAD and GALAD (Cobas) scores were significantly higher for HCC patients compared with CLD controls (*p* <0.001) (Fig. 4B). Within the HCC case group, GAAD and GALAD scores remained high, irrespective of disease etiology (Fig. 4C) and geographic region (Fig. 4D); overall, scores for GAAD and GALAD for the HCC group were significantly higher compared with CLD controls (*p* <0.001).The detection of different HCC stages and CLD controls using single markers (Elecsys AFP, AFP-L3, and PIVKA-II assays) is shown (Fig. S2).

**Fig. 5:**
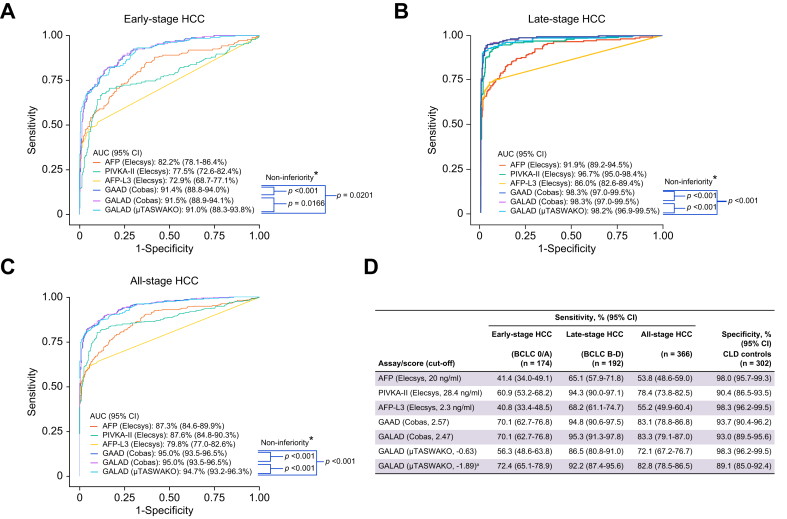
Clinical performance of individual Elecsys assays, AFP, PIVKA-II, and AFP-L3 and algorithmic scores, GAAD (Cobas), GALAD (Cobas), and GALAD (μTASWAKO) in STOP-HCC-MCE. Clinical performance in differentiating early-stage (A); late-stage (B); and all-stage HCC (C) from CLD controls. Levels of significance: early-stage HCC: GAAD (Cobas) *vs.* GALAD (Cobas) *p* <0.001; GAAD (Cobas) *vs.* GALAD (μTASWAKO) p = 0.0202; GALAD (Cobas) *vs.* GALAD (μTASWAKO) p = 0.0166; late- and all-stage HCC: *p* <0.001 (p values comparing non-inferiority of AUCs were calculated using the H0 hypothesis AUC (Test 1) < AUC (Test 2) - 0.01 with a studentized bootstrap approach using N = 1,000 bootstrap replicates.) Sensitivities and specificities are shown in table (D). ^a^Cut-off value corresponds to matching GAAD (Cobas) speciﬁcity of 90%. ∗AUC is not significantly worse by 1%. AFP, alpha-fetoprotein; AFP-L3, *Lens culinaris* agglutinin-reactive AFP; AUC, area under the curve; BCLC, Barcelona Clinic Liver Cancer; CLD, chronic liver disease; GAAD, gender (biological sex), age, AFP, DCP (PIVKA-II); GALAD, gender (biological sex), age, AFP-L3, AFP, DCP (PIVKA-II); HCC, hepatocellular carcinoma; PIVKA-II, protein induced by vitamin K absence or antagonist-II.

GAAD (Cobas), GALAD (Cobas), and GALAD (μTASWAKO) were able to discriminate between early- (AUC 91.0–91.5%), late- (98.2–98.3%), and all-stage HCC (94.7–95.0%) from CLD controls (Fig. 5A–C). The sensitivity for GAAD (Cobas) and GALAD (Cobas) was 70.1% for both in early-stage, 94.8% and 95.3% in late-stage, and 83.1% and 83.3% in all-stage HCC, respectively, at 93.7% (GAAD [Cobas]) and 93.0% (GALAD [Cobas]) specificity (Fig. 5D). When using two cut-offs for GALAD (μTASWAKO), respectively, (–0.63 and –1.89 [comparable to GAAD (Cobas) at 90% specificity]), the sensitivity was 56.3% and 72.4% in early-stage, 86.5% and 92.2% in late-stage, and 72.1% and 82.8% in all-stage HCC; specificity was 98.3% and 89.1% in CLD controls (Fig. 5D). GAAD (Cobas) was non-inferior (by a 1% margin) compared with GALAD (Cobas) and GALAD (μTASWAKO) across HCC stages (*p* <0.001), except for GALAD (μTASWAKO) in early-stage HCC (*p* = 0.02). A contingency table (Table S3) demonstrated that GAAD (Cobas) could detect more early-stage HCC cases, compared with GALAD (Cobas) and GALAD (μTASWAKO) (three and 17 cases, respectively).

The performance of GAAD (Cobas), GALAD (Cobas), and GALAD (μTASWAKO) was superior to individual biomarkers (AFP, AFP-L3, and PIVKA-II) alone across HCC stages (Fig. 5A–D). For individual biomarkers and different algorithms, clinical performance at predefined cut-offs are presented in Table S4, and cut-offs at different specified sensitivity and specificity in Tables S5 and S6, respectively.

**Fig. 6:**
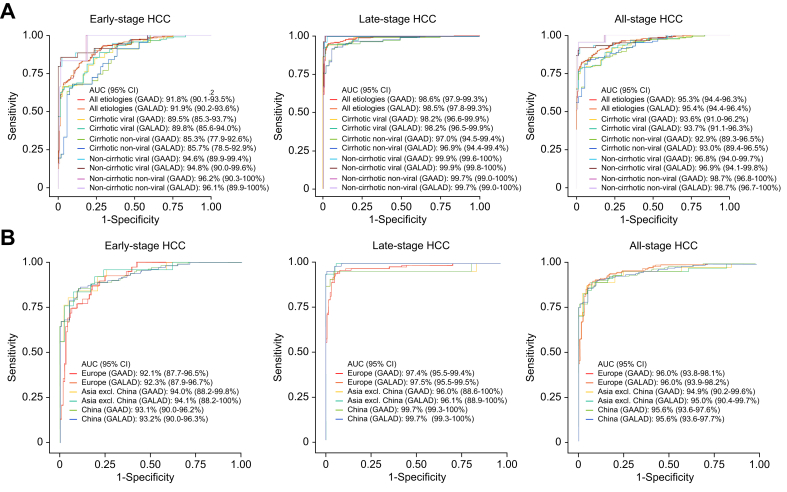
ROC curves of GAAD (Cobas), GALAD (Cobas) scores for discriminating between HCC and CLD controls in STOP-HCC-MCE. Overall and by etiology group in early-stage, late-stage and all-stage HCC (A); and by geographical region in early-stage, late-stage and all-stage HCC (B). AUC, area under the curve; CLD, chronic liver disease; GAAD, gender (biological sex), age, AFP, DCP (PIVKA-II); GALAD, gender (biological sex), age, AFP-L3, AFP, DCP (PIVKA-II); HCC, hepatocellular carcinoma.

In early-, late-, and all-stage HCC with cirrhotic etiology, AUCs were comparable at 85.3–89.5%, 97.0–98.2%, and 92.9–93.6% for GAAD (Cobas), and 85.7–89.8%, 96.9–98.2%, and 93.0–93.7% for GALAD (Cobas), respectively (Fig. 6A). AUCs for GAAD and GALAD (Cobas) were also similarly high (94.6–99.9%) across all HCC disease stages in subgroups without cirrhosis (Fig. 6A). When samples were split by region (i.e. Europe, Asia Pacific, China), AUCs for GAAD (Cobas) and GALAD (Cobas) remained similar, regardless of disease stage, ranging from 92.1% to 94.1% for early-, from 96.0% to 99.7% for late-, and from 94.9% to 96.0% for all-stage HCC (Fig. 6B).

## Discussion

Our study demonstrated for the first time that the GAAD (Cobas), GALAD (Cobas), and GALAD (μTASWAKO) algorithms performed equivalently well in differentiating HCC cases from CLD controls, irrespective of disease stage, etiology, or region. These findings suggest that GAAD may be a useful tool in identifying patients across etiologies and geographical regions who may benefit from more rigorous screening or liver transplantation. In the clinical validation study (STOP-HCC-MCE), GAAD (Cobas; cut-off 2.57), GALAD (Cobas; cut-off 2.47), and GALAD (μTASWAKO; cut-off –1.89) were similar in their ability to differentiate early- (AUC: 91.0–91.5%), and all-stage HCC (94.7–95.0%) from benign CLD. Similar to GALAD (Cobas), GAAD (Cobas) also achieved high specificity of 93.7%, and low false positive rates and high true negative detection rates, regardless of HCC stage. Performance data of the GALAD algorithms in our study were reflective of a previously published large meta-analysis including over 19,000 patients; for example the sensitivity, specificity, and AUC of GALAD were 73% (95% CI: 66–79%), 87% (95% CI: 81–91%), and 86% (95% CI: 82–88%), respectively, for the diagnosis of early-stage HCC.^12^

When considering the similar performance of GAAD and GALAD in our study with changing disease etiologies, treatment paradigms, and other phase II biomarker studies, questions arise regarding the utility of AFP-L3 in HCC surveillance. Although AFP-L3 alone is effective in detecting early-stage HCC with AFP+ tumors, with 9–12 months of lead time, compared with imaging techniques,^26^ AFP-L3 is usually not detected when AFP levels are <20 ng/ml. This was reflected in STOP-HCC-MCE; of 926 patients with AFP <20 ng/ml, only 10.9% of patients had detectable AFP-L3 (data not shown). Therefore, AFP-L3 may not be relevant for HCC diagnosis in individuals with AFP-negative tumors.^27^ Although global viral hepatitis-related HCC incidence has plateaued, HBV remains the most common cause of HCC in Asia,^28^^,^^29^ as reflected in our clinical validation study (79.4% at Asian sites had HBV, and around 50% of these patients were treated with antiviral therapies, likely leading to normalized AFP levels).^20^ Lower AFP levels at HCC diagnosis have also been reported across other etiologies, including MASLD,^30^^,^^31^ in which PIVKA-II demonstrated better HCC diagnostic ability.^31^ As non-viral etiologies are increasing worldwide, it will be beneficial to combine biomarkers, along with demographic characteristics, to improve diagnostic performance, as observed here with the GAAD algorithm—potentially useful for surveillance programs in all at-risk etiologies.

Additionally, biomarker combinations of PIVKA-II and AFP showed significantly higher clinical performance than AFP, PIVKA-II and AFP-L3 combined (AUCs 0.753 and 0.690, respectively; *p* = 0.001),^32^ further signifying that AFP-L3 may have a negligible role in HCC detection. Similarly, the prospective ESCALON study revealed that AFP-L3 contributed minimally to early-stage HCC detection in two large multicenter cohorts from Latin America and Europe.^33^ The value of AFP-L3 in algorithms that combine demographic characteristics and serum biomarkers is unclear. Previously, a phase III biomarker study found that, for HCC detection, the GALAD algorithm showed an improvement in sensitivity but with a similar AUC compared with AFP-L3 alone, suggesting that AFP-L3 contributed to GALAD performance.^16^ Whereas, in our phase II studies, both the GAAD and GALAD (Cobas) algorithms were more sensitive than AFP-L3 in detecting early-stage HCC (70.1% [95% CI: 62.7–76.8%] for both algorithms and 40.8% [95% CI: 33.4–48.5%] for AFP-L3), had higher AUCs (91.4% *vs.* 91.5% *vs.* 72.9%) for detecting early-stage HCC and had higher overall true positive rates (25.6% *vs.* 25.7% *vs.* 14.9%). Although GALAD (Cobas) showed slightly higher false positivity rates (GAAD: 6.27% *vs.* GALAD: 6.98%), GAAD and GALAD (Cobas) demonstrated similar clinical performance in our study overall, suggesting that AFP-L3 has little influence on clinical performance.

The GAAD algorithm may also reduce costs and resource burden on the healthcare system. For example, in China, the cost of an AFP-L3 test was ∼27.7 USD compared with ∼6.2 USD for an AFP test, and ∼18.5 USD for a PIVKA-II test; in a simulated cohort of 5,000 patients with HBV, at a cost-effectiveness threshold of three times China’s GDP per capita, GAAD + USG was the most cost-effective screening strategy for HCC compared with USG and biomarkers alone.^17^^,^^21^^,^^34^ Additionally, in separate simulated cohorts of 100,000 patients in the UK, cost-utility analysis found that GAAD was a cost-effective surveillance strategy compared with USG alone and USG + AFP.^35^

In the specificity panel analysis, of the conditions assessed, relatively high single biomarker concentrations (validated with μTASWAKO assays), and GAAD and GALAD scores, were observed for patients with gastrointestinal cancer. Patients with cholangiocarcinoma and pancreatic cancer were excluded from the analysis owing to high PIVKA-II expression levels. The small sample size was a limitation; however, previous studies also reported high PIVKA-II expression levels in patients with certain gastrointestinal, cholangiocarcinoma, and pancreatic cancers potentially attributable to underlying conditions (e.g. cholestatic disease, cholangitis, biliary stenosis, or bile duct stones) or the administration of antibiotics containing N-methyl-thiotetrazole during treatment.^36^^,^^37^ These findings indicate that high GAAD and GALAD scores should be used in conjunction with other clinical data, such as imaging, patient symptoms, and other laboratory tests.

Given the global plateau in viral hepatitis-related HCC incidence, our study was limited by the low number of patients with non-viral HCC etiology. This was because of the high proportion of Asian patients in our study, where viral etiologies are prevalent, therefore, additional analyses in larger non-viral cohorts may be beneficial. Another limitation was that patient evaluations were conducted in specialized care centers. Further studies should evaluate the clinical performance of GAAD and GALAD in primary care and non-specialist settings where serum assays can also be implemented for a better understanding of real-world performance.

Ultimately, the aim of this study was to validate and provide evidence to support the introduction of the GAAD algorithm as a potential alternative method to GALAD for the detection of HCC, which may result in optimized healthcare spend and improved patient outcomes. In summary, GAAD exhibits high sensitivity and low false positivity for the detection of early-stage HCC, performing favorably compared with individual surveillance biomarkers across disease etiologies and geographical regions. However, further validation through larger phase III/IV studies is required to assess the benefit-to-harm ratio of GAAD-based surveillance. Nonetheless, these findings demonstrate the potential of blood-based biomarker panels in addressing the significant unmet need for early detection of HCC.

### Conclusions

The GAAD (Cobas) algorithm demonstrated good clinical performance and was as sensitive and specific as the GALAD (Cobas) and GALAD (μTASWAKO) algorithms in differentiating HCC and CLD controls, across all disease stages, etiologies, and regions. In participants with CLD undergoing guideline-directed HCC surveillance, the GAAD (Cobas) algorithm may be a time- and cost-efficient tool for early-stage HCC detection, with the potential to increase curative treatment opportunities and reduce mortality.

## Abbreviations

AFP, alpha-fetoprotein; AFP-L3, *Lens culinaris* agglutinin-reactive AFP; ALBI, albumin–bilirubin; ALD, alcoholic-related liver disease; ALT, alanine transaminase; AST, aspartate transferase; AUC, area under the curve; BCLC, Barcelona Clinic Liver Cancer; CLD, chronic liver disease; DCP, des-gamma carboxyprothrombin (PIVKA-II); FN, false negatives; FP, false positives; GAAD, gender (biological sex), age, AFP, DCP (PIVKA-II); GALAD, gender (biological sex), age, AFP-L3, AFP, DCP (PIVKA-II); HBV, hepatitis B virus; HCC, hepatocellular carcinoma; HCV, hepatitis C virus; ICF, International Classification of Functioning, Disability and Health; IQR, interquartile range; MASH, metabolic dysfunction-associated steatohepatitis; MASLD, metabolic dysfunction-associated steatotic liver disease; MELD, model for end-stage liver disease; NE, not evaluable; NPV, negative predictive value; PIVKA-II, protein induced by vitamin K absence or antagonist-II; PPV, positive predictive value; PS, performance status; PT-INR, prothrombin time-international normalized ratio; ROC, receiver operating characteristics; SAR, Special Administrative Region; TN, true negatives; TP, true positives; USG, ultrasonography.

## Writing assistance

Editorial support for this manuscript, under the direction of the authors, was provided by Carolyn Bowler, PhD, CMPP™, Estelle Challinor, MSc, BSc, and Jade Drummond, BSc, of inScience Communications, Springer Healthcare Ltd, UK, and was funded by Roche Diagnostics International AG (Rotkreuz, Switzerland).

## Declarations

GAAD is a CE-marked digital tool for aid in diagnosis of early-stage and all-stage HCC. ELECSYS and COBAS are trademarks of Roche. All other product names and trademarks are the property of their respective owners.

## Financial support

This study was funded by Roche Diagnostics GmbH (Penzberg, Germany).

## Authors’ contributions

Conceptualization: AS, HLYC, TB. Funding acquisition: AS. Investigation: AS, AV, ENDeT, JH, JKH, JT, KMal, KMad, MK, TB, TP, WS. Methodology: AS, HLYC, KK, KMal, KMad, MK, PF. Supervision: AS, ENDeT, HLYC, JH, PF, TB, TP. Validation: AS, HLYC, KK, KMal, KMad, MK. Visualization: AS, KK. Formal analysis: HLYC, KMal, KMad, TB. Project administration: JKH. Data curation: AV, JT, KK, KMal, KMad, MK, PF. Software: KK. Resources: PF. Contributed to the writing and reviewing of the manuscript, and approval of the final manuscript: all authors.

## Data availability statement

Requests concerning the data supporting the findings of this study can be directed to rotkreuz.datasharingrequests@roche.com for consideration.

## Conflicts of interest

JH reports speaker’s bureau participation for Glaxo-Smith-Kline, Gilead Sciences, Roche Diagnostics, grant/research support from Gilead Sciences, BMS, advisory committee or review panel for Aligos, Assembly, Glaxo-Smith-Kline, Gilead Sciences, Johnson Pharmaceutica, and Roche. TB reports consultancy fees from Bayer, Eisai, Ipsen, Merck Sharp & Dome/Merck, Sirtex, and Roche. AV reports consultancy fees from AstraZeneca, Amgen, BeiGene, Böhringer Mannheim, BMS, BTG, Daichi-Sankyo, EISAI, Incyte, Ipsen, MSD, PierreFabre, Roche, Servier, Sirtex, Tahio, Terumo. Speaker for AstraZeneca, Amgen, BeiGene, Böhringer Mannheim, BMS, BTG, Daichi-Sankyo, EISAI, GSK, Imaging Equipment Ltd (AAA), Incyte, Ipsen, Jiangsu Hengrui Medicines MSD, PierreFabre, Roche, Servier, Sirtex, Tahio, Terumo. Research funding from Servier, and Incyte. Commercial medical education provider for Onclive, Oncowissen.de. TP reports speaker’s bureau participation for Bristol-Myers Squibb, Gilead Science, Bayer, Abbott, and Eisai, and MSD and research grant/contracts from Gilead Science, Roche Diagnostics, Jannsen Fibrogen, and VIR. JT reports consultancy fees for Amgen, Bayer Healthcare, Bristol-Myers Squibb, Eisai, Ipsen, Merck Serono, Merck Sharp & Dome, Lilly ImClone, and Roche. ENDeT has served as a paid consultant for AstraZeneca, Bayer, BMS, EISAI, Eli Lilly & Co, MSD, Mallinckrodt, Omega, Pfizer, Ipsen, Terumo and Roche and is currently employed by Boehringer-Ingelheim. He has received reimbursement of meeting attendance fees and travel expenses from Arqule, AstraZeneca, BMS, Bayer, Celsion, and Roche, and lecture honoraria from BMS and Falk. He has received third-party funding for scientific research from Arqule, AstraZeneca, BMS, Bayer, Eli Lilly, Ipsen, and Roche. MK reports speaking and teaching for Eisai, Bayer, Merck Sharp & Dome, Bristol-Myers Squibb, Eli Lilly & Co, and EA Pharma, and grant/research support from Gilead Sciences, Taiho, Sumitomo Dainippon Pharma, Takeda, Otsuka, EA Pharma, AbbVie, Eisai, Ono, and advisory committee or review panel for Eisai, Ono, MSD, Bristol-Myers Squibb, and Roche. KMal is an employee of Microcoat Biotechnologie, contracted by Roche Diagnostics. KMad, KK, and AS are employees of Roche Diagnostics International AG. PF, JKH, and WS have no conflicts to declare. HLYC reports consultancy fees from Arbutus Biopharma, Gilead Sciences, Glaxo-Smith-Kline, Roche, Vir Biotechnology, Aligos Therapeutics, Vaccitech, and Virion Therapeutics, and speaker’s bureau participation for Echosens, Gilead Sciences, Roche, and Viatris.

Please refer to the accompanying ICMJE disclosure forms for further details.
