# Supplementary material for: Comparative evaluation of multimarker algorithms for early-stage HCC detection in multicenter prospective studies
Source: JHEP Rep. 2024 Nov 8;7(2):101263. doi: 10.1016/j.jhepr.2024.101263 (PMC11782856; doi:10.1016/j.jhepr.2024.101263)
Supplement: Multimedia component 1 [file mmc1.pdf]

Comparative evaluation of multimarker algorithms for early-stage HCC detection in multicenter prospective studies

Jinlin Hou, Thomas Berg, Arndt Vogel, Teerha Piratvisuth, Jörg Trojan, Enrico N. De Toni, Masatoshi Kudo, Katarina Malinowsky, Peter Findeisen, Johannes Kolja Hegel, Wenzel Schöning, Kairat Madin, Konstantin Kroeniger, Henry Lik-Yuen Chan, Ashish Sharma

Table of contents

Fig. S1 2

Fig. S2 5

Table S1 6

Table S2 8

Table S3 12

Table S4 14

Table S5 17

Table S6 21

Fig. S1. Clinical performance of GAAD (cobas), GALAD (cobas), and GALAD (μTASWAKO) algorithmic scores in STOP-HCC-ARP for differentiating between early-stage HCC (A), late-stage HCC (B), and all-stage (C) and disease controls by etiology.

A.

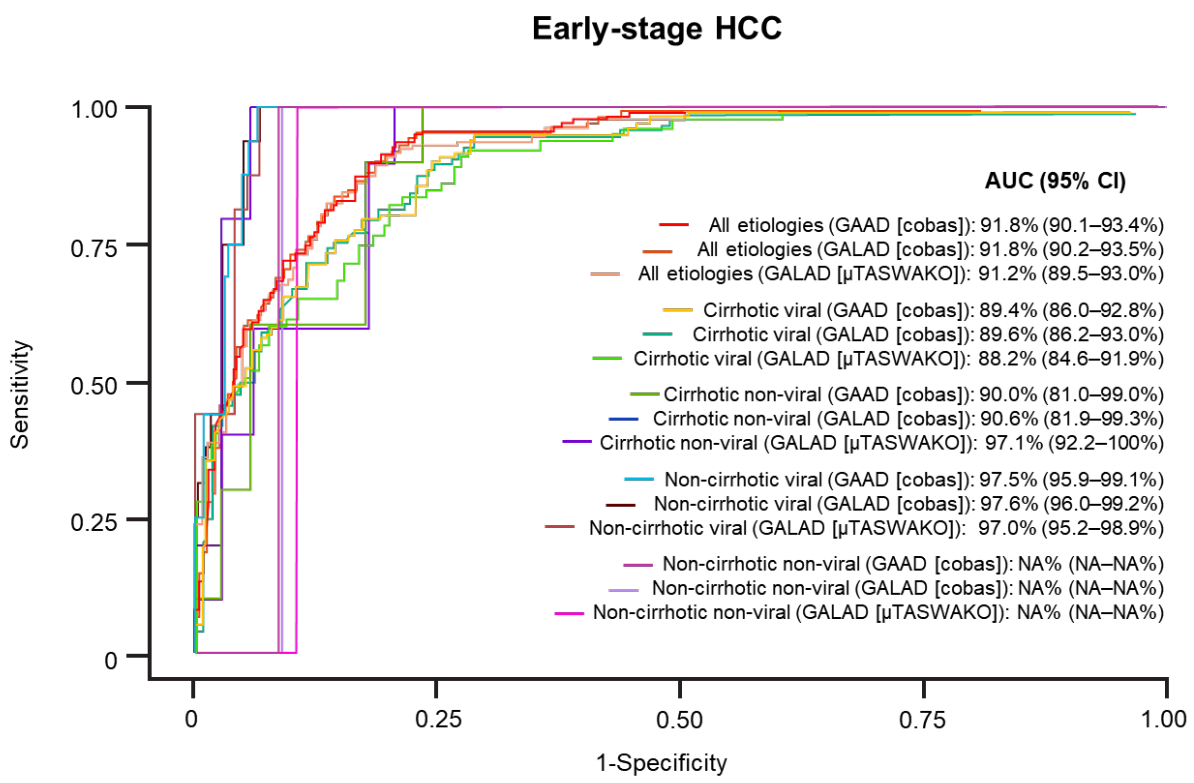

B.

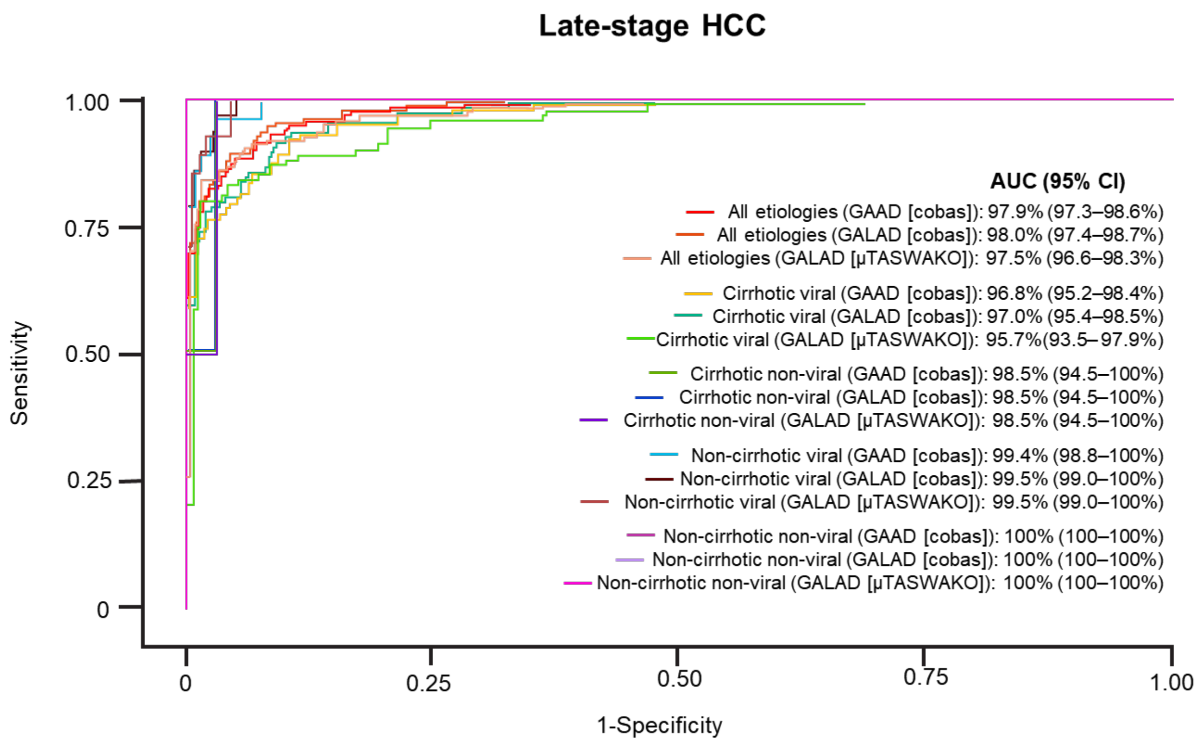

Late-stage HCC
Sensitivity
1-Specificity
1.00
0.75
0.50
0.25
0
0
0.25
0.50
0.75
1.00
AUC (95% CI)
All etiologies (GAAD [cobas]): 97.9% (97.3–98.6%)
All etiologies (GALAD [cobas]): 98.0% (97.4–98.7%)
All etiologies (GALAD [μTASWAKO]): 97.5% (96.6–98.3%)
Cirrhotic viral (GAAD [cobas]): 96.8% (95.2–98.4%)
Cirrhotic viral (GALAD [cobas]): 97.0% (95.4–98.5%)
Cirrhotic viral (GALAD [μTASWAKO]): 95.7%(93.5–97.9%)
Cirrhotic non-viral (GAAD [cobas]): 98.5% (94.5–100%)
Cirrhotic non-viral (GALAD [cobas]): 98.5% (94.5–100%)
Cirrhotic non-viral (GALAD [μTASWAKO]): 98.5% (94.5–100%)
Non-cirrhotic viral (GAAD [cobas]): 99.4% (98.8–100%)
Non-cirrhotic viral (GALAD [cobas]): 99.5% (99.0–100%)
Non-cirrhotic viral (GALAD [μTASWAKO]): 99.5% (99.0–100%)
Non-cirrhotic non-viral (GAAD [cobas]): 100% (100–100%)
Non-cirrhotic non-viral (GALAD [cobas]): 100% (100–100%)
Non-cirrhotic non-viral (GALAD [μTASWAKO]): 100% (100–100%)

C.

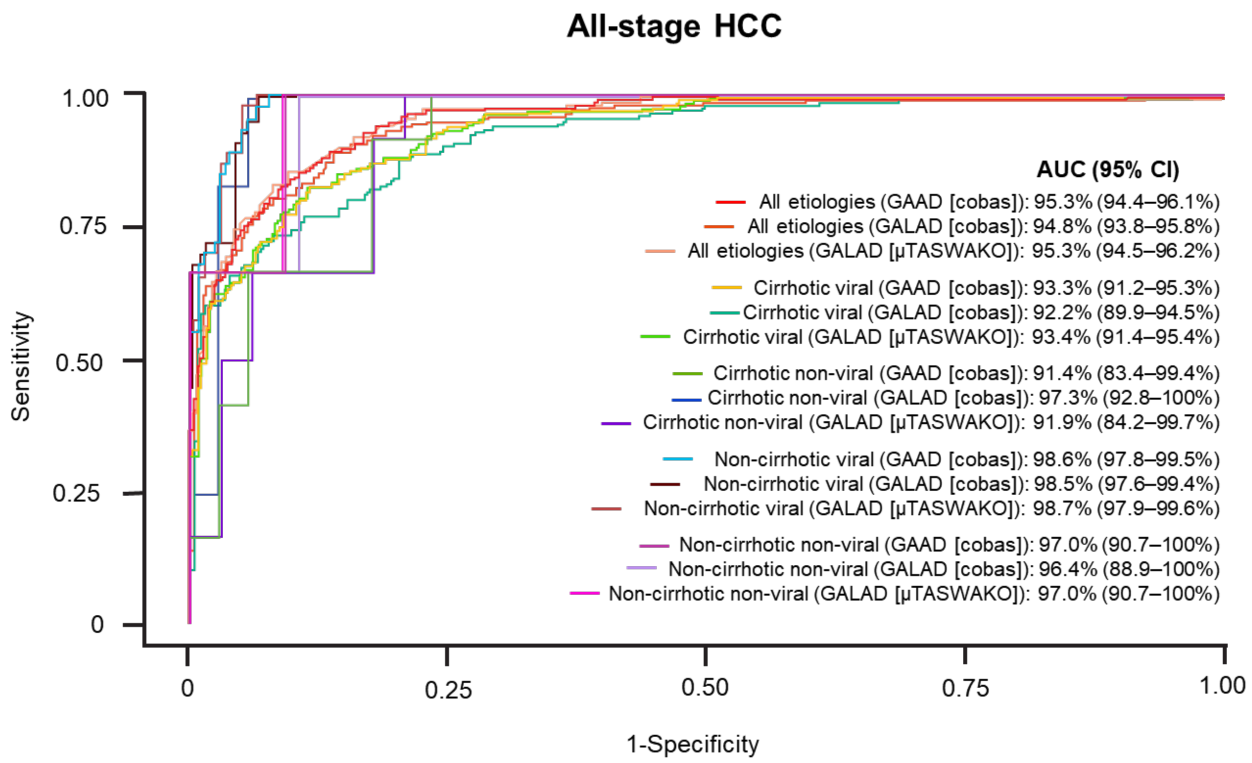

HCC, hepatocellular carcinoma.

Fig. S2. Venn diagram to illustrate the number of early-, late-, and, all-stage HCC cases, or controls detected by the single markers (Elecsys AFP, Elecsys AFP-L3, Elecsys PIVKA-II) using the predefined cutoffs (AFP 20 ng/mL; AFP-L3 2.3 ng/mL; PIVKA-II 28.4 ng/mL) in STOP-HCC-MCE.

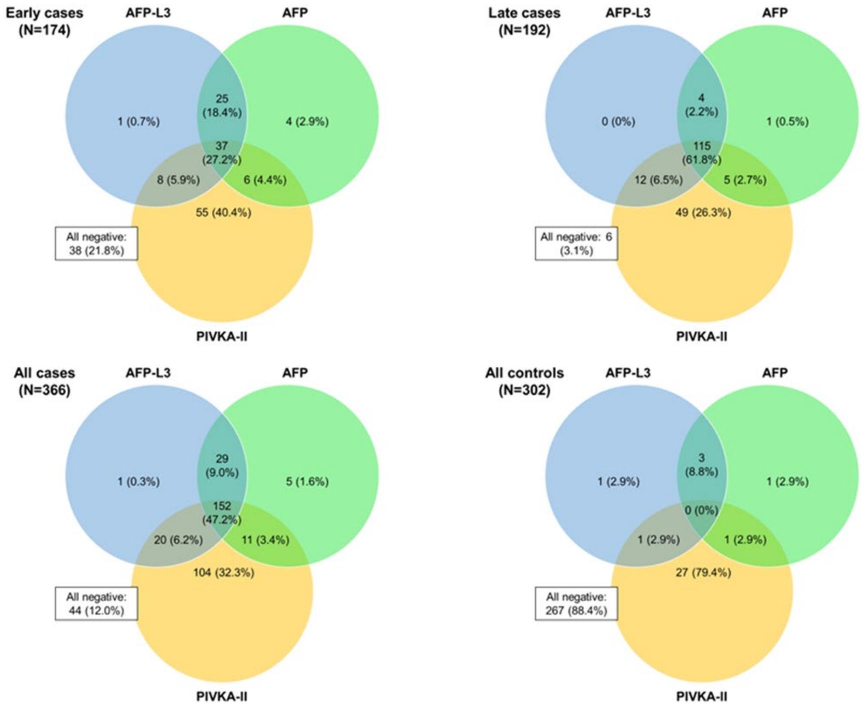

HCC, hepatocellular carcinoma.

Table S1. Participant demographics and clinical characteristics of the specificity panel in STOP-HCC-MCE.

	**Total (N=468)**
**Patient characteristics**	
**Age, years, mean (SD)**	53.9 (14.5)
**Sex, n (%)***	
Male	186 (39.7)
Female	282 (60.3)
**Race, n (%)***	
Asian	178 (38.0)
White	289 (61.8)

Black/African-American	1 (0.2)
Other	0
**Ongoing antiviral therapy, n (%)**	3 (0.6)
**Antibiotics, n (%)**	22 (4.7)

SD, standard deviation.

Table S2. Participant demographics and clinical characteristics in STOP-HCC-MCE by clinical sites.

	Hanover (N=88)	Leipzig (N=93)	Hat Yai (N=75)	Hong Kong* (N=109)	Guangzhou (N=203)	Frankfurt (N=46)	Berlin (N=1)	Munich (N=29)	Osaka (N=16)	Mainz (N=8)	Total (N=668)
**Patient characteristics**											
**Age, mean (SD)**	55.7 (14.8)	59.9 (12.2)	54.0 (10.4)	58.3 (10.3)	47.9 (11.0)	65.2 (12.0)	64.0 (NE)	58.2 (10.8)	67.9 (12.7)	51.6 (13.7)	55.2 (12.9)
**Sex, n (%)**											
Male	58 (65.9)	65 (69.9)	53 (70.7)	78 (71.6)	167 (82.3)	39 (84.8)	1 (100.0)	26 (89.7)	8 (50.0)	4 (50.0)	499 (74.7)
Female	30 (34.1)	28 (30.1)	22 (29.3)	31 (28.4)	36 (17.7)	7 (15.2)	0	3 (10.3)	8 (50.0)	4 (50.0)	169 (25.3)
**Race, n (%)**											
Asian	4 (4.6)	0	75 (100.0)	109 (100.0)	202 (99.5)	1 (2.2)	0	0	16 (100.0)	0	407 (60.9)
White	76 (86.4)	93 (100.0)	0	0	0	44 (95.7)	1 (100.0)	28 (96.6)	0	8 (100.0)	250 (37.4)

Black/ African American	2 (2.3)	0	0	0	0	1 (2.2)	0	1 (3.5)	0	0	4 (0.6)
Other	0	0	0	0	1 (0.5)	0	0	0	0	0	1 (0.2)
Missing	6 (6.8)	0	0	0	0	0	0	0	0	0	6 (0.9)
**Ongoing antiviral therapy, n (%)**	24 (27.3)	2 (2.2)	27 (36.0)	62 (56.9)	132 (65.0)	3 (6.5)	1 (100.0)	4 (13.8)	4 (25.0)	1 (12.5)	260 (38.9)
**Group, n (%)**											
HCC cases	39 (44.3)	41 (44.1)	40 (53.3)	56 (51.4)	120 (59.1)	38 (82.6)	1 (100.0)	21 (72.4)	8 (50.0)	2 (25.0)	366 (54.8)
Early-stage (BCLC 0/A)	7 (8.0)	9 (9.7)	19 (25.3)	43 (39.4)	67 (33.0)	8 (17.4)	1 (100.0)	12 (41.4)	6 (37.5)	2 (25.0)	174 (26.0)

Late-stage (BCLC B–D)	32 (36.4)	32 (34.4)	21 (28.0)	13 (11.9)	53 (26.1)	30 (65.2)	0	9 (31.0)	2 (12.5)	0	192 (28.7)
CLD controls	49 (55.7)	52 (55.9)	35 (46.7)	53 (48.6)	83 (40.9)	8 (17.4)	0	8 (27.6)	8 (50.0)	6 (75.0)	302 (45.2)
**Viral liver disease etiology, n (%)**	69 (78.4)	26 (28.0)	78 (104.0)	95 (87.2)	196 (96.6)	26 (55.3)	1 (100)	8 (27.6)	13 (81.3)	6 (75.0)	518 (669)
**Liver biochemistry & prognostic scores**											
AST, U/L, median (IQR)	36.5 (27.0–59.8)	38.6 (31.9–63.9)	44.0 (26.0–74.0)	33.0 (24.0–49.0)	28.0 (21.0–47.0)	46.5 (38.3–66.8)	37.0 (37.0–37.0)	60.0 (42.0–74.0)	30.5 (22.8–34.0)	31.0 (27.8–52.5)	36.0 (25.0–59.0)
ALT, U/L, median (IQR)	33.5 (25.0–48.3)	32.4 (23.4–46.2)	35.0 (23.5–56.0)	37.0 (23.0–53.0)	29.0 (20.0–43.0)	42.5 (31.3–58.5)	52.0 (52.0–52.0)	35.0 (27.0–48.0)	17.5 (13.0–22.5)	36.0 (21.3–47.0)	32.4 (22.0–49.1)

Serum albumin, g/L, median (IQR)	39.0 (35.0–43.0)	42.7 (38.1–47.2)	42.0 (35.5–45.5)	39.0 (36.0–40.0)	41.7 (36.7–45.7)	40.0 (37.0–44.0)	48.4 (48.4–48.4)	33.0 (30.0–40.0)	43.5 (41.0–44.3)	39.5 (37.5–40.3)	40.0 (36.0–44.3)
Serum bilirubin, μmol/L, median (IQR)	10.0 (6.0–15.0)	14.0 (8.8–23.6)	14.7 (8.7–23.0)	12.0 (9.0–18.0)	13.7 (10.6–19.3)	12.0 (10.3–15.4)	9.1 (9.1–9.1)	23.9 (15.4–46.2)	12.8 (10.3–17.1)	12.0 (9.8–12.8)	13.0 (9.2–20.0)

*Special Administrative Region of China.

Percentages may not add up to 100.0% due to rounding.

AST; aspartate aminotransferase; ALT; alanine aminotransferase; BCLC, Barcelona Clinic Liver Cancer; CLD, chronic liver disease; HCC, hepatocellular carcinoma; IQR, interquartile range; SD, standard deviation.

Table S3. Contingency tables for GAAD (cobas) compared with GALAD (cobas) and GALAD (μTASWAKO) in STOP-HCC-MCE.

HCC stage		GAAD (cobas)+	GAAD (cobas)-	Total
Early-stage	GALAD (cobas)+	111	0	111
	GALAD (cobas)-	3	51	54
	**Total**	114	51	165
All-stage	GALAD (cobas)+	272	1	273
	GALAD (cobas)-	3	60	63
	**Total**	275	61	336
Early-stage	GALAD (μTASWAKO)+	102	0	102
	GALAD (μTASWAKO)-	17	50	67
	**Total**	119	50	169
All-stage	GALAD (μTASWAKO)+	264	0	264
	GALAD (μTASWAKO)-	23	60	83
	**Total**	287	60	347

HCC, hepatocellular carcinoma.

GAAD+ (cobas) corresponds to patients with a GAAD (cobas) score ≥2.57, GAAD- (cobas) corresponds to patients with a GAAD (cobas) score <2.57.

GALAD+ (cobas) corresponds to patients with a GALAD (cobas) score ≥2.47, GALAD- (cobas) corresponds to patients with a GALAD (cobas) score <2.47.

GALAD+ (μTASWAKO) corresponds to patients with a GALAD (μTASWAKO) score ≥2.95, GALAD- (μTASWAKO) corresponds to patients with a GALAD (μTASWAKO) score <2.95.

Table S4. Clinical performance of Elecsys assays, AFP, PIVKA-II and AFP-L3 and algorithmic scores GAAD (cobas), GALAD (cobas), and GALAD (μTASWAKO) for the detection of early-stage (BCLC 0/A), late-stage (BCLC B–D), and all-stage HCC at the predefined cut-offs in STOP-HCC-MCE.

	**AFP**			**PIVKA-II**			**AFP-L3**			**GAAD**			**GALAD**			**GALAD (μTASWAKO)**		
	**Early stage**	**Late stage**	**All stage**	**Early stage**	**Late stage**	**All stage**	**Early stage**	**Late stage**	**All stage**	**Early stage**	**Late stage**	**All stage**	**Early stage**	**Late stage**	**All stage**	**Early stage**	**Late stage**	**All stage**
N (HCC/CLD control)	477 (174/ 303)	495 (192/ 303)	669 (366/ 303)	477 (174/ 303)	495 (192/ 303)	669 (366/ 303)	475 (174/ 301)	493 (192/ 301)	667 (366/ 301)	477 (174/ 303)	495 (192/ 303)	669 (366/ 303)	475 (174/ 301)	493 (192/ 301)	667 (366/ 301)	472 (173/ 299)	485 (186/ 299)	685 (359/ 299)
TP	72	125	197	106	181	287	71	131	202	122	182	304	122	183	305	99	168	267
TN	297	297	297	274	274	274	296	296	296	284	284	284	280	280	280	297	297	297
FP	6	6	6	29	29	29	5	5	5	19	19	19	21	21	21	2	2	2
FN	102	67	169	68	11	79	103	61	164	52	10	62	52	9	61	74	18	92

**Sensitivity (95% CI)**	**41.4 (34.0–49.1)**	**65.1 (57.9–71.8)**	**53.8 (48.6–59.0)**	**60.9 (53.2–68.2)**	**94.3 (90.0–97.1)**	**78.4 (73.8–82.5)**	**40.8 (33.4–48.5)**	**68.2 (61.1–74.7)**	**55.2 (49.9–60.4)**	**70.1 (62.7–76.8)**	**94.8 (90.6–97.5)**	**83.1 (78.8–86.8)**	**70.1 (62.7–76.8)**	**95.3 (91.3–97.8)**	**83.3 (79.1–87.0)**	**57.2 (49.5–64.7)**	**90.3 (85.1–94.2)**	**74.4 (69.5–78.8)**
**Specificity (95% CI)**	**98 (95.7–99.3)**	**98 (95.7–99.3)**	**98 (95.7–99.3)**	**90.4 (86.5–93.5)**	**90.4 (86.5–93.5)**	**90.4 (86.5–93.5)**	**98.3 (96.2–99.5)**	**98.3 (96.2–99.5)**	**98.3 (96.2–99.5)**	**93.7 (90.4–96.2)**	**93.7 (90.4–96.2)**	**93.7 (90.4–96.2)**	**93 (89.5–95.6)**	**93 (89.5–95.6)**	**93 (89.5–95.6)**	**99.3 (97.6–99.9)**	**99.3 (97.6–99.9)**	**99.3 (97.6–99.9)**
PPV 1% prev	17.4	24.9	21.5	6.0	9.1	7.6	19.9	29.3	25.1	10.1	13.2	11.8	9.2	12.1	10.8	46.4	57.7	52.9
PPV 2% prev	29.9	40.2	35.7	11.5	16.7	14.3	33.4	45.6	40.4	18.6	23.6	21.3	17.0	21.8	19.6	63.6	73.4	69.4
PPV 3% prev	39.3	50.4	45.7	16.4	23.3	20.2	43.2	56.0	50.7	25.7	31.9	29.1	23.7	29.7	27.0	72.6	80.7	77.5
PPV 4% prev	46.5	57.8	53.1	21	29.1	25.4	50.6	63.1	58.1	31.8	38.6	35.6	29.5	36.3	33.2	78.1	84.9	82.2
PPV 5% prev	52.4	63.4	58.9	25.1	34.1	30.1	56.4	68.4	63.6	37	44.3	41.1	34.6	41.8	38.6	81.8	87.7	85.4
NPV 1% prev	99.4	99.6	99.5	99.6	99.9	99.8	99.4	99.7	99.5	99.7	99.9	99.8	99.7	99.9	99.8	99.6	99.9	99.7
NPV 2% prev	98.8	99.3	99.0	99.1	99.9	99.5	98.8	99.3	99.1	99.4	99.9	99.6	99.3	99.9	99.6	99.1	99.8	99.5

NPV 3% prev	98.2	98.9	98.6	98.7	99.8	99.3	98.2	99.0	98.6	99.0	99.8	99.4	99.0	99.8	99.4	98.7	99.7	99.2
NPV 4% prev	97.6	98.5	98.1	98.2	99.7	99.0	97.6	98.7	98.1	98.7	99.8	99.3	98.7	99.8	99.3	98.2	99.6	98.9
NPV 5% prev	96.9	98.2	97.6	97.8	99.7	98.8	96.9	98.3	97.7	98.3	99.7	99.1	98.3	99.7	99.1	97.8	99.5	98.7

AFP, alpha-fetoprotein; AFP-L3, *Lens culinaris* agglutinin-reactive AFP; BCLC, Barcelona Clinic Liver Cancer; CI, confidence interval; CLD, chronic liver disease; DCP, des-gamma carboxyprothrombin (PIVKA-II); GAAD, gender (biological sex), age, AFP, DCP; GALAD, gender (biological sex), age, AFP-L3, AFP, DCP; FN, false negatives; FP, false positives; HCC, hepatocellular carcinoma; NPV, negative predictive value; PIVKA-II, protein induced by vitamin K absence or antagonist II; PPV, positive predictive value; TN, true negatives; TP, true positives.

Table S5. Cut-offs of Elecsys assays, AFP, PIVKA-II and AFP-L3 and algorithmic scores GAAD (cobas) and GALAD (cobas), at specified sensitivity in STOP-HCC-MCE.

AFP															
	**AFP**	**Sensitivity all-stage**	**Sensitivity early-stage (BCLC 0/A)**	**Sensitivity late-stage (BCLC B–D)**	**Specificity**	**PPV 1%**	**PPV 2%**	**PPV 3%**	**PPV 4%**	**PPV 5%**	**NPV 1%**	**NPV 2%**	**NPV 3%**	**NPV 4%**	**NPV 5%**
**Sensitivity 70%**	6.94	70.2 (65.2–74.9)	59.2 (51.5–66.6)	80.2 (73.9–85.6)	86.8 (82.5–90.4)	5.1	9.8	14.1	18.1	21.9	99.7	99.3	98.9	98.6	98.2
**Sensitivity 75%**	5.88	75.1 (70.4–79.5)	65.5 (57.9–72.5)	83.9 (77.9–88.8)	83.5 (78.8–87.5)	4.4	8.5	12.3	15.9	19.3	99.7	99.4	99.1	98.8	98.5
**Sensitivity 80%**	5.03	80.1 (75.6–84.0)	71.8 (64.5–78.4)	87.5 (82.0–91.8)	76.9 (71.7–81.5)	3.38	6.6	9.7	12.6	15.4	99.7	99.5	99.2	98.9	98.7
**Sensitivity 85%**	4.23	85.2 (81.2–88.7)	78.2 (71.3–84.1)	91.7 (86.8–95.2)	70.3 (64.8–75.4)	2.82	5.53	8.15	10.7	13.1	99.8	99.6	99.4	99.1	98.9
**Sensitivity 90%**	3.62	90.2 (86.6–93.0)	84.5 (78.2–89.5)	95.3 (91.3–97.8)	63.4 (57.7–68.8)	2.43	4.78	7.07	9.3	11.5	99.8	99.7	99.5	99.4	99.2
**Sensitivity 95%**	2.21	95.1 (92.3–97.1)	92.5 (87.6–96.0)	97.4 (94.0–99.1)	26.4 (21.5–31.7)	1.29	2.57	3.84	5.11	6.37	99.8	99.6	99.4	99.2	99.0
**PIVKA-II**															
	**PIVKA-II**	**Sensitivity all-stage**	**Sensitivity early-stage (BCLC 0/A)**	**Sensitivity late-stage (BCLC B–D)**	**Specificity**	**PPV 1%**	**PPV 2%**	**PPV 3%**	**PPV 4%**	**PPV 5%**	**NPV 1%**	**NPV 2%**	**NPV 3%**	**NPV 4%**	**NPV 5%**

**Sensitivity 70%**	47.2	69.9 (65.0–74.6)	46.6 (39.0–54.3)	91.1 (86.2–94.8)	93.7 (90.4–96.2)	10.1	18.5	25.6	31.7	37.0	99.7	99.3	99.0	98.7	98.3
**Sensitivity 75%**	33.8	75.1 (70.4–79.5)	55.7 (48.0–63.3)	92.7 (88.1–96.0)	92.4 (88.8–95.1)	9.09	16.8	23.4	29.2	34.3	99.7	99.5	99.2	98.9	98.6
**Sensitivity 80%**	27.8	80.1 (75.6–84.0)	63.8 (56.2–70.9)	94.8 (90.6–97.5)	90.1 (86.2–93.2)	7.55	14.2	20	25.2	29.9	99.8	99.6	99.3	99.1	98.8
**Sensitivity 85%**	19.3	85.2 (81.2–88.7)	73.0 (65.7–79.4)	96.4 (92.6–98.5)	71.6 (66.2–76.6)	2.94	5.78	8.5	11.1	13.6	99.8	99.6	99.4	99.1	98.9
**Sensitivity 90%**	16.7	89.3 (85.7–92.3)	79.3 (72.5–85.1)	98.4 (95.5–99.7)	45.9 (40.2–51.7)	1.64	3.26	4.86	6.44	7.99	99.8	99.5	99.3	99.0	98.8
**Sensitivity 95%**	14.0	95.4 (92.7–97.3)	90.2 (84.8–94.2)	100 (98.1–100)	19.5 (15.2–24.4)	1.18	2.36	3.53	4.7	5.87	99.8	99.5	99.3	99.0	98.8
**AFP-L3**															
	**AFP-L3**	**Sensitivity all-stage**	**Sensitivity early-stage (BCLC 0/A)**	**Sensitivity late-stage (BCLC B–D)**	**Specificity**	**PPV 1%**	**PPV 2%**	**PPV 3%**	**PPV 4%**	**PPV 5%**	**NPV 1%**	**NPV 2%**	**NPV 3%**	**NPV 4%**	**NPV 5%**
**Sensitivity 70%**	1.2	100 (99.0–100)	100 (97.9–100)	100 (98.1–100)	0 (0.0–1.22)	1	2	3	4	5	-	-	-	-	-
**Sensitivity 75%**	1.2	100 (99.0–100)	100 (97.9–100)	100 (98.1–100)	0 (0.0–1.22)	1	2	3	4	5	-	-	-	-	-
**Sensitivity 80%**	1.2	100 (99.0–100)	100 (97.9–100)	100 (98.1–100)	0 (0.0–1.22)	1	2	3	4	5	-	-	-	-	-
**Sensitivity 85%**	1.2	100 (99.0–100)	100 (97.9–100)	100 (98.1–100)	0 (0.0–1.22)	1	2	3	4	5	-	-	-	-	-

**Sensitivity 90%**	1.2	100 (99.0–100)	100 (97.9–100)	100 (98.1–100)	0 (0.0–1.22)	1	2	3	4	5	-	-	-	-	-
**Sensitivity 95%**	1.2	100 (99.0–100)	100 (97.9–100)	100 (98.1–100)	0 (0.0–1.22)	1	2	3	4	5	-	-	-	-	-
**GAAD**															
	**GAAD**	**Sensitivity all-stage**	**Sensitivity early-stage (BCLC 0/A)**	**Sensitivity late-stage (BCLC B–D)**	**Specificity**	**PPV 1%**	**PPV 2%**	**PPV 3%**	**PPV 4%**	**PPV 5%**	**NPV 1%**	**NPV 2%**	**NPV 3%**	**NPV 4%**	**NPV 5%**
**Sensitivity 70%**	6.12	70.2 (65.2–74.9)	50.6 (42.9–58.2)	88.0 (82.6–92.3)	98.7 (96.7–99.6)	34.9	52.1	62.2	68.9	73.7	99.7	99.4	99.1	98.8	98.4
**Sensitivity 75%**	4.46	75.1 (70.4–79.5)	57.5 (49.8–64.9)	91.1 (86.2–94.8)	98.0 (95.7–99.3)	27.7	43.6	54.0	61.3	66.6	99.7	99.5	99.2	99.0	98.7
**Sensitivity 80%**	3.45	80.1 (75.6–84.0)	63.8 (56.2–70.9)	94.8 (90.6–97.5)	96.0 (93.2–97.9)	17.0	29.2	38.5	45.7	51.5	99.8	99.6	99.4	99.1	98.9
**Sensitivity 85%**	1.88	85.0 (80.9–88.5)	73.0 (65.7–79.4)	95.8 (92.0–98.2)	89.4 (85.4–92.7)	7.52	14.1	19.9	25.1	29.7	99.8	99.7	99.5	99.3	99.1
**Sensitivity 90%**	1.15	89.9 (86.3–92.8)	81.6 (75.0–87.1)	97.4 (94.0–99.1)	84.8 (80.3–88.7)	5.64	10.8	15.5	19.8	23.8	99.9	99.8	99.6	99.5	99.4
**Sensitivity 95%**	0.683	94.8 (92.0–96.8)	90.8 (85.5–94.7)	98.4 (95.5–99.7)	71.6 (66.2–76.6)	3.26	6.38	9.36	12.2	15.0	99.9	99.9	99.8	99.7	99.6
**GALAD**															
	**GALAD**	**Sensitivity all-stage**	**Sensitivity early-stage (BCLC 0/A)**	**Sensitivity late-stage (BCLC B–D)**	**Specificity**	**PPV 1%**	**PPV 2%**	**PPV 3%**	**PPV 4%**	**PPV 5%**	**NPV 1%**	**NPV 2%**	**NPV 3%**	**NPV 4%**	**NPV 5%**

**Sensitivity 70%**	6.3	70.2 (65.2–74.9)	50.6 (42.9–58.2)	88.0 (82.6–92.3)	98.7 (96.6–99.6)	34.8	51.9	62.0	68.8	73.6	99.7	99.4	99.1	98.8	98.4
**Sensitivity 75%**	4.77	75.1 (70.4–79.5)	57.5 (49.8–64.9)	91.1 (86.2–94.8)	98.0 (95.7–99.3)	27.6	43.5	53.8	61.1	66.5	99.7	99.5	99.2	99.0	98.7
**Sensitivity 80%**	3.51	79.8 (75.3–83.8)	63.8 (56.2–70.9)	94.3 (90.0–97.1)	96.3 (93.6–98.2)	18.1	30.8	40.3	47.6	53.5	99.8	99.6	99.4	99.1	98.9
**Sensitivity 85%**	1.99	85.2 (81.2–88.7)	73.6 (66.4–79.9)	95.8 (92.0–98.2)	90.0 (86.1–93.2)	7.95	14.9	20.9	26.3	31.0	99.8	99.7	99.5	99.3	99.1
**Sensitivity 90%**	1.2	89.9 (86.3–92.8)	81.6 (75.0–87.1)	97.4 (94.0–99.1)	84.4 (79.8–88.3)	5.5	10.5	15.1	19.3	23.3	99.9	99.8	99.6	99.5	99.4
**Sensitivity 95%**	0.689	94.8 (92.0–96.8)	90.8 (85.5–94.7)	98.4 (95.5–99.7)	71.1 (65.6–76.2)	3.21	6.27	9.21	12.0	14.7	99.9	99.9	99.8	99.7	99.6

AFP, alpha-fetoprotein; AFP-L3, *Lens culinaris* agglutinin-reactive AFP; BCLC, Barcelona Clinic Liver Cancer; DCP, des-gamma carboxyprothrombin (PIVKA-II); GAAD, gender (biological sex), age, AFP, DCP; GALAD, gender (biological sex), age, AFP-L3, AFP, DCP; HCC, hepatocellular carcinoma; NPV, negative predictive value; PIVKA-II, protein induced by vitamin K absence or antagonist II; PPV, positive predictive value.

Table S6. Cut-offs of Elecsys assays, AFP, PIVKA-II and AFP-L3 and algorithmic scores GAAD (cobas) and GALAD (cobas) at specified specificity in STOP-HCC-MCE.

**AFP**															
	**AFP**	**Sensitivity all-stage**	**Sensitivity early-stage (BCLC 0/A)**	**Sensitivity late-stage (BCLC B–D)**	**Specificity**	**PPV 1%**	**PPV 2%**	**PPV 3%**	**PPV 4%**	**PPV 5%**	**NPV 1%**	**NPV 2%**	**NPV 3%**	**NPV 4%**	**NPV 5%**
**Specificity 70%**	4.17	85.5 (81.5–89.0)	78.2 (71.3–84.1)	92.2 (87.4–95.6)	70.0 (64.5–75.1)	2.8	5.5	8.1	10.6	13.0	99.8	99.6	99.4	99.1	98.9
**Specificity 75%**	4.69	81.4 (77.1–85.3)	73.6 (66.4–79.9)	88.5 (83.2–92.7)	74.9 (69.6–79.7)	3.2	6.2	9.1	11.9	14.6	99.8	99.5	99.2	99.0	98.7
**Specificity 80%**	5.33	78.4 (73.8–82.5)	69.0 (61.5–75.7)	87.0 (81.4–91.4)	79.5 (74.6–83.9)	3.7	7.3	10.6	13.8	16.8	99.7	99.4	99.2	98.9	98.6
**Specificity 85%**	6.09	73.0 (68.1–77.4)	61.5 (53.8–68.8)	83.3 (77.3–88.3)	84.8 (80.3–88.7)	4.6	8.9	12.9	16.7	20.2	99.7	99.4	99.0	98.7	98.3
**Specificity 90%**	8.51	67.2 (62.1–72.0)	58.0 (50.3–65.5)	75.5 (68.8–81.4)	89.8 (85.8–92.9)	6.2	11.8	16.9	21.5	25.7	99.6	99.3	98.9	98.5	98.1
**Specificity 95%**	12.8	60.4 (55.2–65.4)	49.4 (41.8–57.1)	70.3 (63.3–76.7)	94.7 (91.6–97.0)	10.4	18.9	26.1	32.3	37.6	99.6	99.2	98.7	98.3	97.8
**PIVKA-II**															
	**PIVKA-II**	**Sensitivity all-stage**	**Sensitivity early-stage (BCLC 0/A)**	**Sensitivity late-stage (BCLC B–D)**	**Specificity**	**PPV 1%**	**PPV 2%**	**PPV 3%**	**PPV 4%**	**PPV 5%**	**NPV 1%**	**NPV 2%**	**NPV 3%**	**NPV 4%**	**NPV 5%**

**Specificity 70%**	19.1	85.5 (81.5–89.0)	73.0 (65.7–79.4)	96.9 (93.3–98.8)	70.0 (64.5–75.1)	2.8	5.5	8.1	10.6	13.0	99.8	99.6	99.4	99.1	98.9
**Specificity 75%**	20.2	85.0 (80.9–88.5)	72.4 (65.1–78.9)	96.4 (92.6–98.5)	74.9 (69.6–79.7)	3.3	6.5	9.5	12.4	15.1	99.8	99.6	99.4	99.2	99.0
**Specificity 80%**	21.7	83.9 (79.7–87.5)	70.7 (63.3–77.3)	95.8 (92.0–98.2)	80.2 (75.3–84.5)	4.1	8.0	11.6	15.0	18.2	99.8	99.6	99.4	99.2	99.0
**Specificity 85%**	23.4	82.2 (77.9–86.0)	68.4 (60.9–75.2)	94.8 (90.6–97.5)	85.1 (80.6–89.0)	5.3	10.2	14.6	18.7	22.6	99.8	99.6	99.4	99.1	98.9
**Specificity 90%**	27.5	80.3 (75.9–84.3)	64.4 (56.8–71.5)	94.8 (90.6–97.5)	89.8 (85.8–92.9)	7.4	13.8	19.5	24.7	29.2	99.8	99.6	99.3	99.1	98.9
**Specificity 95%**	58.9	67.5 (62.4–72.3)	42.0 (34.5–49.7)	90.6 (85.6–94.3)	95.0 (92.0–97.2)	12.1	21.8	29.7	36.2	41.8	99.7	99.3	99.0	98.6	98.2
**AFP-L3**															
	**AFP-L3**	**Sensitivity all-stage**	**Sensitivity early-stage (BCLC 0/A)**	**Sensitivity late-stage (BCLC B–D)**	**Specificity**	**PPV 1%**	**PPV 2%**	**PPV 3%**	**PPV 4%**	**PPV 5%**	**NPV 1%**	**NPV 2%**	**NPV 3%**	**NPV 4%**	**NPV 5%**
**Specificity 70%**	1.2	100 (99.0–100)	100 (97.9–100)	100 (98.1–100)	0 (0–1.22)	1	2	3	4	5	-	-	-	-	-
**Specificity 75%**	1.2	100 (99.0–100)	100 (97.9–100)	100 (98.1–100)	0 (0–1.22)	1	2	3	4	5	-	-	-	-	-
**Specificity 80%**	1.2	100 (99.0–100)	100 (97.9–100)	100 (98.1–100)	0 (0–1.22)	1	2	3	4	5	-	-	-	-	-
**Specificity 85%**	1.2	100 (99.0–100)	100 (97.9–100)	100 (98.1–100)	0 (0–1.22)	1	2	3	4	5	-	-	-	-	-

**Specificity 90%**	1.23	64.2 (59.1–69.1)	52.3 (44.6–59.9)	75 (68.3–81.0)	89.7 (85.7–92.9)	5.9	11.3	16.2	20.6	24.7	99.6	99.2	98.8	98.4	97.9
**Specificity 95%**	1.51	61.2 (56.0–66.2	47.7 (40.1–55.4)	73.4 (66.6–79.5)	94.4 (91.1–96.7)	9.9	18.1	25.1	31.1	36.3	99.6	99.2	98.7	98.3	97.9
**GAAD**															
	**GAAD**	**Sensitivity all-stage**	**Sensitivity early-stage (BCLC 0/A)**	**Sensitivity late-stage (BCLC B–D)**	**Specificity**	**PPV 1%**	**PPV 2%**	**PPV 3%**	**PPV 4%**	**PPV 5%**	**NPV 1%**	**NPV 2%**	**NPV 3%**	**NPV 4%**	**NPV 5%**
**Specificity 70%**	0.654	95.6 (93.0–97.5)	92.5 (87.6–96.0)	98.4 (95.5–99.7)	70.3 (64.8–75.4)	3.2	6.2	9.1	11.8	14.5	99.9	99.9	99.8	99.7	99.7
**Specificity 75%**	0.778	93.7 (90.7–96.0)	88.5 (82.8–92.8)	98.4 (95.5–99.7)	74.9 (69.6–79.7)	3.6	7.1	10.4	13.5	16.4	99.9	99.8	99.7	99.7	99.6
**Specificity 80%**	1.01	90.7 (87.3–93.5)	82.8 (76.3–88.1)	97.9 (94.8–99.4)	79.9 (74.9–84.2)	4.4	8.4	12.2	15.8	19.2	99.9	99.8	99.6	99.5	99.4
**Specificity 85%**	1.21	89.6 (86.0–92.5)	81.0 (74.4–86.6)	97.4 (94.0–99.1)	85.1 (80.6–89.0)	5.8	11.0	15.7	20.1	24.1	99.9	99.8	99.6	99.5	99.4
**Specificity 90%**	2.0	84.7 (80.6–88.2)	72.4 (65.1–78.9)	95.8 (92.0–98.2)	89.8 (85.8–92.9)	7.7	14.5	20.4	25.6	30.3	99.8	99.7	99.5	99.3	99.1
**Specificity 95%**	2.83	82.5 (78.2–86.3)	69.0 (61.5–75.7)	94.8 (90.6–97.5)	94.7 (91.6–97.0)	13.6	24.2	32.6	39.4	45.1	99.8	99.6	99.4	99.2	99.0
**GALAD**															
	**GALAD**	**Sensitivity all-stage**	**Sensitivity early-stage (BCLC 0/A)**	**Sensitivity late-stage (BCLC B–D)**	**Specificity**	**PPV 1%**	**PPV 2%**	**PPV 3%**	**PPV 4%**	**PPV 5%**	**NPV 1%**	**NPV 2%**	**NPV 3%**	**NPV 4%**	**NPV 5%**

**Specificity 70%**	0.655	95.4 (92.7–97.3)	92.0 (86.9–95.5)	98.4 (95.5–99.7)	69.8 (64.2–74.9)	3.1	6.1	8.9	11.6	14.2	99.9	99.9	99.8	99.7	99.7
**Specificity 75%**	0.797	94.0 (91.0–96.2)	89.1 (83.5–93.3)	98.4 (95.5–99.7)	75.1 (69.8–79.9)	3.7	7.2	10.4	13.6	16.6	99.9	99.8	99.8	99.7	99.6
**Specificity 80%**	1.04	90.7 (87.3–93.5)	83.3 (76.9–88.5)	97.4 (94.0–99.1)	80.1 (75.1–84.4)	4.4	8.5	12.3	15.9	19.3	99.9	99.8	99.6	99.5	99.4
**Specificity 85%**	1.22	89.9 (86.3–92.8)	81.6 (75.0–87.1)	97.4 (94.0–99.1)	85.0 (80.5–88.9)	5.7	10.9	15.7	20.0	24.0	99.9	99.8	99.6	99.5	99.4
**Specificity 90%**	1.82	86.6 (82.7–89.9)	76.4 (69.4–82.5)	95.8 (92.0–98.2)	90.0 (86.1–93.2)	8.1	15.1	21.2	26.6	31.4	99.9	99.7	99.5	99.4	99.2
**Specificity 95%**	2.83	82.0 (77.6–85.8)	67.8 (60.3–74.7)	94.8 (90.6–97.5)	95.0 (91.9–97.2)	14.2	25.1	33.7	40.7	46.4	99.8	99.6	99.4	99.2	99.0

AFP, alpha-fetoprotein; AFP-L3, *Lens culinaris* agglutinin-reactive AFP; BCLC, Barcelona Clinic Liver Cancer; DCP, des-gamma carboxyprothrombin (PIVKA-II); GAAD, gender (biological sex), age, AFP, DCP; GALAD, gender (biological sex), age, AFP-L3, AFP, DCP; HCC, hepatocellular carcinoma; NPV, negative predictive value; PIVKA-II, protein induced by vitamin K absence or antagonist II; PPV, positive predictive value.
