# Supplementary material for: Comparative evaluation of multimarker algorithms for early-stage HCC detection in multicenter prospective studies
Source: JHEP Rep. 2024 Nov 8;7(2):101263. doi: 10.1016/j.jhepr.2024.101263 (PMC11782856; doi:10.1016/j.jhepr.2024.101263)
Supplement: Multimedia component 2 [file mmc2.docx]

**JHEP Reports**

**CTAT methods**

**If the CTAT form is not relevant to your study, please outline the reasons why:**

| Biological samples were obtained from study participants, not from a vendor. |
| --- |

- 1. **Software**

| **Software name** | **Manufacturer** | **Version** |
| --- | --- | --- |
| **R** | **R Core Team** | **Version 4.1.3** |

- 1. **Please provide the details of the corresponding methods author for the manuscript:**

| **Konstantin Kroeniger**  Clinical Algorithms & Biomarker Statistics  Roche Diagnostics GmbH  Nonnenwald 2  82377 Penzberg  Germany  Email: konstantin.kroeniger@roche.com |
| --- |
