# Supplementary material for: Comparative evaluation of multimarker algorithms for early-stage HCC detection in multicenter prospective studies
Source: JHEP Rep. 2024 Nov 8;7(2):101263. doi: 10.1016/j.jhepr.2024.101263 (PMC11782856; doi:10.1016/j.jhepr.2024.101263)
Supplement: Multimedia component 4 [file mmc4.pdf]

Comparative evaluation of multimarker algorithms for early-stage HCC detection in multicenter prospective studies☆

Jinlin Hou[1], Thomas Berg[2], Arndt Vogel[3,4,5], Teerha Piratvisuth[6], Jörg Trojan[7], Enrico N. De Toni[8], Masatoshi Kudo[9], Katarina Malinowsky[10], Peter Findeisen[11], Johannes Kolja Hegel[12], Wenzel Schöning[13], Kairat Madin[14], Konstantin Kroeniger[15], Henry Lik-Yuen Chan[16,‡,*], Ashish Sharma[17,‡,*]

JHEP Reports **2025**. vol. 7 | 1–12

Check for updates

Background & Aims: We compared the clinical performance of the novel GAAD (gender [biological sex], age, alpha-fetoprotein [AFP], des-gamma carboxyprothrombin [DCP]) and GALAD (gender [biological sex], age, AFP, *Lens culinaris* agglutinin-reactive AFP [AFP-L3], DCP) algorithms to deduce the clinical utility of AFP-L3 for detecting early-stage hepatocellular carcinoma (HCC) from chronic liver disease (CLD).

Methods: An algorithm development study (STOP-HCC-ARP) and clinical validation study (STOP-HCC-MCE) were conducted, recruiting adult participants with HCC (confirmed by radiology or pathology) or CLD in an international, multicenter, case-control design. Serum biomarkers were measured using Elecsys assays (GAAD and GALAD [Cobas]) or µTASWAKO assays (GALAD [µTASWAKO]) while blinded to case/control status.

Results: In STOP-HCC-ARP (algorithm development cohort), 1,006 patients {297 HCC (41.4% early-stage [Barcelona Clinic Liver Cancer {BCLC} 0/A) and 709 CLD} were included. Area under the curve (AUCs) for discriminating between early-stage HCC *vs.* CLD were 91.4%, 91.4%, and 90.8% for GAAD (Cobas), GALAD (Cobas), and GALAD (µTASWAKO), respectively. The clinical validation cohort of STOP-HCC-MCE comprised 1,142 patients, (366 HCC cases [48% early-stage], 468 specificity samples and 302 CLD); AUCs for GAAD (Cobas), GALAD (Cobas), and GALAD (µTASWAKO) for discriminating between early-stage HCC *vs.* CLD were 91.4%, 91.5%, and 91.0%, respectively; AUCs were 94.7–95.0% for all-stage HCC. The GAAD and GALAD algorithms demonstrated similar good performance regardless of disease etiology, presence of cirrhosis, geographical region, and within pan-tumor specificity panels (p <0.001).

Conclusions: GAAD (Cobas) demonstrated good clinical performance, similar to GALAD (Cobas and µTASWAKO) algorithms, in differentiating HCC and CLD controls, across all disease stages, etiologies, and regions; therefore, AFP-L3 may have a negligible role in GALAD for HCC surveillance.

© 2024 The Authors. Published by Elsevier B.V. on behalf of European Association for the Study of the Liver (EASL). This is an open access article under the CC BY license (http://creativecommons.org/licenses/by/4.0/).

Introduction

Early hepatocellular carcinoma (HCC) surveillance is essential to improve clinical outcomes.[1,2] Therefore, surveillance programs, including ultrasonography (USG) every 6 months with or without alpha-fetoprotein (AFP) testing, are recommended to screen at-risk patients.[1,3–6] Risk factors for developing HCC include cirrhosis, hepatitis B and C virus (HBV, HCV) infection, metabolic dysfunction-associated steatotic liver disease (MASLD), and alcoholic-related liver disease (ALD).[2]

Although surveillance programs are guideline-recommended, the combination of USG + AFP may only identify up to 70% of patients with early-stage HCC.[1,4,5,7] In addition to AFP, other HCC-specific serum biomarkers, such as protein induced by vitamin K absence or antagonist-II (PIVKA-II) and *Lens culinaris* agglutinin-reactive AFP (AFP-L3), have been identified.[8] However, when used individually, serum biomarkers demonstrate inadequate sensitivity and accuracy for HCC diagnosis.[1,3–6,8] Notably, a recent Asia-Pacific consensus paper advised that PIVKA-II in combination with AFP (and USG) shows potential benefit for HCC detection especially in those with small and AFP-negative tumors, which are predominantly early-stage disease.[9–11]

To further improve the detection of early-stage HCC from benign chronic liver disease (CLD), algorithms combining demographic characteristics and serum biomarkers have also been developed.[8,12–14] Notably, the GALAD score, combining gender (biological sex) and age plus a three-serum biomarker

☆ Given their role as Co-Editor, Arndt Vogel had no involvement in the peer-review of this article and had no access to information regarding its peer-review. Full responsibility for the editorial process for this article was delegated to the Guest Editor Tim Meyer.

* Corresponding authors. Address: Faculty of Medicine, Choh-Ming Li Basic Medical Sciences Building, The Chinese University of Hong Kong, 32 Tai Po Road, Ma Liu Shui, Hong Kong Special Administrative Region of China, 999077 (H.L.Y. Chan); Clinical Development and Medical Affairs, Roche Diagnostics International AG, Forrenstrasse 2, 6343 Risch-Rotkreuz, Switzerland (A. Sharma).

E-mail addresses: hlychan@cuhk.edu.hk (H. Lik-Yuen Chan), ashish.sharma.as6@roche.com (A. Sharma).

‡ These authors shared equal contribution.

https://doi.org/10.1016/j.jhepr.2024.101263

panel (AFP-L3, AFP, and PIVKA-II), has demonstrated good clinical performance for the differentiation of HCC and CLD, superior to that of single biomarkers in multiple case-control and prospective phase II/III biomarker studies.[8,12–16] Although AFP-L3 is included in GALAD, the first algorithm development study reported odds ratios of 1.05 and 1.04 for AFP-L3 in the discovery and validation datasets, respectively, with near zero coefficients; thus, AFP-L3 may contribute negligibly to the GALAD algorithm.[14]

Years later, the role and contribution of AFP-L3 in HCC detection remains controversial as a standalone assay and component of GALAD owing to evolving disease etiologies and antiviral treatment paradigms. Viral hepatitis-related HCC has plateaued globally, whereas ALD and MASLD-related HCC incidence and mortality have increased,[17–19] highlighting a need to closely examine the utility of AFP-L3 and GALAD in the evolving high-risk population. Because of advances in antiviral HBV/HCV therapies leading to improved liver function, post-treatment AFP levels stay normalized, rendering high specificity to AFP for HCC surveillance in patients with chronic inflammatory background,[20] potentially making AFP-L3 usage obsolete.[21] Therefore, novel Elecsys-based GAAD (gender [biological sex], age, AFP, DCP [des-gamma carboxyprothrombin {PIVKA-II}]) and GALAD algorithms (Roche Diagnostics International Ltd, Rotkreuz, Switzerland) have been developed. The GAAD algorithm does not include AFP-L3 and demonstrated good clinical performance in HCC and benign CLD differentiation.[22]

In this study, we compared the clinical performance of the GAAD and GALAD algorithms to deduce the clinical utility of AFP-L3 in the context of disease etiology shift and current treatment patterns in two large-scale prospective studies.

Experimental procedures

Study population

Two independent, international, multicenter, prospective cohort studies were enrolled using a case-control design in participants aged ≥18 years, comprising cohorts of HCC (early-stage [Barcelona Clinic Liver Cancer {BCLC} tumor stage 0/A], late-stage [BCLC tumor stage B–D], and all-stage), and a benign CLD control. The STOP-HCC-ARP algorithm development study involved seven clinics across Germany, Spain, Thailand, and Hong Kong Special Administrative Region of China (SAR) between 2014 and 2016; the study design and biomarker selection methodology have been previously described (Fig. 1A).[8] The STOP-HCC-MCE clinical validation study involved 10 clinics from the People's Republic of China, Hong Kong (SAR), Germany, Thailand, and Japan from 2017 to 2022. The study data were included in clinical performance analysis and a specificity panel.

Eligible HCC patients had a first-time HCC diagnosis, confirmed either by radiology according to international guidelines (USG, or either a ≥1 cm lesion showing arterial-phase hyperenhancement in combination with washout appearance and/or capsule by quadruple-phase computed tomography scan or multiphase contrast-enhanced magnetic resonance imaging)[1,5,23] or by positive pathology within 6 months of enrollment. Briefly, the BCLC staging system was used to categorize patients with HCC into one of the following disease stages: very early HCC (stage 0; single nodule ≤2 cm without vascular invasion or extrahepatic spread in an asymptomatic patient with preserved liver function); early HCC (stage A; single nodule or ≥3 nodules <3 cm without macrovascular invasion or extrahepatic spread in an asymptomatic patient [performance status {PS} 0]); intermediate HCC (stage B; multifocal HCC with no vascular invasion or extrahepatic spread in an asymptomatic patient with preserved liver function [PS 0]); advanced HCC (stage C; patients with vascular invasion or extrahepatic spread presenting with PS ≥2 and preserved liver function); end-stage (stage D; major cancer-related symptoms [PS >2] and/or impaired liver function without the option of liver transplant owing to HCC burden or non–HCC-related factors). Eligible CLD controls comprised at-risk patients without HCC confirmed by imaging within the past 12 months, and the presence of cirrhotic or non-cirrhotic liver disease of either viral (HBV/HCV infection) or non-viral (ALD, metabolic dysfunction-associated steatohepatitis or other) etiology undergoing HCC surveillance.

Exclusion criteria was any other cancer (excluding non-melanoma skin cancer), recurrent HCC, HCC treatment, a glomerular filtration rate <60 ml/min/1.73 m^2, or treatment with anti-vitamin K coagulant therapy. Those in the CLD control group with a hepatic mass (either indeterminate or meeting radiological criteria for HCC) were also excluded.

For specificity panel analysis of STOP-HCC-MCE, diseases included in the specificity panel were grouped into the following categories: (1) other histologically-confirmed malignancies: cholangiocarcinoma, colorectal cancer, pancreatic cancer, gastric/esophageal cancer, gynecological cancers (ovarian, endometrial and cervical), lung cancer, renal cancer, breast cancer; (2) other benign liver diseases: hemangioma, benign liver cysts, other benign liver diseases (hepatocellular adenoma or focal nodular hyperplasia); and (3) benign diseases: rheumatoid arthritis, morbus Crohn, ulcerative colitis, other autoimmune disease (systemic lupus erythematosus and Hashimoto thyroiditis). To note, patients with cholangiocarcinoma and pancreatic cancer were excluded from the analysis.

Ethics committee and institutional review board approvals were obtained for all study sites involved in sample collection. Each participant provided informed consent before enrollment, and local rules regarding informed consent for the subsequent use of collected samples were followed.

GAAD and GALAD (Cobas) algorithm development

Multivariate analyses were performed to identify the best-performing biomarker panel that could separate early- or all-stage HCC from benign controls using two methods: (1) lasso regression (no fixed panel size) and (2) exhaustive search with logistic regression (fixed panel size [from two to four biomarkers]). Lasso regression optimized the best model by maximizing diagnostic accuracy while minimizing the biomarker number. In an exhaustive search, logistic regression models, based on all possible two to four biomarker combinations, were evaluated and compared. For the two top-performing clinical algorithms (GAAD and GALAD), the full STOP-HCC-ARP dataset was used to train logistic regression models, with an HCC diagnosis (BCLC-stage-independent) used as the predictor variable. After training the models, the GAAD and GALAD cut-offs were determined by calculating the 90% quantile of the score values (using the type 2 method, described previously[24]) from the control patients of STOP-HCC-ARP, which corresponds to the 90% specificity cut-off for the aid in diagnosis of early-stage HCC.

Biomarker evaluation

Serum samples were collected ≥1 day before any planned procedures requiring general anesthesia and stored at -70 °C at the sample collecting sites. Samples were tested at Microcoat GmbH (Bernried, Germany), with the exception of samples collected in the People's Republic of China (Elecsys assays were tested at the collection site and µTASWAKO assays were tested at Huashan Hospital, Shanghai, China).

PIVKA-II, AFP, and AFP-L3 serum levels were measured in one run within 3 experimental days using either Elecsys assays on the Cobas e 601 analyzer, or in multiple smaller batches in more than 3 days using µTASWAKO assays on the Fujifilm

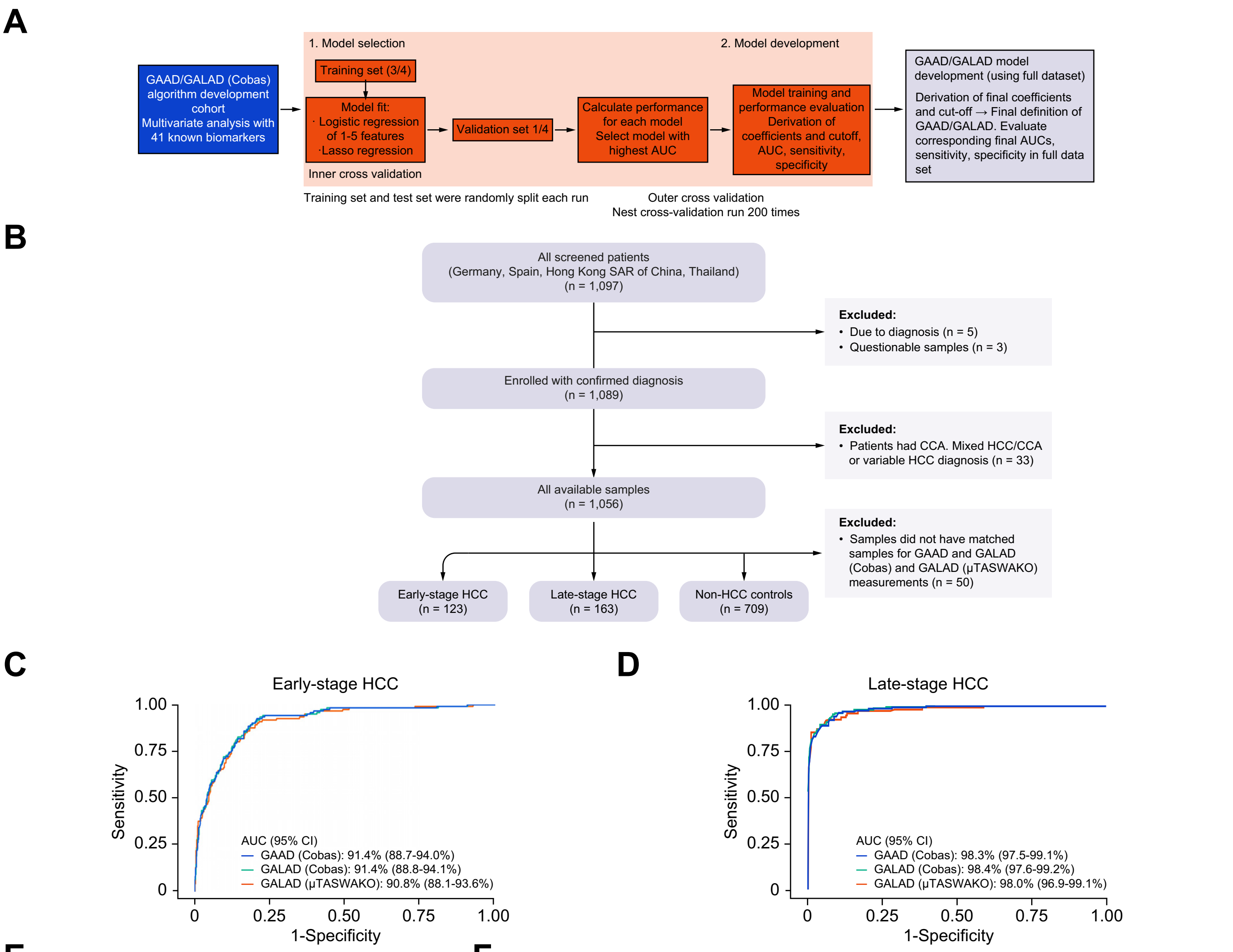

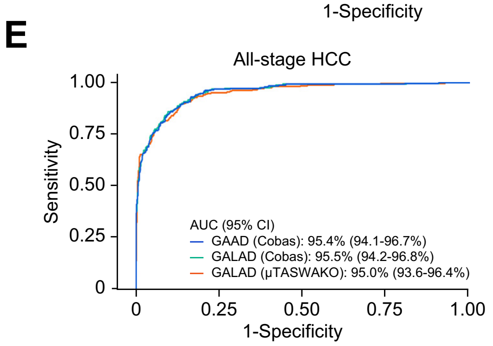

F

Algorithm (cut-off score)	Sensitivity, % (95% CI)			Specificity, % (95% CI)
	Early-stage HCC (BCLC 0/A) (n = 123)	Late-stage HCC (BCLC B-D) (n = 163)	All-stage HCC (n = 297)	CLD controls (n = 709)
GAAD (Cobas, 2.57)	71.5 (62.7-79.3)	95.1 (90.6-97.9)	85.2 (80.6-89.0)	90.1 (87.7-92.2)
GALAD (Cobas, 2.47)	72.4 (63.6-80.0)	96.3 (92.2-98.6)	86.2 (81.7-89.9)	89.8 (87.4-92.0)
GALAD (µTASWAKO, -0.63)	58.5 (49.3-67.3)	92.0 (86.7-95.7)	77.8 (72.6-82.4)	94.1 (92.1-95.7)
GALAD (µTASWAKO, -1.23)*	69.1 (60.1-77.1)	93.3 (88.2-96.6)	83.2 (78.4-87.2)	90.0 (87.5-92.1)

Fig. 1. Development, clinical evaluation, and performance of GAAD (Cobas), GALAD (Cobas), and GALAD (µTASWAKO) algorithmic scores in STOP-HCC-ARP. Algorithm development strategy (A) and study disposition (B); clinical performance in differentiating early-stage (C); late-stage (D) and all-stage HCC (E) from CLD controls. Sensitivities and specificities are shown in table (F). *Cut-off value corresponds to matching GAAD (Cobas) specificity of 90%. AUC, area under the curve; BCLC, Barcelona Clinic Liver Cancer; CCA, cholangiocarcinoma; CLD, chronic liver disease; GAAD, gender (biological sex), age, AFP, DCP (PIVKA-II); GALAD, gender (biological sex), age, AFP-L3, AFP, DCP (PIVKA-II); HCC, hepatocellular carcinoma.

Micro Total Analysis System µTASWako analyzer owing to lower throughput of the µTASWako analyzer. Sample stability studies were conducted for all assays in accordance with the regulatory guidelines for an *in vitro* diagnostic medical device registration.

Predefined established cut-offs (based on the training cohort) used for the detection of HCC *vs.* benign CLD were: 20 ng/ml for AFP (Elecsys); 2.3 ng/ml for AFP-L3 (Elecsys); 28.4 ng/ml for PIVKA-II (Elecsys); 2.47 (range 0–10) for GALAD (Cobas); 2.57 (range 0–10) for GAAD (Cobas); and –0.63 for GALAD (µTASWAKO).

Additional cut-offs for GALAD (µTASWAKO) were also assessed, corresponding to GAAD (Cobas) specificity of 90%.

Statistical analysis

The clinical performance of the GAAD and GALAD algorithms, and individual biomarkers alone, were compared using receiver operating characteristic (ROC) analysis, and area under the curve (AUC) values were calculated. For sensitivity and specificity analyses, the derived 95% confidence intervals (CIs) were calculated from the binomial distribution using the Clopper-Pearson method.[25] Values of p comparing non-inferiority of AUCs were calculated using the H0 hypothesis AUC (Test 1) < AUC (Test 2) - 0.01 with a studentized bootstrap approach using N = 1,000 bootstrap replicates.

For the specificity panel in the STOP-HCC-MCE study, p values were calculated using binomial tests, with Bonferroni correction for every marker using the cut-offs listed previously and a specificity value ≥90%. If the sample size in the disease group was too small (<10), subgroup analysis was not performed.

For further details regarding the materials and methods used, please refer to the Supplementary CTAT table.

Results

Algorithm development study, STOP-HCC-ARP

Study population

Of the 1,097 patients enrolled, 1,006 had samples available for inclusion in the algorithm development study based on matched sample availability (Fig. 1B). The demographics and characteristics of the participants are shown (Table 1). Of all HCC patients, 123 (41.4%) had early- and 163 (54.9%) had late-stage HCC. In the early-stage HCC, late-stage HCC, and CLD control groups, respectively, mean age (standard deviation [SD]) was 60.5 (9.5), 60.6 (9.9), and 55.3 (10.7) years; 71.5%, 85.3%, and 56.3% were male; and 84.6%, 74.2%, and 53.2% had cirrhosis.

Clinical performance

The AUCs of GAAD (Cobas), GALAD (Cobas), and GALAD (µTASWAKO) were comparable and demonstrated good clinical performance in discriminating between early- (91.4%, 91.4%, and 90.8%, respectively), late- (98.3%, 98.4%, and 98.0%) and all-stage HCC (95.4%, 95.5%, and 95.0%) *vs.* CLD controls (Fig. 1C–E). The sensitivity and specificities of the algorithms are shown (Fig. 1F). When using a cut-off for GALAD (µTASWAKO) corresponding to 90% specificity for GAAD (Cobas), performance was comparable to both GAAD and GALAD (Cobas).

The GAAD and GALAD algorithms performed well, and AUCs remained comparable across different HCC etiologies (viral or non-viral disease etiologies with or without cirrhosis) (Fig. S1). In early-, late-, and all-stage HCC, respectively, AUCs for viral and non-viral etiologies were 88.2–97.6%, 95.7–99.5%,

Table 1. Participant demographics and clinical characteristics in algorithm development study, STOP-HCC-ARP.

	Early-stage HCC (BCLC 0/A) (n = 123)	Late-stage HCC (BCLC B–D) (n = 163)	All-stage HCC (n = 297)*	CLD controls (n = 709)
**Patient characteristics**				
Age, years, mean (SD)	60.5 (9.5)	60.6 (9.9)	60.8 (9.8)	55.3 (10.7)
Sex, n (%)†				
Male	88 (71.5)	139 (85.3)	233 (78.5)	399 (56.3)
Female	34 (27.6)	24 (14.7)	63 (21.2)	309 (43.6)
**Liver biochemistry, n (SD)**				
AST, U/L, median (IQR)‡	44.6 (28.0–78.5)	74.5 (48.6–123.9)	58.4 (39.2–104.8)	28.9 (22.5–45.3)
ALT, U/L, median (IQR)‡	26.8 (16.2–44.6)	34.0 (21.8–63.3)	30.5 (19.0–53.1)	20.0 (13.7–32.2)
Serum albumin, g/L, median (IQR)§	39.8 (34.0–44.6)	37.0 (32.0–41.8)	38.0 (33.0–43.0)	44.0 (41.0–46.0)
Serum total bilirubin, µmol/L, median (IQR)¶	16.8 (10.3–27.4)	17.1 (11.8–28.0)	16.9 (10.3–27.4)	12.0 (8.6–17.0)
ALBI score, median (IQR)	–2.5 (–3.0–1.9)	–2.3 (–2.8–1.8)	–2.4 (–2.9–1.9)	–3.0 (–3.3–2.7)
**Etiology, n (%)****				
Cirrhosis	104 (84.6)	121 (74.2)	232 (78.1)	377 (53.2)
Cirrhosis viral	97 (78.9)	102 (62.6)	205 (69.0)	350 (49.4)
Cirrhosis non-viral	28 (22.8)	34 (20.9)	64 (21.5)	59 (8.3)
Non-cirrhosis	15 (12.2)	29 (17.8)	47 (15.8)	330 (46.5)
Non-cirrhosis viral	16 (13.0)	28 (17.2)	47 (15.8)	339 (47.8)
Non-cirrhosis non-viral	3 (2.4)	8 (4.9)	11 (3.7)	67 (9.4)
Other	4 (3.3)	13 (8)	18 (6.1)	2 (0.3)

ALBI, albumin–bilirubin; ALT, alanine transaminase; ALT, alanine transaminase; AST, aspartate transferase; BCLC, Barcelona Clinic Liver Cancer; CLD, chronic liver disease; HCC, hepatocellular carcinoma; MELD, model for end-stage liver disease.
*HCC stage was unknown for 11 patients.
†Missing data for one patient with early-stage HCC, and two patients in the non-HCC CLD control groups.
‡Missing data for five patients with HCC (two with early-stage; three with late-stage HCC), and 21 patients with non-HCC CLD.
§Missing data for 19 patients with HCC (10 with early-stage; nine with late-stage HCC), and 122 patients with non-HCC CLD.
¶Missing data for 20 patients with HCC (10 with early-stage; 10 with late-stage HCC), and 169 patients with non-HCC CLD.
**Patients could have multiple disease etiologies.

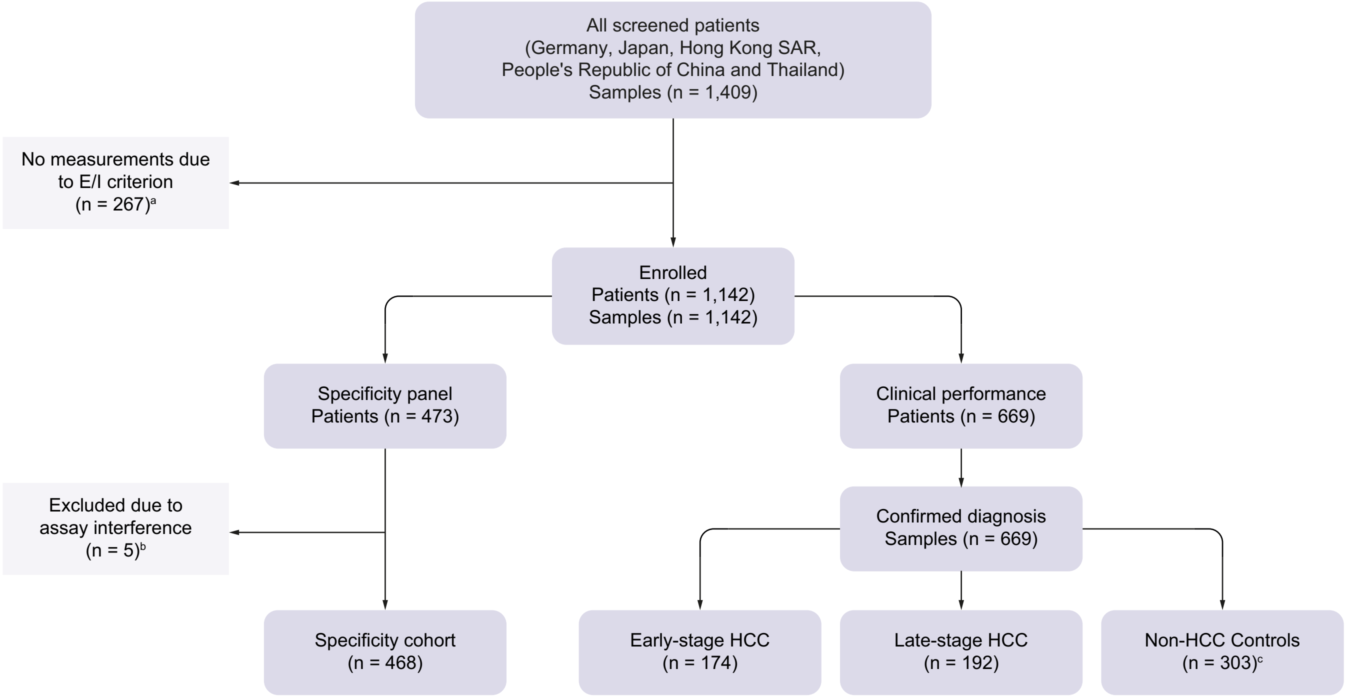

Fig. 2. Study design and sample disposition in STOP-HCC-MCE. [a]Excluded because of exclusion criteria (67 owing to renal failure, 17 owing to ICF issues, and 124 owing to lab parameters, sample processing, other cancer/missing diagnosis). [b]Excluded because of interferences with assays. [c]One non-HCC control subject was included for GAAD evaluation but excluded for GALAD evaluation due to bilirubin interference with AFP-L3 assay. AFP-L3, *Lens culinaris* agglutinin-reactive alpha-fetoprotein; E/I, exclusion/inclusion; GAAD, gender (biological sex), age, AFP, DCP (PIVKA-II); GALAD, gender (biological sex), age, AFP-L3, AFP, DCP (PIVKA-II); HCC, hepatocellular carcinoma; ICF, International Classification of Functioning, Disability and Health.

and 92.2–98.7%, and 90.0–97.1%, 98.5–100%, and 91.4–97.3%, respectively.

Clinical validation study, STOP-HCC-MCE

Study population

Patients enrolled and eligible for analysis in STOP-HCC-MCE (n = 1,142) were split into two cohorts; 668 evaluable participants (366 with HCC [174 early-stage; 192 late-stage] and 302 CLD controls) were included for analysis in the clinical performance cohort and 468 participants in the specificity panel cohort (Fig. 2).

Baseline characteristics of patients included in the clinical performance cohort are described (Table 2). In the early-stage HCC, late-stage HCC, and CLD control groups, respectively, mean age (SD) was 58.2 (11.4), 61.3 (11.2), and 49.6 (12.5) years; 83.9%, 84.4%, and 63.2% were male; 77.6%, 79.2%, and 37.1% had cirrhosis; and 88.5%, 66.7%, and 77.8% of patients had viral liver disease etiology (Table 2). Participant demographics and clinical characteristics for the specificity panel cohort, and the clinical performance cohort by clinical site, are shown (Tables S1 and S2, respectively).

Method comparison and specificity panel analysis

A method comparison using weighted Deming regression analyses indicated an excellent analytical agreement between GALAD (μTASWAKO) and GAAD (Cobas) (Pearson's r = 0.962; *p* <0.001), and GALAD (Cobas) (Pearson's r = 0.969; *p* <0.001) algorithms in this cohort (Fig. 3A). In the specificity panel cohort (n = 468), low scores for GAAD (Cobas), GALAD (Cobas), and GALAD (μTASWAKO) were observed across disease groups, indicating high algorithmic panel specificity for HCC diagnosis except slight elevation in gastrointestinal cancers (median [IQR 1.79 [0.90–2.83], *p* <0.01; 1.82 [0.96–2.90], *p* <0.01); and -2.61 [-3.42 to -1.38] for GAAD (Cobas), GALAD (Cobas), and GALAD (μTASWAKO), respectively] (Fig. 3B–D).

Clinical performance

The distribution of GAAD and GALAD (Cobas) scores was both distinctly higher in all-stage HCC *vs.* CLD (median [IQR]: GAAD: 9.05 [4.49–9.96] *vs.* 0.39 [0.16–0.77], *p* <0.001; GALAD: 9.24 [4.78–9.98] *vs.* 0.41 [0.18–0.80], *p* <0.001) (Fig. 4A). For each BCLC stage, GAAD and GALAD (Cobas) scores were significantly higher for HCC patients compared with CLD controls (*p* <0.001) (Fig. 4B). Within the HCC case group, GAAD and GALAD scores remained high, irrespective of disease etiology (Fig. 4C) and geographic region (Fig. 4D); overall, scores for GAAD and GALAD for the HCC group were significantly higher compared with CLD controls (*p* <0.001).The detection of different HCC stages and CLD controls using single markers (Elecsys AFP, AFP-L3, and PIVKA-II assays) is shown (Fig. S2).

GAAD (Cobas), GALAD (Cobas), and GALAD (μTASWAKO) were able to discriminate between early- (AUC 91.0–91.5%), late- (98.2–98.3%), and all-stage HCC (94.7–95.0%) from CLD controls (Fig. 5A–C). The sensitivity for GAAD (Cobas) and GALAD (Cobas) was 70.1% for both in early-stage, 94.8% and 95.3% in late-stage, and 83.1% and 83.3% in all-stage HCC, respectively, at 93.7% (GAAD [Cobas]) and 93.0% (GALAD

Table 2. Participant demographics and clinical characteristics in clinical validation study, STOP-HCC-MCE.

	Early-stage (BCLC 0/A) HCC (n = 174)	Late-stage (BCLC B–D) HCC (n = 192)	Non-HCC CLD controls (n = 302)
**Patient characteristics**			
Age, years, mean (SD)	58.2 (11.4)	61.3 (11.2)	49.6 (12.5)
Sex, n (%)			
Male	146 (83.9)	162 (84.4)	191 (63.2)
Female	28 (16.1)	30 (15.6)	111 (36.8)
Race, n (%)			
Asian	135 (77.6)	91 (47.4)	181 (59.9)
White	39 (22.4)	99 (51.6)	112 (37.1)
Black/African American	0	1 (0.5)	3 (1.0)
Other/missing	0	1 (0.5)	6 (2.0)
**Liver disease etiology, n (%)**			
Viral liver disease etiology	154 (88.5)	128 (66.7)	235 (77.8)
Antiviral therapy in patients with viral liver disease etiology	74 (48.1)	63 (49.2)	121 (51.4)
HBV	127 (73.0)	105 (54.7)	174 (57.6)
HCV	27 (15.5)	23 (12.0)	61 (20.2)
MASH	9 (5.2)	21 (10.9)	62 (20.5)
ALD	14 (8)	42 (21.9)	23 (7.6)
Other	27 (15.5)	34 (17.7)	76 (25.2)
Cirrhosis	135 (77.6)	152 (79.2)	112 (37.1)
Cirrhotic HBV	97 (55.7)	81 (42.2)	60 (19.9)
Cirrhotic HCV	22 (12.6)	22 (11.5)	16 (5.3)
Cirrhotic MASH	7 (4.0)	14 (7.3)	7 (2.3)
Cirrhotic ALD	14 (8.0)	40 (20.8)	19 (6.3)
Cirrhotic other	23 (13.2)	28 (14.6)	29 (9.6)
Non-cirrhosis	39 (22.4)	40 (20.8)	190 (62.9)
Non-cirrhotic HBV	30 (17.2)	24 (12.5)	114 (37.7)
Non-cirrhotic HCV	5 (2.9)	1 (0.5)	45 (14.9)
Non-cirrhotic MASH	2 (1.1)	7 (3.6)	55 (18.2)
Non-cirrhotic ALD	0	2 (1.0)	4 (1.3)
Non-cirrhotic other	4 (2.3)	6 (3.1)	47 (15.6)
**Liver biochemistry and clinical features**			
AST, U/L, median (IQR)	34.0 (25.0–47.0)	66.5 (41.0–119.0)	29.0 (21.0-41.0)
ALT, U/L, median (IQR)	30.0 (23.0–42.0)	42.0 (30.0–68.0)	27.8 (20.0–44.8)
Serum albumin, g/L, median (IQR)	39.4 (36.0–41.8)	35.8 (31.8–40.0)	44.0 (40.0–47.0)
Serum total bilirubin, µmol/L, median (IQR)	14.1 (10.8–20.8)	15.4 (11.0–26.6)	11.0 (8.0–16.3)
PT-INR, n (%)			
1	174 (100.0)	187 (97.4)	–
2/3	0	5 (2.6)	–
Ascites, n (%)			
Mild	12 (6.9)	42 (21.9)	8 (2.7)
Moderate to severe	4 (2.3)	17 (8.9)	4 (1.3)
None	158 (90.8)	133 (69.3)	290 (96.0)
Hepatic encephalopathy, n (%)			
Grade I–II	2 (1.2)	4 (2.1)	4 (1.3)
None	172 (98.8)	188 (97.9)	298 (98.7)
MELD score, median (IQR)	8.0 (7.0–9.0)	8.0 (7.0–10.0)	7.0 (6.0–8.0)
ALBI score, median (IQR)	–2.6 (–2.8–2.3)	–2.3 (–2.6–1.8)	–3.1 (–3.3–2.7)
ALBI grade, n (%)			
1	85 (48.9)	55 (28.6)	241 (79.8)
2	85 (48.9)	114 (59.4)	56 (18.5)
3	4 (2.3)	23 (12)	5 (1.7)
Child–Pugh class, n (%)			
A	154 (88.5)	129 (67.2)	–
B	20 (11.5)	54 (28.1)	–
C	0 (0)	9 (4.7)	–
Tumor nodule number, n (%)			
1	147 (84.5)	45 (23.4)	NA
2	23 (13.2)	35 (18.2)	NA
3	4 (2.3)	17 (8.9)	NA
≥3	0 (0)	95 (49.5)	NA

(*continued on next page*)

Table 2. (*continued*)

	Early-stage (BCLC 0/A) HCC (n = 174)	Late-stage (BCLC B–D) HCC (n = 192)	Non-HCC CLD controls (n = 302)
**Tumor characteristics, n (%)**			
Large multinodular	0 (0)	87 (45.3)	NA
Portal invasion or extrahepatic spread (N1, M1)	0 (0)	82 (42.7)	NA
Single <2 cm	43 (24.7)	0 (0)	NA
Single or ≤3 nodules ≤3 cm	131 (75.3)	23 (12.0)	NA
**Size of index lesion, cm, mean (IQR; minimum, maximum)**	3.0 (1.8–3.8; 0–11.3)	7.6 (4.5–10.0; 0.7–18.4)	NA

ALBI, albumin–bilirubin; ALD, alcoholic-related liver disease; ALT, alanine transaminase; AST, aspartate transferase; BCLC, Barcelona Clinic Liver Cancer; CLD, chronic liver disease; HBV, hepatitis B virus; HCC, hepatocellular carcinoma; HCV, hepatitis C virus; MASH, metabolic dysfunction-associated steatohepatitis; MELD, model for end-stage liver disease; PT-INR, prothrombin time-international normalized ratio.

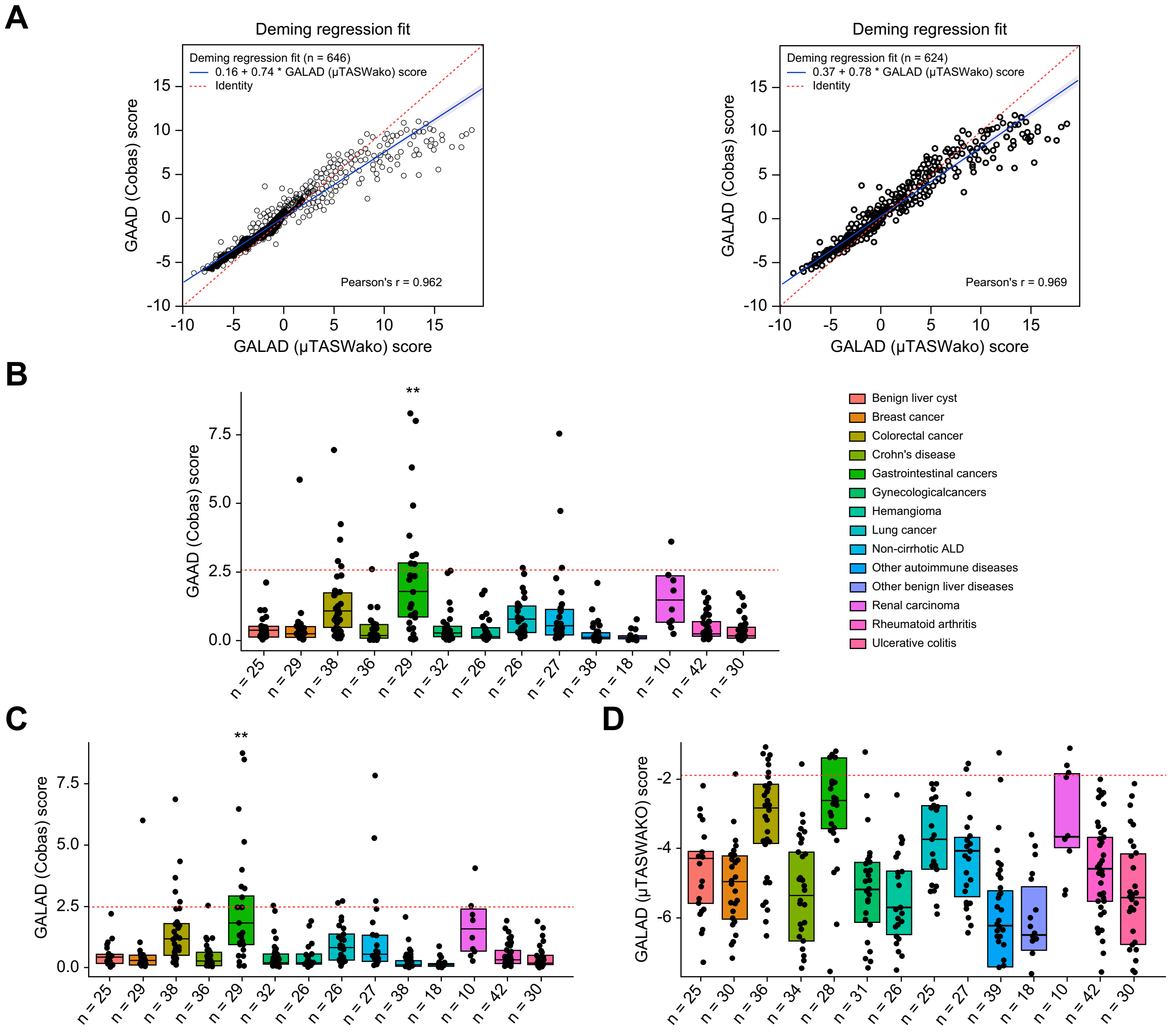

Fig. 3. Weighted Deming regression fit and specificty panel data in STOP-HCC-MCE. Weighted Deming regression fit of agreement between GAAD (Cobas) and GALAD (μTASWAKO), GALAD (Cobas) and GALAD (μTASWAKO) in STOP-HCC-MCE (A), specificity panel data across disease groups for GAAD (Cobas) (B), GALAD (Cobas) (C), and GALAD (μTASWAKO) (D) in STOP-HCC-MCE. Levels of significance: ***p* <0.01. (*p* values were calculated using binomial tests, Bonferroni correction for every marker, and specificity values with the corresponding cut-offs for the markers: GAAD [2.57, 90%], GALAD [2.47, 90%], WAKO GALAD [−1.89, 90%].) ALD, alcoholic-related liver disease; GAAD, gender (biological sex), age, AFP, DCP (PIVKA-II); GALAD, gender (biological sex), age, AFP-L3, AFP, DCP (PIVKA-II).

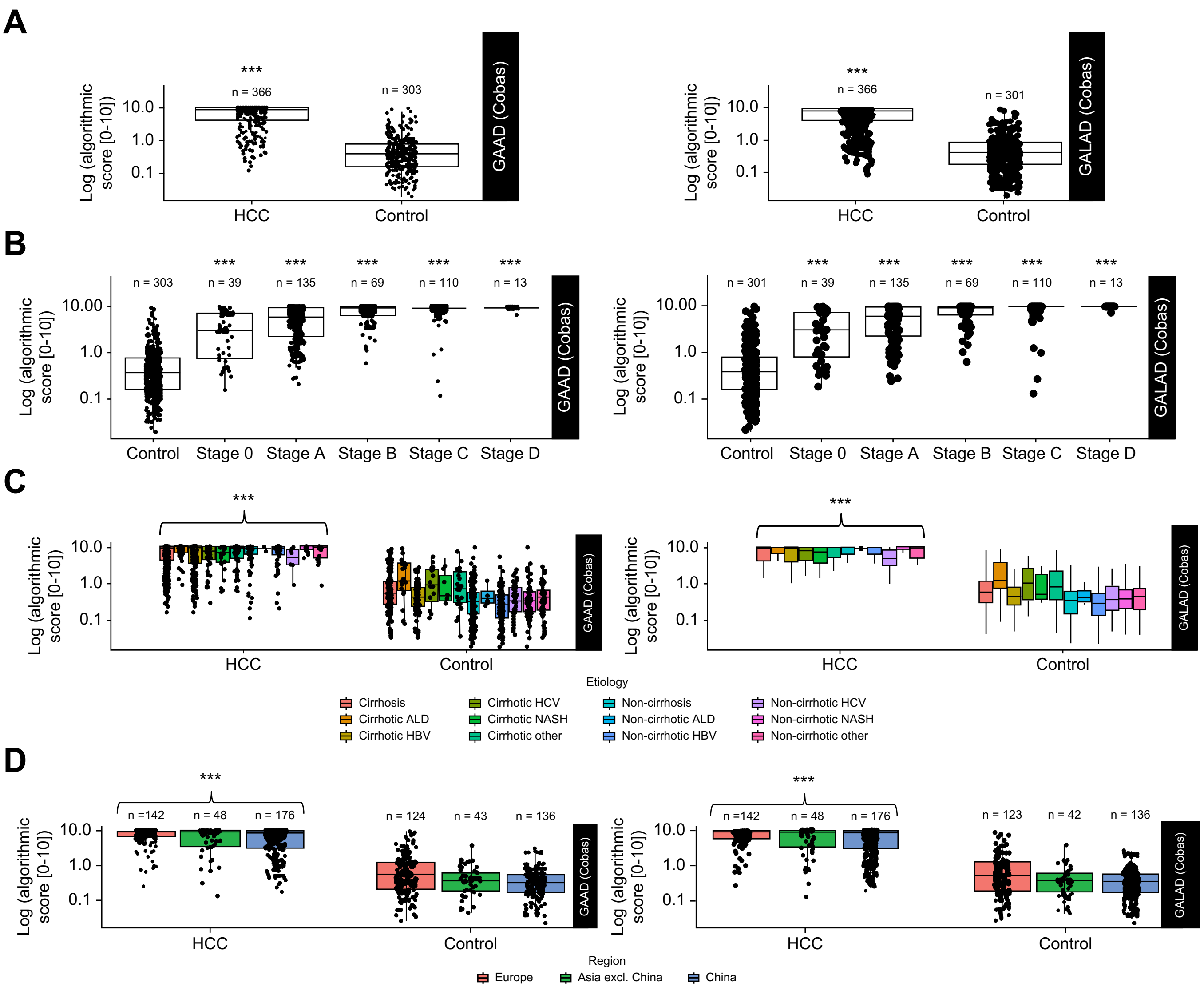

Fig. 4. Distribution of GAAD (Cobas) and GALAD (Cobas) scores. Distribution by HCC and CLD controls (A), BCLC (B), etiology (C), and geographical region (D) in STOP-HCC-MCE. Levels of significance: ****p* <0.001 compared with control (Welch's *t* test). ALD, alcoholic-related liver disease; BCLC, Barcelona Clinic Liver Cancer; CLD, chronic liver disease; CLD, chronic liver disease; GAAD, gender (biological sex), age, AFP, DCP (PIVKA-II); GALAD, gender (biological sex), age, AFP-L3, AFP, DCP (PIVKA-II); HBV, hepatitis B virus; HCC, hepatocellular carcinoma; HCV, hepatitis C virus; NASH, non-alcoholic steatohepatitis.

[Cobas]) specificity (Fig. 5D). When using two cut-offs for GALAD (µTASWAKO), respectively, (–0.63 and –1.89 [comparable to GAAD (Cobas) at 90% specificity]), the sensitivity was 56.3% and 72.4% in early-stage, 86.5% and 92.2% in late-stage, and 72.1% and 82.8% in all-stage HCC; specificity was 98.3% and 89.1% in CLD controls (Fig. 5D). GAAD (Cobas) was non-inferior (by a 1% margin) compared with GALAD (Cobas) and GALAD (µTASWAKO) across HCC stages (*p* <0.001), except for GALAD (µTASWAKO) in early-stage HCC (*p* = 0.02). A contingency table (Table S3) demonstrated that GAAD (Cobas) could detect more early-stage HCC cases, compared with GALAD (Cobas) and GALAD (µTASWAKO) (three and 17 cases, respectively).

The performance of GAAD (Cobas), GALAD (Cobas), and GALAD (µTASWAKO) was superior to individual biomarkers (AFP, AFP-L3, and PIVKA-II) alone across HCC stages (Fig. 5A–D). For individual biomarkers and different algorithms, clinical performance at predefined cut-offs are presented in Table S4, and cut-offs at different specified sensitivity and specificity in Tables S5 and S6, respectively.

In early-, late-, and all-stage HCC with cirrhotic etiology, AUCs were comparable at 85.3–89.5%, 97.0–98.2%, and 92.9–93.6% for GAAD (Cobas), and 85.7–89.8%, 96.9–98.2%, and 93.0–93.7% for GALAD (Cobas), respectively (Fig. 6A). AUCs for GAAD and GALAD (Cobas) were also similarly high (94.6–99.9%) across all HCC disease stages in subgroups without cirrhosis (Fig. 6A). When samples were split by region (i.e. Europe, Asia Pacific, China), AUCs for GAAD (Cobas) and GALAD (Cobas) remained similar, regardless of disease stage, ranging from 92.1% to 94.1% for early-, from 96.0% to 99.7% for late-, and from 94.9% to 96.0% for all-stage HCC (Fig. 6B).

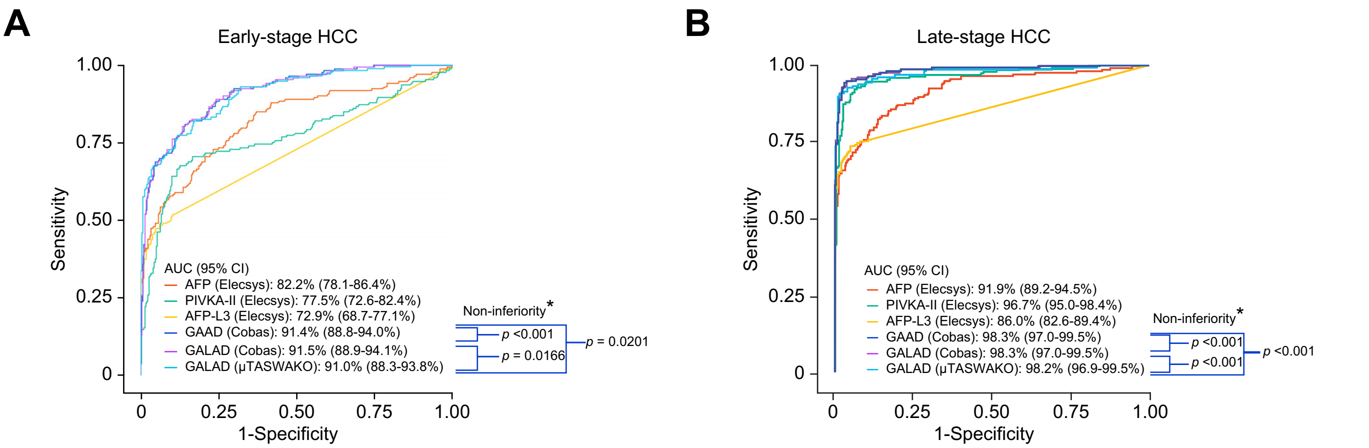

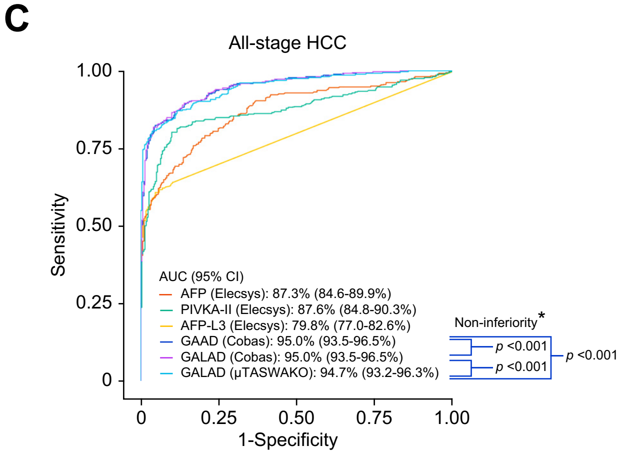

D

Assay/score (cut-off)	Sensitivity, % (95% CI) Early-stage HCC (BCLC 0/A) (n = 174)	Late-stage HCC (BCLC B-D) (n = 192)	All-stage HCC (n = 366)	Specificity, % (95% CI) CLD controls (n = 302)
AFP (Elecsys, 20 ng/ml)	41.4 (34.0-49.1)	65.1 (57.9-71.8)	53.8 (48.6-59.0)	98.0 (95.7-99.3)
PIVKA-II (Elecsys, 28.4 ng/ml)	60.9 (53.2-68.2)	94.3 (90.0-97.1)	78.4 (73.8-82.5)	90.4 (86.5-93.5)
AFP-L3 (Elecsys, 2.3 ng/ml)	40.8 (33.4-48.5)	68.2 (61.1-74.7)	55.2 (49.9-60.4)	98.3 (96.2-99.5)
GAAD (Cobas, 2.57)	70.1 (62.7-76.8)	94.8 (90.6-97.5)	83.1 (78.8-86.8)	93.7 (90.4-96.2)
GALAD (Cobas, 2.47)	70.1 (62.7-76.8)	95.3 (91.3-97.8)	83.3 (79.1-87.0)	93.0 (89.5-95.6)
GALAD (µTASWAKO, -0.63)	56.3 (48.6-63.8)	86.5 (80.8-91.0)	72.1 (67.2-76.7)	98.3 (96.2-99.5)
GALAD (µTASWAKO, -1.89)[a]	72.4 (65.1-78.9)	92.2 (87.4-95.6)	82.8 (78.5-86.5)	89.1 (85.0-92.4)

Fig. 5. Clinical performance of individual Elecsys assays, AFP, PIVKA-II, and AFP-L3 and algorithmic scores, GAAD (Cobas), GALAD (Cobas), and GALAD (µTASWAKO) in STOP-HCC-MCE. Clinical performance in differentiating early-stage (A); late-stage (B); and all-stage HCC (C) from CLD controls. Levels of significance: early-stage HCC: GAAD (Cobas) *vs.* GALAD (Cobas) $p < 0.001$; GAAD (Cobas) *vs.* GALAD (µTASWAKO) p = 0.0202; GALAD (Cobas) *vs.* GALAD (µTASWAKO) p = 0.0166; late- and all-stage HCC: $p < 0.001$ (p values comparing non-inferiority of AUCs were calculated using the H0 hypothesis AUC (Test 1) < AUC (Test 2) - 0.01 with a studentized bootstrap approach using N = 1,000 bootstrap replicates.) Sensitivities and specificities are shown in table (D). [a]Cut-off value corresponds to matching GAAD (Cobas) specificity of 90%. *AUC is not significantly worse by 1%. AFP, alpha-fetoprotein; AFP-L3, *Lens culinaris* agglutinin-reactive AFP; AUC, area under the curve; BCLC, Barcelona Clinic Liver Cancer; CLD, chronic liver disease; GAAD, gender (biological sex), age, AFP, DCP (PIVKA-II); GALAD, gender (biological sex), age, AFP-L3, AFP, DCP (PIVKA-II); HCC, hepatocellular carcinoma; PIVKA-II, protein induced by vitamin K absence or antagonist-II.

Discussion

Our study demonstrated for the first time that the GAAD (Cobas), GALAD (Cobas), and GALAD (µTASWAKO) algorithms performed equivalently well in differentiating HCC cases from CLD controls, irrespective of disease stage, etiology, or region. These findings suggest that GAAD may be a useful tool in identifying patients across etiologies and geographical regions who may benefit from more rigorous screening or liver transplantation. In the clinical validation study (STOP-HCC-MCE), GAAD (Cobas; cut-off 2.57), GALAD (Cobas; cut-off 2.47), and GALAD (µTASWAKO; cut-off −1.89) were similar in their ability to differentiate early- (AUC: 91.0–91.5%), and all-stage HCC (94.7–95.0%) from benign CLD. Similar to GALAD (Cobas), GAAD (Cobas) also achieved high specificity of 93.7%, and low false positive rates and high true negative detection rates, regardless of HCC stage. Performance data of the GALAD algorithms in our study were reflective of a previously published large meta-analysis including over 19,000 patients; for example the sensitivity, specificity, and AUC of GALAD were 73% (95% CI: 66–79%), 87% (95% CI: 81–91%), and 86% (95% CI: 82–88%), respectively, for the diagnosis of early-stage HCC.[12]

When considering the similar performance of GAAD and GALAD in our study with changing disease etiologies, treatment paradigms, and other phase II biomarker studies, questions arise regarding the utility of AFP-L3 in HCC surveillance. Although AFP-L3 alone is effective in detecting early-stage HCC with AFP+ tumors, with 9–12 months of lead time, compared with imaging techniques,[26] AFP-L3 is usually not detected when AFP levels are <20 ng/ml. This was reflected in STOP-HCC-MCE; of 926 patients with AFP <20 ng/ml, only 10.9% of patients had detectable AFP-L3 (data not shown). Therefore, AFP-L3 may not be relevant for HCC diagnosis in individuals with AFP-negative tumors.[27] Although global viral hepatitis-related HCC incidence has plateaued, HBV remains the most common cause of HCC in Asia,[28,29] as reflected in our clinical validation study (79.4% at Asian sites had HBV, and around 50% of these patients were treated with antiviral therapies, likely leading to normalized AFP levels).[20] Lower AFP levels at HCC diagnosis have also been reported across other etiologies, including MASLD,[30,31] in which PIVKA-II demonstrated better HCC diagnostic ability.[31] As non-viral etiologies are increasing worldwide, it will be beneficial to combine

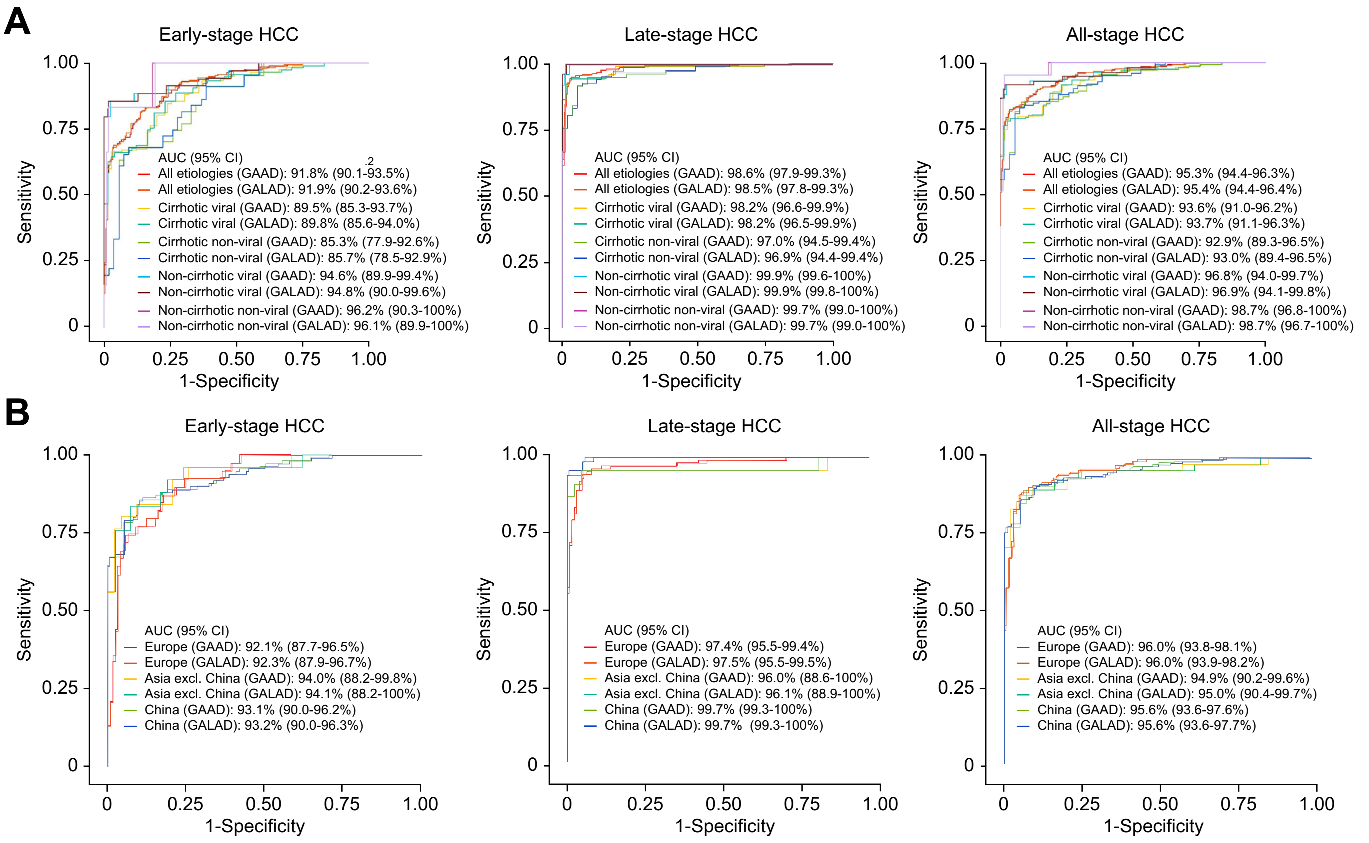

Fig. 6. ROC curves of GAAD (Cobas), GALAD (Cobas) scores for discriminating between HCC and CLD controls in STOP-HCC-MCE. Overall and by etiology group in early-stage, late-stage and all-stage HCC (A); and by geographical region in early-stage, late-stage and all-stage HCC (B). AUC, area under the curve; CLD, chronic liver disease; GAAD, gender (biological sex), age, AFP, DCP (PIVKA-II); GALAD, gender (biological sex), age, AFP-L3, AFP, DCP (PIVKA-II); HCC, hepatocellular carcinoma.

biomarkers, along with demographic characteristics, to improve diagnostic performance, as observed here with the GAAD algorithm—potentially useful for surveillance programs in all at-risk etiologies.

Additionally, biomarker combinations of PIVKA-II and AFP showed significantly higher clinical performance than AFP, PIVKA-II and AFP-L3 combined (AUCs 0.753 and 0.690, respectively; $p = 0.001$),[32] further signifying that AFP-L3 may have a negligible role in HCC detection. Similarly, the prospective ESCALON study revealed that AFP-L3 contributed minimally to early-stage HCC detection in two large multicenter cohorts from Latin America and Europe.[33] The value of AFP-L3 in algorithms that combine demographic characteristics and serum biomarkers is unclear. Previously, a phase III biomarker study found that, for HCC detection, the GALAD algorithm showed an improvement in sensitivity but with a similar AUC compared with AFP-L3 alone, suggesting that AFP-L3 contributed to GALAD performance.[16] Whereas, in our phase II studies, both the GAAD and GALAD (Cobas) algorithms were more sensitive than AFP-L3 in detecting early-stage HCC (70.1% [95% CI: 62.7–76.8%] for both algorithms and 40.8% [95% CI: 33.4–48.5%] for AFP-L3), had higher AUCs (91.4% *vs.* 91.5% *vs.* 72.9%) for detecting early-stage HCC and had higher overall true positive rates (25.6% *vs.* 25.7% *vs.* 14.9%). Although GALAD (Cobas) showed slightly higher false positivity rates (GAAD: 6.27% *vs.* GALAD: 6.98%), GAAD and GALAD (Cobas) demonstrated similar clinical performance in our study overall, suggesting that AFP-L3 has little influence on clinical performance.

The GAAD algorithm may also reduce costs and resource burden on the healthcare system. For example, in China, the cost of an AFP-L3 test was ~27.7 USD compared with ~6.2 USD for an AFP test, and ~18.5 USD for a PIVKA-II test; in a simulated cohort of 5,000 patients with HBV, at a cost-effectiveness threshold of three times China's GDP per capita, GAAD + USG was the most cost-effective screening strategy for HCC compared with USG and biomarkers alone.[17,21,34] Additionally, in separate simulated cohorts of 100,000 patients in the UK, cost-utility analysis found that GAAD was a cost-effective surveillance strategy compared with USG alone and USG + AFP.[35]

In the specificity panel analysis, of the conditions assessed, relatively high single biomarker concentrations (validated with μTASWAKO assays), and GAAD and GALAD scores, were observed for patients with gastrointestinal cancer. Patients with cholangiocarcinoma and pancreatic cancer were excluded from the analysis owing to high PIVKA-II expression levels. The small sample size was a limitation; however, previous studies also reported high PIVKA-II expression levels in patients with certain gastrointestinal, cholangiocarcinoma, and pancreatic cancers potentially attributable to underlying conditions (e.g. cholestatic disease, cholangitis, biliary stenosis, or bile duct stones) or the administration of antibiotics containing N-methyl-thiotetrazole during treatment.[36,37] These findings

indicate that high GAAD and GALAD scores should be used in conjunction with other clinical data, such as imaging, patient symptoms, and other laboratory tests.

Given the global plateau in viral hepatitis-related HCC incidence, our study was limited by the low number of patients with non-viral HCC etiology. This was because of the high proportion of Asian patients in our study, where viral etiologies are prevalent, therefore, additional analyses in larger non-viral cohorts may be beneficial. Another limitation was that patient evaluations were conducted in specialized care centers. Further studies should evaluate the clinical performance of GAAD and GALAD in primary care and non-specialist settings where serum assays can also be implemented for a better understanding of real-world performance.

Ultimately, the aim of this study was to validate and provide evidence to support the introduction of the GAAD algorithm as a potential alternative method to GALAD for the detection of HCC, which may result in optimized healthcare spend and improved patient outcomes. In summary, GAAD exhibits high sensitivity and low false positivity for the detection of early-stage HCC, performing favorably compared with individual surveillance biomarkers across disease etiologies and geographical regions. However, further validation through larger phase III/IV studies is required to assess the benefit-to-harm ratio of GAAD-based surveillance. Nonetheless, these findings demonstrate the potential of blood-based biomarker panels in addressing the significant unmet need for early detection of HCC.

Conclusions

The GAAD (Cobas) algorithm demonstrated good clinical performance and was as sensitive and specific as the GALAD (Cobas) and GALAD (μTASWAKO) algorithms in differentiating HCC and CLD controls, across all disease stages, etiologies, and regions. In participants with CLD undergoing guideline-directed HCC surveillance, the GAAD (Cobas) algorithm may be a time- and cost-efficient tool for early-stage HCC detection, with the potential to increase curative treatment opportunities and reduce mortality.

Affiliations

[1]*Hepatology Unit and Department of Infectious Diseases, Nanfang Hospital, Southern Medical University, Guangzhou China;* [2]*Division of Hepatology, Department of Medicine II, University of Leipzig Medical Center, Leipzig, Germany;* [3]*Department of Gastroenterology, Hepatology and Endocrinology, Medizinische Hochschule Hannover, Hannover, Germany (At the time of analysis);* [4]*Division of Gastroenterology and Hepatology, Toronto General Hospital, Toronto, Canada;* [5]*Medical Oncology, Princess Margaret Cancer Centre, Toronto, Canada;* [6]*Division of Gastroenterology and Hepatology Department of Medicine, Prince of Songkla University, Hat Yai, Thailand;* [7]*Department of Gastroenterology, Goethe Universitaet Frankfurt, Frankfurt, Germany;* [8]*Department of Medicine II, University Hospital, Ludwig Maximilian University of Munich, Munich, Germany;* [9]*Department of Gastroenterology and Hepatology, Kindai University, Osaka, Japan;* [10]*Department of Biomarker Services, Microcoat Biotechnologie GmbH, Bernried, Germany;* [11]*MVZ Labor Dr. Limbach & Kollegen, Heidelberg, Germany;* [12]*Department of Studies, Collaboration and Innovation Management, Labor Berlin Charité Vivantes Services GmbH, Berlin, Germany;* [13]*Department of Surgery, Universitätsmedizin Berlin, Chirurgische Klinik, Campus Charité Mitte and Campus Virchow-Klinikum, Berlin, Germany;* [14]*Global Study Management, Roche Diagnostics GmbH, Penzberg, Germany;* [15]*Clinical Algorithms & Biomarker Statistics, Roche Diagnostics GmbH, Penzberg, Germany;* [16]*Department of Internal Medicine, The Chinese University of Hong Kong, Hong Kong Special Administrative Region of China;* [17]*Clinical Development & Medical Affairs, Roche Diagnostics International AG, Rotkreuz, Switzerland*

Abbreviations

AFP, alpha-fetoprotein; AFP-L3, *Lens culinaris* agglutinin-reactive AFP; ALBI, albumin–bilirubin; ALD, alcoholic-related liver disease; ALT, alanine transaminase; AST, aspartate transferase; AUC, area under the curve; BCLC, Barcelona Clinic Liver Cancer; CLD, chronic liver disease; DCP, des-gamma carboxyprothrombin (PIVKA-II); FN, false negatives; FP, false positives; GAAD, gender (biological sex), age, AFP, DCP (PIVKA-II); GALAD, gender (biological sex), age, AFP-L3, AFP, DCP (PIVKA-II); HBV, hepatitis B virus; HCC, hepatocellular carcinoma; HCV, hepatitis C virus; ICF, International Classification of Functioning, Disability and Health; IQR, interquartile range; MASH, metabolic dysfunction-associated steatohepatitis; MASLD, metabolic dysfunction-associated steatotic liver disease; MELD, model for end-stage liver disease; NE, not evaluable; NPV, negative predictive value; PIVKA-II, protein induced by vitamin K absence or antagonist-II; PPV, positive predictive value; PS, performance status; PT-INR, prothrombin time-international normalized ratio; ROC, receiver operating characteristics; SAR, Special Administrative Region; TN, true negatives; TP, true positives; USG, ultrasonography.

Financial support

This study was funded by Roche Diagnostics GmbH (Penzberg, Germany).

Conflicts of interest

JH reports speaker's bureau participation for Glaxo-Smith-Kline, Gilead Sciences, Roche Diagnostics, grant/research support from Gilead Sciences, BMS, advisory committee or review panel for Aligos, Assembly, Glaxo-Smith-Kline, Gilead Sciences, Johnson Pharmaceutica, and Roche. TB reports consultancy fees from Bayer, Eisai, Ipsen, Merck Sharp & Dome/Merck, Sirtex, and Roche. AV reports consultancy fees from AstraZeneca, Amgen, BeiGene, Böhringer Mannheim, BMS, BTG, Daichi-Sankyo, EISAI, Incyte, Ipsen, MSD, PierreFabre, Roche, Servier, Sirtex, Tahio, Terumo. Speaker for AstraZeneca, Amgen, BeiGene, Böhringer Mannheim, BMS, BTG, Daichi-Sankyo, EISAI, GSK, Imaging Equipment Ltd (AAA), Incyte, Ipsen, Jiangsu Hengrui Medicines MSD, PierreFabre, Roche, Servier, Sirtex, Tahio, Terumo. Research funding from Servier, and Incyte. Commercial medical education provider for Onclive, Oncowissen.de. TP reports speaker's bureau participation for Bristol-Myers Squibb, Gilead Science, Bayer, Abbott, and Eisai, and MSD and research grant/contracts from Gilead Science, Roche Diagnostics, Jannsen Fibrogen, and VIR. JT reports consultancy fees for Amgen, Bayer Healthcare, Bristol-Myers Squibb, Eisai, Ipsen, Merck Serono, Merck Sharp & Dome, Lilly ImClone, and Roche. ENDeT has served as a paid consultant for AstraZeneca, Bayer, BMS, EISAI, Eli Lilly & Co, MSD, Mallinckrodt, Omega, Pfizer, Ipsen, Terumo and Roche and is currently employed by Boehringer-Ingelheim. He has received reimbursement of meeting attendance fees and travel expenses from Arqule, AstraZeneca, BMS, Bayer, Celsion, and Roche, and lecture honoraria from BMS and Falk. He has received third-party funding for scientific research from Arqule, AstraZeneca, BMS, Bayer, Eli Lilly, Ipsen, and Roche. MK reports speaking and teaching for Eisai, Bayer, Merck Sharp & Dome, Bristol-Myers Squibb, Eli Lilly & Co, and EA Pharma, and grant/research support from Gilead Sciences, Taiho, Sumitomo Dainippon Pharma, Takeda, Otsuka, EA Pharma, AbbVie, Eisai, Ono, and advisory committee or review panel for Eisai, Ono, MSD, Bristol-Myers Squibb, and Roche. KMal is an employee of Microcoat Biotechnologie, contracted by Roche Diagnostics. KMad, KK, and AS are employees of Roche Diagnostics International AG. PF, JKH, and WS have no conflicts to declare. HLYC reports consultancy fees from Arbutus Biopharma, Gilead Sciences, Glaxo-Smith-Kline, Roche, Vir Biotechnology, Aligos Therapeutics, Vaccitech, and Virion Therapeutics, and speaker's bureau participation for Echosens, Gilead Sciences, Roche, and Viatris.

Please refer to the accompanying ICMJE disclosure forms for further details.

Authors' contributions

Conceptualization: AS, HLYC, TB. Funding acquisition: AS. Investigation: AS, AV, ENDeT, JH, JKH, JT, KMal, KMad, MK, TB, TP, WS. Methodology: AS, HLYC, KK, KMal, KMad, MK, PF. Supervision: AS, ENDeT, HLYC, JH, PF, TB, TP. Validation: AS, HLYC, KK, KMal, KMad, MK. Visualization: AS, KK. Formal analysis: HLYC, KMal, KMad, TB. Project administration: JKH. Data curation: AV, JT, KK, KMal, KMad, MK, PF. Software: KK. Resources: PF. Contributed to the writing and reviewing of the manuscript, and approval of the final manuscript: all authors.

Data availability statement

Requests concerning the data supporting the findings of this study can be directed to rotkreuz.datasharingrequests@roche.com for consideration.

Acknowledgements

The authors would like to thank the participants and their families for their contribution to the study. We also thank Tawesak Tanwandee, Satawat Thongsawat, Wattana Sukeepaisarnjaroen; Juan Ignacio Esteban, Marta Bes, Bruno Köhler, Magdalena Swiatek-de Lange, and David Morgenstern for their contributions to the algorithm development part of the study.

Writing assistance

Editorial support for this manuscript, under the direction of the authors, was provided by Carolyn Bowler, PhD, CMPP^{TM}, Estelle Challinor, MSc, BSc, and Jade Drummond, BSc, of inScience Communications, Springer Healthcare Ltd, UK, and was funded by Roche Diagnostics International AG (Rotkreuz, Switzerland).

Declarations

GAAD is a CE-marked digital tool for aid in diagnosis of early-stage and all-stage HCC. ELECSYS and COBAS are trademarks of Roche. All other product names and trademarks are the property of their respective owners.

Supplementary data

Supplementary data to this article can be found online at https://doi.org/10.1016/j.jhepr.2024.101263.

References

Author names in bold designate shared co-first authorship

[1] Omata M, Cheng AL, Kokudo N, et al. Asia-Pacific clinical practice guidelines on the management of hepatocellular carcinoma: a 2017 update. Hepatol Int 2017;11:317–370.
[2] Llovet JM, Kelley RK, Villanueva A, et al. Hepatocellular carcinoma. Nat Rev Dis Primers 2021;7:6.
[3] Vogel A, Cervantes A, Chau I, et al. Hepatocellular carcinoma: ESMO Clinical Practice Guidelines for diagnosis, treatment and follow-up. Ann Oncol 2018;29(Suppl 4):238–255.
[4] Singal AG, Llovet JM, Yarchoan M, et al. AASLD Practice Guidance on prevention, diagnosis, and treatment of hepatocellular carcinoma. Hepatology 2023;78:1922–1965.
[5] European Association for the Study of the Liver. EASL clinical practice guidelines: management of hepatocellular carcinoma. J Hepatol 2018;69:182–236.
[6] Shiina S, Gani RA, Yokosuka O, et al. APASL practical recommendations for the management of hepatocellular carcinoma in the era of COVID-19. Hepatol Int 2020;14:920–929.
[7] Singal A, Volk ML, Waljee A, et al. Meta-analysis: surveillance with ultrasound for early-stage hepatocellular carcinoma in patients with cirrhosis. Aliment Pharmacol Ther 2009;30:37–47.
[8] Piratvisuth T, Tanwandee T, Thongsawat S, et al. Multimarker panels for detection of early stage hepatocellular carcinoma: a prospective, multicenter, case-control study. Hepatol Commun 2022;6:679–691.
[9] Kim DY, Toan BN, Tan CK, et al. Utility of combining PIVKA-II and AFP in the surveillance and monitoring of hepatocellular carcinoma in the Asia-Pacific region. Clin Mol Hepatol 2023;29:277–292.
[10] Kudo M. Early hepatocellular carcinoma: definition and diagnosis. Liver Cancer 2013;2:69–72.
[11] Wang T, Zhang KH. New blood biomarkers for the diagnosis of AFP-negative hepatocellular carcinoma. Front Oncol 2020;10:1316.
[12] Guan MC, Zhang SY, Ding Q, et al. The performance of GALAD score for diagnosing hepatocellular carcinoma in patients with chronic liver diseases: a systematic review and meta-analysis. J Clin Med 2023;12:949.
[13] Berhane S, Toyoda H, Tada T, et al. Role of the GALAD and BALAD-2 serologic models in diagnosis of hepatocellular carcinoma and prediction of survival in patients. Clin Gastroenterol Hepatol 2016;14:875–886.e6.
[14] Johnson PJ, Pirrie SJ, Cox TF, et al. The detection of hepatocellular carcinoma using a prospectively developed and validated model based on serological biomarkers. Cancer Epidemiol Biomarkers Prev 2014;23:144–153.
[15] Best J, Bechmann LP, Sowa JP, et al. GALAD score detects early hepatocellular carcinoma in an international cohort of patients with nonalcoholic steatohepatitis. Clin Gastroenterol Hepatol 2020;18:728–735.e4.
[16] Tayob N, Kanwal F, Alsarraj A, et al. The performance of AFP, AFP-3, DCP as biomarkers for detection of hepatocellular carcinoma (HCC): a phase 3 biomarker study in the United States. Clin Gastroenterol Hepatol 2023;21:415–423.e4.
[17] Wen C, Nan Y, Osvoldo U, et al. Cost-effectiveness analysis of GAAD algorithm on hepatocellular carcinoma screening in patients with chronic hepatitis B in China. Presented at ISPOR Europe, 6–9 Nov 2022; Vienna, Austria. 2022.
[18] Nagaoki Y, Hyogo H, Ando Y, et al. Increasing incidence of non-HBV- and non-HCV-related hepatocellular carcinoma: single-institution 20-year study. BMC Gastroenterol 2021;21:306.
[19] Ioannou GN. Epidemiology and risk-stratification of NAFLD-associated HCC. J Hepatol 2021;75:1476–1484.
[20] **Yang JD**, **Dai J**, Singal AG, et al. Improved performance of serum alpha-fetoprotein for hepatocellular carcinoma diagnosis in HCV cirrhosis with normal alanine transaminase. Cancer Epidemiol Biomarkers Prev 2017;26:1085–1092.
[21] **Liu S**, **Sun L**, Yao L, et al. Diagnostic performance of AFP, AFP-L3, or PIVKA-II for hepatitis C virus-associated hepatocellular carcinoma: a multicenter analysis. J Clin Med 2022;11:5075.
[22] **Piratvisuth T**, **Hou J**, Tawandee T, et al. Development & clinical validation of a novel algorithmic score (GAAD) for detecting hepatocellular carcinoma in prospective cohort studies. Hepatol Commun 2023;7:e0317.
[23] Kudo M, Kawamura Y, Hasegawa K, et al. Management of hepatocellular carcinoma in Japan: JSH consensus statements and recommendations 2021 update. Liver Cancer 2021;10:181–223.
[24] Hyndman R, Fan Y. Sample quantiles in statistical packages. Am Stat 1996;50:361–365.
[25] Clopper CJ, Pearson ES. The use of confidence or fiducial limits illustrated in the case of the binomial. Biometrika 1934;26:404–413.
[26] Li D, Mallory T, Satomura S. AFP-L3: a new generation of tumor marker for hepatocellular carcinoma. Clin Chim Acta 2001;313:15–19.
[27] Tsuchiya N, Sawada Y, Endo I, et al. Biomarkers for the early diagnosis of hepatocellular carcinoma. World J Gastroenterol 2015;21:10573–10583.
[28] Jafri W, Kamran M. Hepatocellular carcinoma in Asia: a challenging situation. Euroasian J Hepatogastroenterol 2019;9:27–33.
[29] Zhang CH, Cheng Y, Zhang S, et al. Changing epidemiology of hepatocellular carcinoma in Asia. Liver Int 2022;42:2029–2041.
[30] Than NN, Ghazanfar A, Hodson J, et al. Comparing clinical presentations, treatments and outcomes of hepatocellular carcinoma due to hepatitis C and non-alcoholic fatty liver disease. QJM 2016;110:73–81.
[31] Ueno M, Takeda H, Takai A, et al. Risk factors and diagnostic biomarkers for nonalcoholic fatty liver disease-associated hepatocellular carcinoma: current evidence and future perspectives. World J Gastroenterol 2022;28:3410–3421.
[32] Park SJ, Jang JY, Jeong SW, et al. Usefulness of AFP, AFP-L3, and PIVKA-II, and their combinations in diagnosing hepatocellular carcinoma. Medicine 2017;96:e5811.
[33] **Beudeker BJB**, **Fu S**, Balderramo D, et al. Validation and optimization of AFP-based biomarker panels for early HCC detection in Latin America and Europe. Hepatol Commun 2023;7:e0264.
[34] Nan Y, Garay OU, Lu X, et al. Early-stage hepatocellular carcinoma screening in patients with chronic hepatitis B in China: a cost-effectiveness analysis. J Comp Eff Res 2024;13:e230146.
[35] Garay UAL, Bird TG, Walkley R, et al. Cost-utility analysis of Elecsys GAAD algorithm versus ultrasound plus A-fetoprotein (AFP) for HCC surveillance in patients with compensated liver cirrhosis in the United Kingdom (UK) [abstract EE37]. Value Health 2022;25(12 Suppl):S60.
[36] Ge C, Luo M, Guo K, et al. Role of PIVKA-II in screening for malignancies at a hepatobiliary and pancreatic disease center: a large-scale real-world study. iLIVER 2022;1:209–216.
[37] Kanazumi N, Takeda S, Inoue S, et al. PIVKA-II during perioperative period in patients with hepato-biliary-pancreatic diseases. Hepatogastroenterology 2000;47:1695–1699.

Keywords: Hepatocellular carcinoma; Surveillance; Algorithm; GAAD; GALAD.
Received 12 April 2024; received in revised form 22 October 2024; accepted 29 October 2024; Available online 8 November 2024

Journal of Hepatology, Volume *7*

Supplemental information

Comparative evaluation of multimarker algorithms for early-stage HCC detection in multicenter prospective studies

Jinlin Hou, Thomas Berg, Arndt Vogel, Teerha Piratvisuth, Jörg Trojan, Enrico N. De Toni, Masatoshi Kudo, Katarina Malinowsky, Peter Findeisen, Johannes Kolja Hegel, Wenzel Schöning, Kairat Madin, Konstantin Kroeniger, Henry Lik-Yuen Chan, and Ashish Sharma

Comparative evaluation of multimarker algorithms for early-stage HCC detection in multicenter prospective studies

Jinlin Hou, Thomas Berg, Arndt Vogel, Teerha Piratvisuth, Jörg Trojan, Enrico N. De Toni, Masatoshi Kudo, Katarina Malinowsky, Peter Findeisen, Johannes Kolja Hegel, Wenzel Schöning, Kairat Madin, Konstantin Kroeniger, Henry Lik-Yuen Chan, Ashish Sharma

Table of contents

Fig. S1 2

Fig. S2 5

Table S1 6

Table S2 8

Table S3 12

Table S4 14

Table S5 17

Table S6 21

Fig. S1. Clinical performance of GAAD (cobas), GALAD (cobas), and GALAD (µTASWAKO) algorithmic scores in STOP-HCC-ARP for differentiating between early-stage HCC (A), late-stage HCC (B), and all-stage (C) and disease controls by etiology.

A.

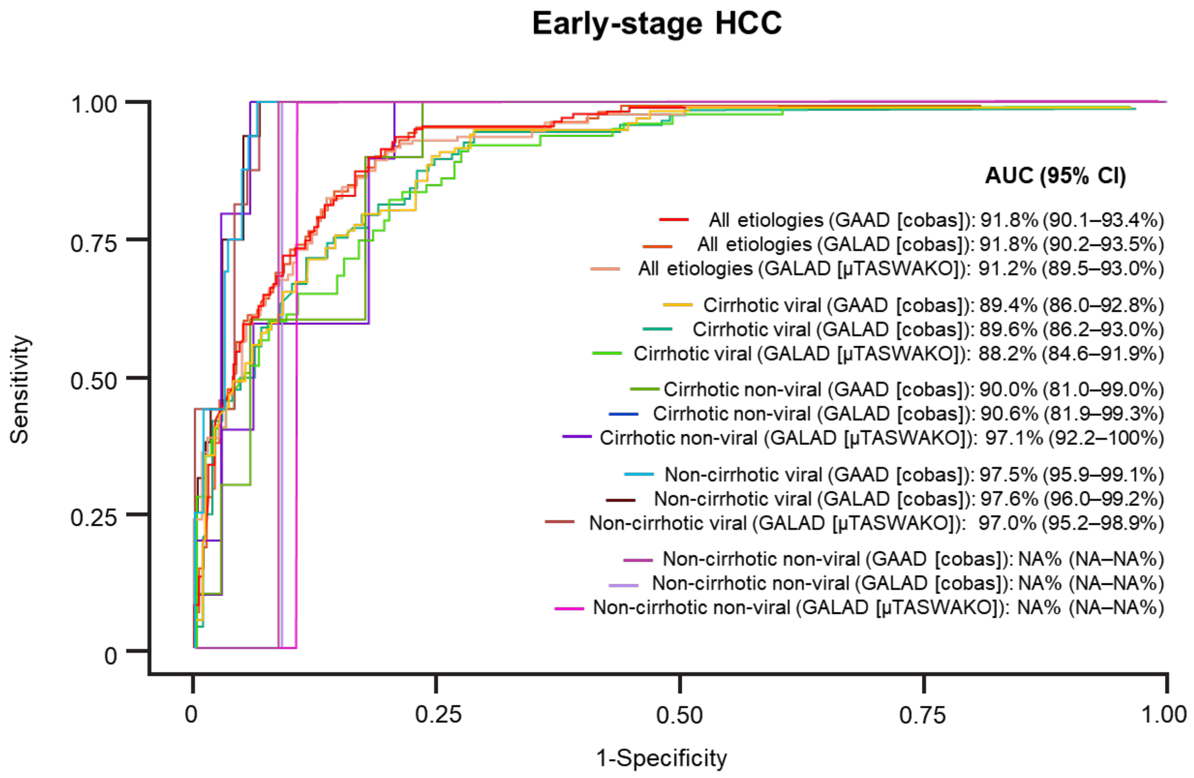

B.

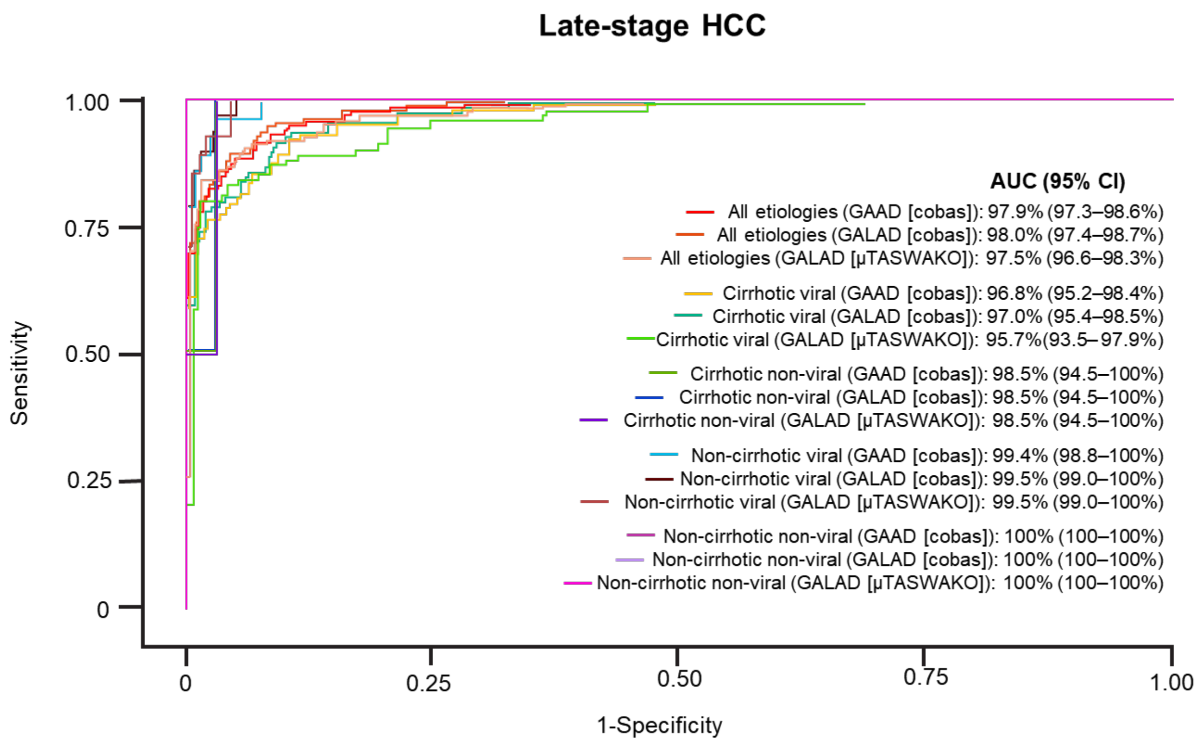

Late-stage HCC
Sensitivity
1.00
0.75
0.50
0.25
0
1-Specificity
0
0.25
0.50
0.75
1.00
AUC (95% CI)
All etiologies (GAAD [cobas]): 97.9% (97.3–98.6%)
All etiologies (GALAD [cobas]): 98.0% (97.4–98.7%)
All etiologies (GALAD [µTASWAKO]): 97.5% (96.6–98.3%)
Cirrhotic viral (GAAD [cobas]): 96.8% (95.2–98.4%)
Cirrhotic viral (GALAD [cobas]): 97.0% (95.4–98.5%)
Cirrhotic viral (GALAD [µTASWAKO]): 95.7%(93.5–97.9%)
Cirrhotic non-viral (GAAD [cobas]): 98.5% (94.5–100%)
Cirrhotic non-viral (GALAD [cobas]): 98.5% (94.5–100%)
Cirrhotic non-viral (GALAD [µTASWAKO]): 98.5% (94.5–100%)
Non-cirrhotic viral (GAAD [cobas]): 99.4% (98.8–100%)
Non-cirrhotic viral (GALAD [cobas]): 99.5% (99.0–100%)
Non-cirrhotic viral (GALAD [µTASWAKO]): 99.5% (99.0–100%)
Non-cirrhotic non-viral (GAAD [cobas]): 100% (100–100%)
Non-cirrhotic non-viral (GALAD [cobas]): 100% (100–100%)
Non-cirrhotic non-viral (GALAD [µTASWAKO]): 100% (100–100%)

C.

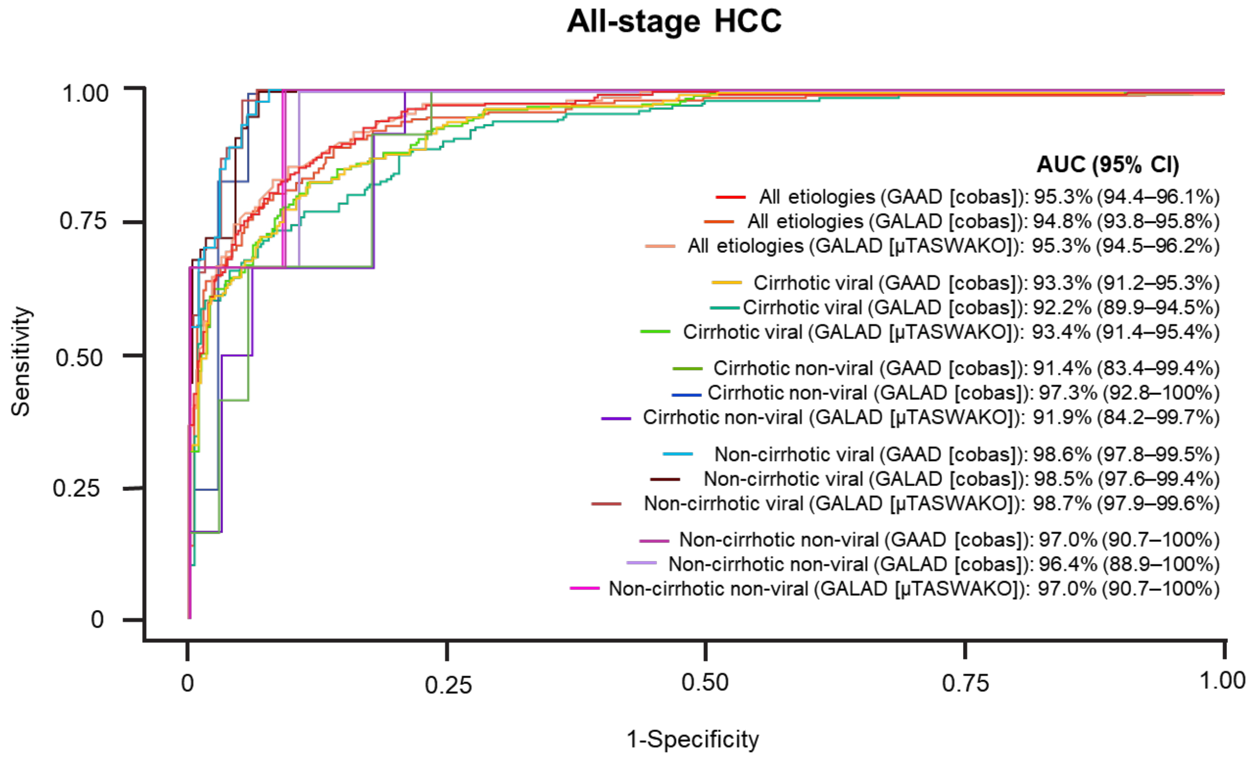

HCC, hepatocellular carcinoma.

Fig. S2. Venn diagram to illustrate the number of early-, late-, and, all-stage HCC cases, or controls detected by the single markers (Elecsys AFP, Elecsys AFP-L3, Elecsys PIVKA-II) using the predefined cutoffs (AFP 20 ng/mL; AFP-L3 2.3 ng/mL; PIVKA-II 28.4 ng/mL) in STOP-HCC-MCE.

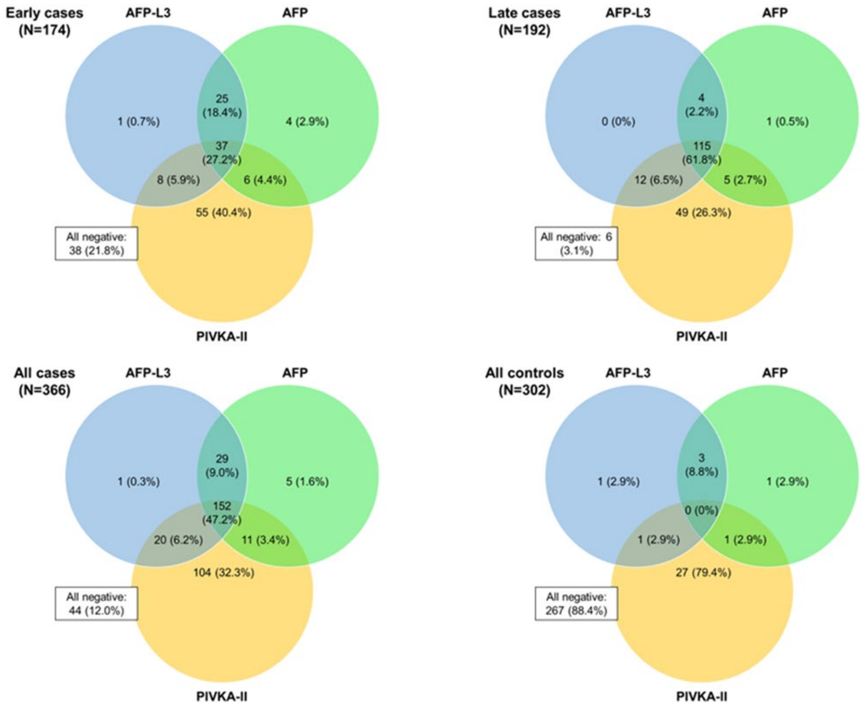

HCC, hepatocellular carcinoma.

Table S1. Participant demographics and clinical characteristics of the specificity panel in STOP-HCC-MCE.

	**Total (N=468)**
**Patient characteristics**	
**Age, years, mean (SD)**	53.9 (14.5)
**Sex, n (%)***	
Male	186 (39.7)
Female	282 (60.3)
**Race, n (%)***	
Asian	178 (38.0)
White	289 (61.8)

Black/African-American	1 (0.2)
Other	0
**Ongoing antiviral therapy, n (%)**	3 (0.6)
**Antibiotics, n (%)**	22 (4.7)

SD, standard deviation.

Table S2. Participant demographics and clinical characteristics in STOP-HCC-MCE by clinical sites.

	Hanover (N=88)	Leipzig (N=93)	Hat Yai (N=75)	Hong Kong* (N=109)	Guangzhou (N=203)	Frankfurt (N=46)	Berlin (N=1)	Munich (N=29)	Osaka (N=16)	Mainz (N=8)	Total (N=668)
**Patient characteristics**											
**Age, mean (SD)**	55.7 (14.8)	59.9 (12.2)	54.0 (10.4)	58.3 (10.3)	47.9 (11.0)	65.2 (12.0)	64.0 (NE)	58.2 (10.8)	67.9 (12.7)	51.6 (13.7)	55.2 (12.9)
**Sex, n (%)**											
Male	58 (65.9)	65 (69.9)	53 (70.7)	78 (71.6)	167 (82.3)	39 (84.8)	1 (100.0)	26 (89.7)	8 (50.0)	4 (50.0)	499 (74.7)
Female	30 (34.1)	28 (30.1)	22 (29.3)	31 (28.4)	36 (17.7)	7 (15.2)	0	3 (10.3)	8 (50.0)	4 (50.0)	169 (25.3)
**Race, n (%)**											
Asian	4 (4.6)	0	75 (100.0)	109 (100.0)	202 (99.5)	1 (2.2)	0	0	16 (100.0)	0	407 (60.9)
White	76 (86.4)	93 (100.0)	0	0	0	44 (95.7)	1 (100.0)	28 (96.6)	0	8 (100.0)	250 (37.4)

Black/ African American	2 (2.3)	0	0	0	0	1 (2.2)	0	1 (3.5)	0	0	4 (0.6)
Other	0	0	0	0	1 (0.5)	0	0	0	0	0	1 (0.2)
Missing	6 (6.8)	0	0	0	0	0	0	0	0	0	6 (0.9)
**Ongoing antiviral therapy, n (%)**	24 (27.3)	2 (2.2)	27 (36.0)	62 (56.9)	132 (65.0)	3 (6.5)	1 (100.0)	4 (13.8)	4 (25.0)	1 (12.5)	260 (38.9)
**Group, n (%)**											
HCC cases	39 (44.3)	41 (44.1)	40 (53.3)	56 (51.4)	120 (59.1)	38 (82.6)	1 (100.0)	21 (72.4)	8 (50.0)	2 (25.0)	366 (54.8)
Early-stage (BCLC 0/A)	7 (8.0)	9 (9.7)	19 (25.3)	43 (39.4)	67 (33.0)	8 (17.4)	1 (100.0)	12 (41.4)	6 (37.5)	2 (25.0)	174 (26.0)

Late-stage (BCLC B–D)	32 (36.4)	32 (34.4)	21 (28.0)	13 (11.9)	53 (26.1)	30 (65.2)	0	9 (31.0)	2 (12.5)	0	192 (28.7)
CLD controls	49 (55.7)	52 (55.9)	35 (46.7)	53 (48.6)	83 (40.9)	8 (17.4)	0	8 (27.6)	8 (50.0)	6 (75.0)	302 (45.2)
**Viral liver disease etiology, n (%)**	69 (78.4)	26 (28.0)	78 (104.0)	95 (87.2)	196 (96.6)	26 (55.3)	1 (100)	8 (27.6)	13 (81.3)	6 (75.0)	518 (669)
**Liver biochemistry & prognostic scores**											
AST, U/L, median (IQR)	36.5 (27.0–59.8)	38.6 (31.9–63.9)	44.0 (26.0–74.0)	33.0 (24.0–49.0)	28.0 (21.0–47.0)	46.5 (38.3–66.8)	37.0 (37.0–37.0)	60.0 (42.0–74.0)	30.5 (22.8–34.0)	31.0 (27.8–52.5)	36.0 (25.0–59.0)
ALT, U/L, median (IQR)	33.5 (25.0–48.3)	32.4 (23.4–46.2)	35.0 (23.5–56.0)	37.0 (23.0–53.0)	29.0 (20.0–43.0)	42.5 (31.3–58.5)	52.0 (52.0–52.0)	35.0 (27.0–48.0)	17.5 (13.0–22.5)	36.0 (21.3–47.0)	32.4 (22.0–49.1)

Serum albumin, g/L, median (IQR)	39.0 (35.0–43.0)	42.7 (38.1–47.2)	42.0 (35.5–45.5)	39.0 (36.0–40.0)	41.7 (36.7–45.7)	40.0 (37.0–44.0)	48.4 (48.4–48.4)	33.0 (30.0–40.0)	43.5 (41.0–44.3)	39.5 (37.5–40.3)	40.0 (36.0–44.3)
Serum bilirubin, μmol/L, median (IQR)	10.0 (6.0–15.0)	14.0 (8.8–23.6)	14.7 (8.7–23.0)	12.0 (9.0–18.0)	13.7 (10.6–19.3)	12.0 (10.3–15.4)	9.1 (9.1–9.1)	23.9 (15.4–46.2)	12.8 (10.3–17.1)	12.0 (9.8–12.8)	13.0 (9.2–20.0)

*Special Administrative Region of China.

Percentages may not add up to 100.0% due to rounding.

AST; aspartate aminotransferase; ALT; alanine aminotransferase; BCLC, Barcelona Clinic Liver Cancer; CLD, chronic liver disease; HCC, hepatocellular carcinoma; IQR, interquartile range; SD, standard deviation.

Table S3. Contingency tables for GAAD (cobas) compared with GALAD (cobas) and GALAD (µTASWAKO) in STOP-HCC-MCE.

HCC stage		GAAD (cobas)+	GAAD (cobas)-	Total
Early-stage	GALAD (cobas)+	111	0	111
	GALAD (cobas)-	3	51	54
	**Total**	114	51	165
All-stage	GALAD (cobas)+	272	1	273
	GALAD (cobas)-	3	60	63
	**Total**	275	61	336
Early-stage	GALAD (µTASWAKO)+	102	0	102
	GALAD (µTASWAKO)-	17	50	67
	**Total**	119	50	169
All-stage	GALAD (µTASWAKO)+	264	0	264
	GALAD (µTASWAKO)-	23	60	83
	**Total**	287	60	347

HCC, hepatocellular carcinoma.

GAAD+ (cobas) corresponds to patients with a GAAD (cobas) score ≥2.57, GAAD- (cobas) corresponds to patients with a GAAD (cobas) score <2.57.

GALAD+ (cobas) corresponds to patients with a GALAD (cobas) score ≥2.47, GALAD- (cobas) corresponds to patients with a GALAD (cobas) score <2.47.

GALAD+ (µTASWAKO) corresponds to patients with a GALAD (µTASWAKO) score ≥2.95, GALAD- (µTASWAKO) corresponds to patients with a GALAD (µTASWAKO) score <2.95.

Table S4. Clinical performance of Elecsys assays, AFP, PIVKA-II and AFP-L3 and algorithmic scores GAAD (cobas), GALAD (cobas), and GALAD (μTASWAKO) for the detection of early-stage (BCLC 0/A), late-stage (BCLC B–D), and all-stage HCC at the predefined cut-offs in STOP-HCC-MCE.

	AFP			PIVKA-II			AFP-L3			GAAD			GALAD			GALAD (μTASWAKO)		
	Early stage	Late stage	All stage	Early stage	Late stage	All stage	Early stage	Late stage	All stage	Early stage	Late stage	All stage	Early stage	Late stage	All stage	Early stage	Late stage	All stage
N (HCC/CLD control)	477 (174/ 303)	495 (192/ 303)	669 (366/ 303)	477 (174/ 303)	495 (192/ 303)	669 (366/ 303)	475 (174/ 301)	493 (192/ 301)	667 (366/ 301)	477 (174/ 303)	495 (192/ 303)	669 (366/ 303)	475 (174/ 301)	493 (192/ 301)	667 (366/ 301)	472 (173/ 299)	485 (186/ 299)	685 (359/ 299)
TP	72	125	197	106	181	287	71	131	202	122	182	304	122	183	305	99	168	267
TN	297	297	297	274	274	274	296	296	296	284	284	284	280	280	280	297	297	297
FP	6	6	6	29	29	29	5	5	5	19	19	19	21	21	21	2	2	2
FN	102	67	169	68	11	79	103	61	164	52	10	62	52	9	61	74	18	92

**Sensitivity (95% CI)**	**41.4 (34.0–49.1)**	**65.1 (57.9–71.8)**	**53.8 (48.6–59.0)**	**60.9 (53.2–68.2)**	**94.3 (90.0–97.1)**	**78.4 (73.8–82.5)**	**40.8 (33.4–48.5)**	**68.2 (61.1–74.7)**	**55.2 (49.9–60.4)**	**70.1 (62.7–76.8)**	**94.8 (90.6–97.5)**	**83.1 (78.8–86.8)**	**70.1 (62.7–76.8)**	**95.3 (91.3–97.8)**	**83.3 (79.1–87.0)**	**57.2 (49.5–64.7)**	**90.3 (85.1–94.2)**	**74.4 (69.5–78.8)**
**Specificity (95% CI)**	**98 (95.7–99.3)**	**98 (95.7–99.3)**	**98 (95.7–99.3)**	**90.4 (86.5–93.5)**	**90.4 (86.5–93.5)**	**90.4 (86.5–93.5)**	**98.3 (96.2–99.5)**	**98.3 (96.2–99.5)**	**98.3 (96.2–99.5)**	**93.7 (90.4–96.2)**	**93.7 (90.4–96.2)**	**93.7 (90.4–96.2)**	**93 (89.5–95.6)**	**93 (89.5–95.6)**	**93 (89.5–95.6)**	**99.3 (97.6–99.9)**	**99.3 (97.6–99.9)**	**99.3 (97.6–99.9)**
PPV 1% prev	17.4	24.9	21.5	6.0	9.1	7.6	19.9	29.3	25.1	10.1	13.2	11.8	9.2	12.1	10.8	46.4	57.7	52.9
PPV 2% prev	29.9	40.2	35.7	11.5	16.7	14.3	33.4	45.6	40.4	18.6	23.6	21.3	17.0	21.8	19.6	63.6	73.4	69.4
PPV 3% prev	39.3	50.4	45.7	16.4	23.3	20.2	43.2	56.0	50.7	25.7	31.9	29.1	23.7	29.7	27.0	72.6	80.7	77.5
PPV 4% prev	46.5	57.8	53.1	21	29.1	25.4	50.6	63.1	58.1	31.8	38.6	35.6	29.5	36.3	33.2	78.1	84.9	82.2
PPV 5% prev	52.4	63.4	58.9	25.1	34.1	30.1	56.4	68.4	63.6	37	44.3	41.1	34.6	41.8	38.6	81.8	87.7	85.4
NPV 1% prev	99.4	99.6	99.5	99.6	99.9	99.8	99.4	99.7	99.5	99.7	99.9	99.8	99.7	99.9	99.8	99.6	99.9	99.7
NPV 2% prev	98.8	99.3	99.0	99.1	99.9	99.5	98.8	99.3	99.1	99.4	99.9	99.6	99.3	99.9	99.6	99.1	99.8	99.5

NPV 3% prev	98.2	98.9	98.6	98.7	99.8	99.3	98.2	99.0	98.6	99.0	99.8	99.4	99.0	99.8	99.4	98.7	99.7	99.2
NPV 4% prev	97.6	98.5	98.1	98.2	99.7	99.0	97.6	98.7	98.1	98.7	99.8	99.3	98.7	99.8	99.3	98.2	99.6	98.9
NPV 5% prev	96.9	98.2	97.6	97.8	99.7	98.8	96.9	98.3	97.7	98.3	99.7	99.1	98.3	99.7	99.1	97.8	99.5	98.7

AFP, alpha-fetoprotein; AFP-L3, *Lens culinaris* agglutinin-reactive AFP; BCLC, Barcelona Clinic Liver Cancer; CI, confidence interval; CLD, chronic liver disease; DCP, des-gamma carboxyprothrombin (PIVKA-II); GAAD, gender (biological sex), age, AFP, DCP; GALAD, gender (biological sex), age, AFP-L3, AFP, DCP; FN, false negatives; FP, false positives; HCC, hepatocellular carcinoma; NPV, negative predictive value; PIVKA-II, protein induced by vitamin K absence or antagonist II; PPV, positive predictive value; TN, true negatives; TP, true positives.

Table S5. Cut-offs of Elecsys assays, AFP, PIVKA-II and AFP-L3 and algorithmic scores GAAD (cobas) and GALAD (cobas), at specified sensitivity in STOP-HCC-MCE.

**AFP**															
	**AFP**	**Sensitivity all-stage**	**Sensitivity early-stage (BCLC 0/A)**	**Sensitivity late-stage (BCLC B–D)**	**Specificity**	**PPV 1%**	**PPV 2%**	**PPV 3%**	**PPV 4%**	**PPV 5%**	**NPV 1%**	**NPV 2%**	**NPV 3%**	**NPV 4%**	**NPV 5%**
**Sensitivity 70%**	6.94	70.2 (65.2–74.9)	59.2 (51.5–66.6)	80.2 (73.9–85.6)	86.8 (82.5–90.4)	5.1	9.8	14.1	18.1	21.9	99.7	99.3	98.9	98.6	98.2
**Sensitivity 75%**	5.88	75.1 (70.4–79.5)	65.5 (57.9–72.5)	83.9 (77.9–88.8)	83.5 (78.8–87.5)	4.4	8.5	12.3	15.9	19.3	99.7	99.4	99.1	98.8	98.5
**Sensitivity 80%**	5.03	80.1 (75.6–84.0)	71.8 (64.5–78.4)	87.5 (82.0–91.8)	76.9 (71.7–81.5)	3.38	6.6	9.7	12.6	15.4	99.7	99.5	99.2	98.9	98.7
**Sensitivity 85%**	4.23	85.2 (81.2–88.7)	78.2 (71.3–84.1)	91.7 (86.8–95.2)	70.3 (64.8–75.4)	2.82	5.53	8.15	10.7	13.1	99.8	99.6	99.4	99.1	98.9
**Sensitivity 90%**	3.62	90.2 (86.6–93.0)	84.5 (78.2–89.5)	95.3 (91.3–97.8)	63.4 (57.7–68.8)	2.43	4.78	7.07	9.3	11.5	99.8	99.7	99.5	99.4	99.2
**Sensitivity 95%**	2.21	95.1 (92.3–97.1)	92.5 (87.6–96.0)	97.4 (94.0–99.1)	26.4 (21.5–31.7)	1.29	2.57	3.84	5.11	6.37	99.8	99.6	99.4	99.2	99.0
**PIVKA-II**															
	**PIVKA-II**	**Sensitivity all-stage**	**Sensitivity early-stage (BCLC 0/A)**	**Sensitivity late-stage (BCLC B–D)**	**Specificity**	**PPV 1%**	**PPV 2%**	**PPV 3%**	**PPV 4%**	**PPV 5%**	**NPV 1%**	**NPV 2%**	**NPV 3%**	**NPV 4%**	**NPV 5%**

**Sensitivity 70%**	47.2	69.9 (65.0–74.6)	46.6 (39.0–54.3)	91.1 (86.2–94.8)	93.7 (90.4–96.2)	10.1	18.5	25.6	31.7	37.0	99.7	99.3	99.0	98.7	98.3
**Sensitivity 75%**	33.8	75.1 (70.4–79.5)	55.7 (48.0–63.3)	92.7 (88.1–96.0)	92.4 (88.8–95.1)	9.09	16.8	23.4	29.2	34.3	99.7	99.5	99.2	98.9	98.6
**Sensitivity 80%**	27.8	80.1 (75.6–84.0)	63.8 (56.2–70.9)	94.8 (90.6–97.5)	90.1 (86.2–93.2)	7.55	14.2	20	25.2	29.9	99.8	99.6	99.3	99.1	98.8
**Sensitivity 85%**	19.3	85.2 (81.2–88.7)	73.0 (65.7–79.4)	96.4 (92.6–98.5)	71.6 (66.2–76.6)	2.94	5.78	8.5	11.1	13.6	99.8	99.6	99.4	99.1	98.9
**Sensitivity 90%**	16.7	89.3 (85.7–92.3)	79.3 (72.5–85.1)	98.4 (95.5–99.7)	45.9 (40.2–51.7)	1.64	3.26	4.86	6.44	7.99	99.8	99.5	99.3	99.0	98.8
**Sensitivity 95%**	14.0	95.4 (92.7–97.3)	90.2 (84.8–94.2)	100 (98.1–100)	19.5 (15.2–24.4)	1.18	2.36	3.53	4.7	5.87	99.8	99.5	99.3	99.0	98.8
**AFP-L3**															
	**AFP-L3**	**Sensitivity all-stage**	**Sensitivity early-stage (BCLC 0/A)**	**Sensitivity late-stage (BCLC B–D)**	**Specificity**	**PPV 1%**	**PPV 2%**	**PPV 3%**	**PPV 4%**	**PPV 5%**	**NPV 1%**	**NPV 2%**	**NPV 3%**	**NPV 4%**	**NPV 5%**
**Sensitivity 70%**	1.2	100 (99.0–100)	100 (97.9–100)	100 (98.1–100)	0 (0.0–1.22)	1	2	3	4	5	-	-	-	-	-
**Sensitivity 75%**	1.2	100 (99.0–100)	100 (97.9–100)	100 (98.1–100)	0 (0.0–1.22)	1	2	3	4	5	-	-	-	-	-
**Sensitivity 80%**	1.2	100 (99.0–100)	100 (97.9–100)	100 (98.1–100)	0 (0.0–1.22)	1	2	3	4	5	-	-	-	-	-
**Sensitivity 85%**	1.2	100 (99.0–100)	100 (97.9–100)	100 (98.1–100)	0 (0.0–1.22)	1	2	3	4	5	-	-	-	-	-

**Sensitivity 90%**	1.2	100 (99.0–100)	100 (97.9–100)	100 (98.1–100)	0 (0.0–1.22)	1	2	3	4	5	-	-	-	-	-
**Sensitivity 95%**	1.2	100 (99.0–100)	100 (97.9–100)	100 (98.1–100)	0 (0.0–1.22)	1	2	3	4	5	-	-	-	-	-
**GAAD**															
	**GAAD**	**Sensitivity all-stage**	**Sensitivity early-stage (BCLC 0/A)**	**Sensitivity late-stage (BCLC B–D)**	**Specificity**	**PPV 1%**	**PPV 2%**	**PPV 3%**	**PPV 4%**	**PPV 5%**	**NPV 1%**	**NPV 2%**	**NPV 3%**	**NPV 4%**	**NPV 5%**
**Sensitivity 70%**	6.12	70.2 (65.2–74.9)	50.6 (42.9–58.2)	88.0 (82.6–92.3)	98.7 (96.7–99.6)	34.9	52.1	62.2	68.9	73.7	99.7	99.4	99.1	98.8	98.4
**Sensitivity 75%**	4.46	75.1 (70.4–79.5)	57.5 (49.8–64.9)	91.1 (86.2–94.8)	98.0 (95.7–99.3)	27.7	43.6	54.0	61.3	66.6	99.7	99.5	99.2	99.0	98.7
**Sensitivity 80%**	3.45	80.1 (75.6–84.0)	63.8 (56.2–70.9)	94.8 (90.6–97.5)	96.0 (93.2–97.9)	17.0	29.2	38.5	45.7	51.5	99.8	99.6	99.4	99.1	98.9
**Sensitivity 85%**	1.88	85.0 (80.9–88.5)	73.0 (65.7–79.4)	95.8 (92.0–98.2)	89.4 (85.4–92.7)	7.52	14.1	19.9	25.1	29.7	99.8	99.7	99.5	99.3	99.1
**Sensitivity 90%**	1.15	89.9 (86.3–92.8)	81.6 (75.0–87.1)	97.4 (94.0–99.1)	84.8 (80.3–88.7)	5.64	10.8	15.5	19.8	23.8	99.9	99.8	99.6	99.5	99.4
**Sensitivity 95%**	0.683	94.8 (92.0–96.8)	90.8 (85.5–94.7)	98.4 (95.5–99.7)	71.6 (66.2–76.6)	3.26	6.38	9.36	12.2	15.0	99.9	99.9	99.8	99.7	99.6
**GALAD**															
	**GALAD**	**Sensitivity all-stage**	**Sensitivity early-stage (BCLC 0/A)**	**Sensitivity late-stage (BCLC B–D)**	**Specificity**	**PPV 1%**	**PPV 2%**	**PPV 3%**	**PPV 4%**	**PPV 5%**	**NPV 1%**	**NPV 2%**	**NPV 3%**	**NPV 4%**	**NPV 5%**

**Sensitivity 70%**	6.3	70.2 (65.2–74.9)	50.6 (42.9–58.2)	88.0 (82.6–92.3)	98.7 (96.6–99.6)	34.8	51.9	62.0	68.8	73.6	99.7	99.4	99.1	98.8	98.4
**Sensitivity 75%**	4.77	75.1 (70.4–79.5)	57.5 (49.8–64.9)	91.1 (86.2–94.8)	98.0 (95.7–99.3)	27.6	43.5	53.8	61.1	66.5	99.7	99.5	99.2	99.0	98.7
**Sensitivity 80%**	3.51	79.8 (75.3–83.8)	63.8 (56.2–70.9)	94.3 (90.0–97.1)	96.3 (93.6–98.2)	18.1	30.8	40.3	47.6	53.5	99.8	99.6	99.4	99.1	98.9
**Sensitivity 85%**	1.99	85.2 (81.2–88.7)	73.6 (66.4–79.9)	95.8 (92.0–98.2)	90.0 (86.1–93.2)	7.95	14.9	20.9	26.3	31.0	99.8	99.7	99.5	99.3	99.1
**Sensitivity 90%**	1.2	89.9 (86.3–92.8)	81.6 (75.0–87.1)	97.4 (94.0–99.1)	84.4 (79.8–88.3)	5.5	10.5	15.1	19.3	23.3	99.9	99.8	99.6	99.5	99.4
**Sensitivity 95%**	0.689	94.8 (92.0–96.8)	90.8 (85.5–94.7)	98.4 (95.5–99.7)	71.1 (65.6–76.2)	3.21	6.27	9.21	12.0	14.7	99.9	99.9	99.8	99.7	99.6

AFP, alpha-fetoprotein; AFP-L3, *Lens culinaris* agglutinin-reactive AFP; BCLC, Barcelona Clinic Liver Cancer; DCP, des-gamma carboxyprothrombin (PIVKA-II); GAAD, gender (biological sex), age, AFP, DCP; GALAD, gender (biological sex), age, AFP-L3, AFP, DCP; HCC, hepatocellular carcinoma; NPV, negative predictive value; PIVKA-II, protein induced by vitamin K absence or antagonist II; PPV, positive predictive value.

Table S6. Cut-offs of Elecsys assays, AFP, PIVKA-II and AFP-L3 and algorithmic scores GAAD (cobas) and GALAD (cobas) at specified specificity in STOP-HCC-MCE.

AFP															
	**AFP**	**Sensitivity all-stage**	**Sensitivity early-stage (BCLC 0/A)**	**Sensitivity late-stage (BCLC B–D)**	**Specificity**	**PPV 1%**	**PPV 2%**	**PPV 3%**	**PPV 4%**	**PPV 5%**	**NPV 1%**	**NPV 2%**	**NPV 3%**	**NPV 4%**	**NPV 5%**
**Specificity 70%**	4.17	85.5 (81.5–89.0)	78.2 (71.3–84.1)	92.2 (87.4–95.6)	70.0 (64.5–75.1)	2.8	5.5	8.1	10.6	13.0	99.8	99.6	99.4	99.1	98.9
**Specificity 75%**	4.69	81.4 (77.1–85.3)	73.6 (66.4–79.9)	88.5 (83.2–92.7)	74.9 (69.6–79.7)	3.2	6.2	9.1	11.9	14.6	99.8	99.5	99.2	99.0	98.7
**Specificity 80%**	5.33	78.4 (73.8–82.5)	69.0 (61.5–75.7)	87.0 (81.4–91.4)	79.5 (74.6–83.9)	3.7	7.3	10.6	13.8	16.8	99.7	99.4	99.2	98.9	98.6
**Specificity 85%**	6.09	73.0 (68.1–77.4)	61.5 (53.8–68.8)	83.3 (77.3–88.3)	84.8 (80.3–88.7)	4.6	8.9	12.9	16.7	20.2	99.7	99.4	99.0	98.7	98.3
**Specificity 90%**	8.51	67.2 (62.1–72.0)	58.0 (50.3–65.5)	75.5 (68.8–81.4)	89.8 (85.8–92.9)	6.2	11.8	16.9	21.5	25.7	99.6	99.3	98.9	98.5	98.1
**Specificity 95%**	12.8	60.4 (55.2–65.4)	49.4 (41.8–57.1)	70.3 (63.3–76.7)	94.7 (91.6–97.0)	10.4	18.9	26.1	32.3	37.6	99.6	99.2	98.7	98.3	97.8
**PIVKA-II**															
	**PIVKA-II**	**Sensitivity all-stage**	**Sensitivity early-stage (BCLC 0/A)**	**Sensitivity late-stage (BCLC B–D)**	**Specificity**	**PPV 1%**	**PPV 2%**	**PPV 3%**	**PPV 4%**	**PPV 5%**	**NPV 1%**	**NPV 2%**	**NPV 3%**	**NPV 4%**	**NPV 5%**

**Specificity 70%**	19.1	85.5 (81.5–89.0)	73.0 (65.7–79.4)	96.9 (93.3–98.8)	70.0 (64.5–75.1)	2.8	5.5	8.1	10.6	13.0	99.8	99.6	99.4	99.1	98.9
**Specificity 75%**	20.2	85.0 (80.9–88.5)	72.4 (65.1–78.9)	96.4 (92.6–98.5)	74.9 (69.6–79.7)	3.3	6.5	9.5	12.4	15.1	99.8	99.6	99.4	99.2	99.0
**Specificity 80%**	21.7	83.9 (79.7–87.5)	70.7 (63.3–77.3)	95.8 (92.0–98.2)	80.2 (75.3–84.5)	4.1	8.0	11.6	15.0	18.2	99.8	99.6	99.4	99.2	99.0
**Specificity 85%**	23.4	82.2 (77.9–86.0)	68.4 (60.9–75.2)	94.8 (90.6–97.5)	85.1 (80.6–89.0)	5.3	10.2	14.6	18.7	22.6	99.8	99.6	99.4	99.1	98.9
**Specificity 90%**	27.5	80.3 (75.9–84.3)	64.4 (56.8–71.5)	94.8 (90.6–97.5)	89.8 (85.8–92.9)	7.4	13.8	19.5	24.7	29.2	99.8	99.6	99.3	99.1	98.9
**Specificity 95%**	58.9	67.5 (62.4–72.3)	42.0 (34.5–49.7)	90.6 (85.6–94.3)	95.0 (92.0–97.2)	12.1	21.8	29.7	36.2	41.8	99.7	99.3	99.0	98.6	98.2
**AFP-L3**															
	**AFP-L3**	**Sensitivity all-stage**	**Sensitivity early-stage (BCLC 0/A)**	**Sensitivity late-stage (BCLC B–D)**	**Specificity**	**PPV 1%**	**PPV 2%**	**PPV 3%**	**PPV 4%**	**PPV 5%**	**NPV 1%**	**NPV 2%**	**NPV 3%**	**NPV 4%**	**NPV 5%**
**Specificity 70%**	1.2	100 (99.0–100)	100 (97.9–100)	100 (98.1–100)	0 (0–1.22)	1	2	3	4	5	-	-	-	-	-
**Specificity 75%**	1.2	100 (99.0–100)	100 (97.9–100)	100 (98.1–100)	0 (0–1.22)	1	2	3	4	5	-	-	-	-	-
**Specificity 80%**	1.2	100 (99.0–100)	100 (97.9–100)	100 (98.1–100)	0 (0–1.22)	1	2	3	4	5	-	-	-	-	-
**Specificity 85%**	1.2	100 (99.0–100)	100 (97.9–100)	100 (98.1–100)	0 (0–1.22)	1	2	3	4	5	-	-	-	-	-

**Specificity 90%**	1.23	64.2 (59.1–69.1)	52.3 (44.6–59.9)	75 (68.3–81.0)	89.7 (85.7–92.9)	5.9	11.3	16.2	20.6	24.7	99.6	99.2	98.8	98.4	97.9
**Specificity 95%**	1.51	61.2 (56.0–66.2	47.7 (40.1–55.4)	73.4 (66.6–79.5)	94.4 (91.1–96.7)	9.9	18.1	25.1	31.1	36.3	99.6	99.2	98.7	98.3	97.9
**GAAD**															
	**GAAD**	**Sensitivity all-stage**	**Sensitivity early-stage (BCLC 0/A)**	**Sensitivity late-stage (BCLC B–D)**	**Specificity**	**PPV 1%**	**PPV 2%**	**PPV 3%**	**PPV 4%**	**PPV 5%**	**NPV 1%**	**NPV 2%**	**NPV 3%**	**NPV 4%**	**NPV 5%**
**Specificity 70%**	0.654	95.6 (93.0–97.5)	92.5 (87.6–96.0)	98.4 (95.5–99.7)	70.3 (64.8–75.4)	3.2	6.2	9.1	11.8	14.5	99.9	99.9	99.8	99.7	99.7
**Specificity 75%**	0.778	93.7 (90.7–96.0)	88.5 (82.8–92.8)	98.4 (95.5–99.7)	74.9 (69.6–79.7)	3.6	7.1	10.4	13.5	16.4	99.9	99.8	99.7	99.7	99.6
**Specificity 80%**	1.01	90.7 (87.3–93.5)	82.8 (76.3–88.1)	97.9 (94.8–99.4)	79.9 (74.9–84.2)	4.4	8.4	12.2	15.8	19.2	99.9	99.8	99.6	99.5	99.4
**Specificity 85%**	1.21	89.6 (86.0–92.5)	81.0 (74.4–86.6)	97.4 (94.0–99.1)	85.1 (80.6–89.0)	5.8	11.0	15.7	20.1	24.1	99.9	99.8	99.6	99.5	99.4
**Specificity 90%**	2.0	84.7 (80.6–88.2)	72.4 (65.1–78.9)	95.8 (92.0–98.2)	89.8 (85.8–92.9)	7.7	14.5	20.4	25.6	30.3	99.8	99.7	99.5	99.3	99.1
**Specificity 95%**	2.83	82.5 (78.2–86.3)	69.0 (61.5–75.7)	94.8 (90.6–97.5)	94.7 (91.6–97.0)	13.6	24.2	32.6	39.4	45.1	99.8	99.6	99.4	99.2	99.0
**GALAD**															
	**GALAD**	**Sensitivity all-stage**	**Sensitivity early-stage (BCLC 0/A)**	**Sensitivity late-stage (BCLC B–D)**	**Specificity**	**PPV 1%**	**PPV 2%**	**PPV 3%**	**PPV 4%**	**PPV 5%**	**NPV 1%**	**NPV 2%**	**NPV 3%**	**NPV 4%**	**NPV 5%**

**Specificity 70%**	0.655	95.4 (92.7–97.3)	92.0 (86.9–95.5)	98.4 (95.5–99.7)	69.8 (64.2–74.9)	3.1	6.1	8.9	11.6	14.2	99.9	99.9	99.8	99.7	99.7
**Specificity 75%**	0.797	94.0 (91.0–96.2)	89.1 (83.5–93.3)	98.4 (95.5–99.7)	75.1 (69.8–79.9)	3.7	7.2	10.4	13.6	16.6	99.9	99.8	99.8	99.7	99.6
**Specificity 80%**	1.04	90.7 (87.3–93.5)	83.3 (76.9–88.5)	97.4 (94.0–99.1)	80.1 (75.1–84.4)	4.4	8.5	12.3	15.9	19.3	99.9	99.8	99.6	99.5	99.4
**Specificity 85%**	1.22	89.9 (86.3–92.8)	81.6 (75.0–87.1)	97.4 (94.0–99.1)	85.0 (80.5–88.9)	5.7	10.9	15.7	20.0	24.0	99.9	99.8	99.6	99.5	99.4
**Specificity 90%**	1.82	86.6 (82.7–89.9)	76.4 (69.4–82.5)	95.8 (92.0–98.2)	90.0 (86.1–93.2)	8.1	15.1	21.2	26.6	31.4	99.9	99.7	99.5	99.4	99.2
**Specificity 95%**	2.83	82.0 (77.6–85.8)	67.8 (60.3–74.7)	94.8 (90.6–97.5)	95.0 (91.9–97.2)	14.2	25.1	33.7	40.7	46.4	99.8	99.6	99.4	99.2	99.0

AFP, alpha-fetoprotein; AFP-L3, *Lens culinaris* agglutinin-reactive AFP; BCLC, Barcelona Clinic Liver Cancer; DCP, des-gamma carboxyprothrombin (PIVKA-II); GAAD, gender (biological sex), age, AFP, DCP; GALAD, gender (biological sex), age, AFP-L3, AFP, DCP; HCC, hepatocellular carcinoma; NPV, negative predictive value; PIVKA-II, protein induced by vitamin K absence or antagonist II; PPV, positive predictive value.
